# Provider Volume Impacts Neurosurgical Procedure Selection in Older Patients With High‐Grade Glioma

**DOI:** 10.1002/cam4.70866

**Published:** 2025-04-18

**Authors:** Anne S. Reiner, Akriti Mishra Meza, Katherine S. Panageas, Nelson S. Moss

**Affiliations:** ^1^ Department of Epidemiology and Biostatistics Memorial Sloan Kettering Cancer Center New York New York USA; ^2^ Department of Neurosurgery Memorial Sloan Kettering Cancer Center New York New York USA

**Keywords:** astrocytoma, glioma, Medicare, population‐based, SEER

## Abstract

**Background:**

We examined the association between academic center status and neurosurgical resection volume with surgical procedures performed and subsequent survival.

**Methods:**

In a population‐based study using the Surveillance, Epidemiology, and End Results (SEER)‐Medicare‐linked databases, we identified patients > 65 years diagnosed with primary WHO grade III‐IV glioma from 2008 to 2017. Surgical procedures were identified through Medicare claims from 2007 to 2019. Associations between center type (academic vs. not) and center volume (top 10% of distribution of resections during the study period vs. the bottom 90%) were estimated with upfront surgery procedure (resection vs. biopsy vs. none) and survival by estimating hazard ratios (HRs) and 95% confidence intervals (CIs) from multivariable regression models accounting for within‐center provider cluster correlation.

**Results:**

We identified 8592 patients, of whom 8128 could both be attributed to a provider and received neurosurgical intervention attributed to resection or biopsy. When considered together, center volume, not center academic status, drove surgical decisions for first procedure type such that patients treated by a top 10% volume center were 23% more likely to receive resection (95% CI: 14%–34%, *p* < 0.0001). When considered together, resection, not center volume, drove improvement in overall survival such that patients who received resection, regardless of center volume, were 22% less likely to die during the study period (95% CI: 17%–27%, *p* < 0.0001).

**Conclusions:**

We provide the first population‐based evidence that older patients diagnosed with grade III–IV glioma who seek treatment from higher‐volume centers are more likely to receive aggressive neurosurgical care. Aggressive neurosurgical care, even if received from low‐volume centers, improves survival.

**Abbreviations**
CI: confidence interval
HR: hazard ratio
IDH: isocitrate dehydrogenase
NCCN: National Comprehensive Cancer Network
OS: overall survival
RT: radiation therapy
SD: standard deviation
SEER: surveillance, epidemiology, and end results

## 1 Introduction

High‐grade gliomas, encompassing grade III and IV tumors, constitute approximately 60% of all malignant brain tumors in the United States [1, 2]. Initial therapeutic management for newly diagnosed patients relies heavily on surgery [3]. Furthermore, achieving favorable outcomes for patients with glioblastoma depends on the implementation of aggressive surgical management, with a higher extent of resection associated with improved survival, including in older patients [4, 5, 6, 7, 8, 9].

Delivering appropriate surgical care demands a well‐coordinated surgical system comprised of an interdependent network of primary healthcare settings, community hospitals, and specialty centers resourced with skilled surgeons, and patients able to reach the appropriate care in a timely fashion [10, 11, 12]. However, the landscape of neuro‐oncologic and neurosurgical care in the United States is highly fragmented, influenced by an interplay of socioeconomics, geographic location, race, ethnicity, and access to care [10, 13, 14]. A geospatial analysis of the neurosurgical workforce across the United States highlights concentrations of neurosurgeons at academic centers, which are also disproportionately situated in urban settings [13]. Patients who receive care from high‐volume or academic centers with specialized expertise tend to experience improved surgical outcomes; however, the role of academic setting versus surgeon experience in driving surgical aggressiveness and/or outcomes remains uncertain [15, 16, 17]. In addition, while volume correlates of treatment including resection have been described, such associations with choice of invasive procedure (namely diagnostic biopsy versus resection) have not been evaluated [18].

Using the 2020 release of SEER‐Medicare data, we aimed to describe contemporary patterns of surgery and survival and investigate the effects of both center type (academic vs. not) and center volume on these outcomes among older patients with high‐grade gliomas. We hypothesized that not presenting to an academic center or a higher‐volume center limits the degree of neurosurgical care received.

## 2 Methods

### 2.1 Design

A population‐based cohort study was implemented using data from Surveillance, Epidemiology, and End Results (SEER) linked with Medicare claims from 2007 through 2019. The SEER registries include over 25% of all patients diagnosed with cancer in the United States. Medicare is the primary health insurer for Americans 65 years and older and covers medical care including, but not limited to, inpatient hospital treatment, outpatient care, and physician services. The SEER‐Medicare‐linked database is a unique resource providing a large population‐based cohort which can be used to longitudinally evaluate health outcomes [19]. The Memorial Sloan Kettering institutional review board deemed this study (IRB #X23‐027) exempt from review and waived the need for informed consent.

### 2.2 Cancer Cohort

Our cohort was established using the SEER‐Medicare‐linked databases to identify diagnoses from January 1, 2008, to December 31, 2017. Medicare eligibility is primarily based on being 65 years or older (individuals under 65 may also quality if they have certain disabilities or end‐stage renal disease). For our cohort, eligibility was specifically defined by individuals > 65 years at the time of their primary diagnosis of glioma. Data on isocitrate dehydrogenase (IDH) mutation status, O6‐methylguanine‐DNA methyltransferase (MGMT) promoter methylation status, and 1p19q information were not available. We identified patients diagnosed with a first primary brain cancer diagnosis using WHO 2008 diagnostic criteria of astrocytoma or glioblastoma identified from the SEER file utilizing the primary site recode ICD‐O‐3/WHO 2008 “site_recode_icd_o_3_who_2008” variable (codes: 31010 and 31040). Furthermore, patients diagnosed with astrocytoma or glioblastoma were identified with “histologic_type_icd_o_3” codes 9401 and 9440, respectively. Exclusions were applied if any of the following occurred: Patient lacked Part A or B Medicare coverage or belonged to a health maintenance organization in the year prior to primary glioma diagnosis or through follow‐up; primary glioma diagnosis was made only at the time of death; month of primary glioma diagnosis was missing; primary glioma diagnosis was made before age 66. Requiring the primary glioma diagnosis to be made when the patient was > 65 years of age allowed us to ascertain comorbidity status in the year prior to primary glioma diagnosis.

Sociodemographic characteristics, including sex, year of glioma diagnosis, marital status, cancer grade, geographic region, and census tract poverty level, were available in the SEER file, as were tumor characteristics including size and laterality. Race and date of birth to determine age at glioma diagnosis were obtained from the Medicare enrollment database.

### 2.3 Claims

We utilized Medicare claims from January 1, 2007, through December 31, 2019, from the following files: physician and supplier, outpatient standard analytic, inpatient visits, provider analysis and review, home health agencies, hospice visits, durable medical equipment, and part D data. Claims information was available through December 31, 2019, which was the end of follow‐up. Claims information was also queried from the calendar year 2007, a year prior to the cohort start year of 2008, to ascertain comorbidities and calculate the Charlson comorbidity score for the year prior to glioma diagnosis, an index shown to have excellent clinimetric properties [20, 21]. Claim codes used for surgery, radiotherapy, and chemotherapy are available in Tables S1–S4.

### 2.4 Hospital Characteristics

The hospital characteristics file was used to ascertain academic status for medical facilities where patients received surgical interventions through 2016 using the variable “academic hospital (F38)” (yes/no). For patients with hospital characteristics information unavailable for the same year as their primary glioma diagnosis, hospital characteristics information from the year prior was used, and in rare cases when hospital characteristics were unavailable from the prior year, then hospital characteristics’ information from 2 years prior was used.

### 2.5 Center Volume

Center provider volume was determined by totaling the number of resections performed at each unique center over the study timeframe (2007–2019). Centers were assigned with the following algorithm: For patients with a surgical procedure (either resection or biopsy), the center for the procedure was assigned; for patients without a surgical procedure or for patients with a surgical procedure without a corresponding center, the center for the diagnostic scan in closest absolute time to the glioma diagnosis was assigned; for patients with multiple centers for either the surgical procedure or the diagnostic scan, the center associated with an inpatient claim was prioritized.

### 2.6 Statistical Analyses

Statistical measures such as means, medians, ranges, and proportions were used to characterize the cohort under study overall and by glioma subtype. Published definitions of high‐volume surgical centers vary widely for general surgery and neurosurgery [22, 23, 24, 25]. We defined high volume using the top decile of center neurosurgical volume, which is in the range of published literature and also allowed us to investigate the interaction between center volume and center academic status. Center volume was dichotomized (top 10%/bottom 90%). The correlation between volume and academic status was tested using the chi‐squared test. Rates of surgery by type were described using cumulative incidence rates with corresponding 95% confidence intervals (CI) and were estimated separately for resection and biopsy, considering the presence of competing risks with death as a competing event. Cumulative incidence curves for upfront surgery type (biopsy versus resection) were estimated, stratified by center academic status and center volume. Associations between receipt of surgery type with center academic status, center volume, and the interaction between center academic status and center volume were estimated using multivariable competing risks regression modeling accounting for within‐center provider cluster correlation to determine their individual and joint effects on surgical decision. When investigating the association between center academic status and receipt of surgery type, center volume was not used as an adjusting variable. Similarly, when investigating the association between center volume and receipt of surgery type, center academic status was not used as an adjusting variable.

Kaplan–Meier methodology was used to describe overall survival (OS) from the date of primary glioma diagnosis until death for those who died prior to administrative follow‐up (12/31/2019) or until the last administrative claim date (12/31/2019) for those who were alive. Survival curves were estimated separately by center type and center volume. The impact of resection on overall survival, as well as the interaction between center volume and resection on survival, was assessed using multivariable Cox regression accounting for within‐center cluster correlation and with resection treated as a time‐varying covariate.

Multivariable models were adjusted for several factors, including age at first primary glioma diagnosis, year of first primary glioma diagnosis, tumor size, tumor laterality, sex, race, marital status, geographic registry region, census tract‐based poverty, glioma subtype, time‐varying treatments (chemotherapy and radiation), and Charlson Comorbidity Index in the year prior to glioma diagnosis. Resection was also adjusted for in the OS models. All tests were two‐sided with an alpha level of statistical significance < 0.05. Analyses were performed using SAS version 9.4 (the SAS Institute, Cary, NC) and R v4.2.1 (R Foundation for Statistical Computing).

## 3 Results

### 3.1 Patient and Provider Characteristics

We identified 8592 patients diagnosed over 65 years of age with primary anaplastic astrocytoma (6%) or glioblastoma (94%) from 2008 to 2017. Most patients were white (90%) and male (54%) (Table 1). Of 8592 patients, 464 either could not be attributed to a center or received neurosurgical intervention which could not be classified as resection or biopsy, resulting in an analytical cohort of 8128 patients. There were 810 unique providers who performed a median of 7 resections from 2007 to 2019 (range: 0–841). The top 10% performed 68 or more resections during the study timeframe (Figure S1). In general, patients who were treated at high‐volume centers were younger, lived in areas with lower census tract poverty, and were married. There were no appreciable differences in race or sex by center volume.

**TABLE 1:** Cohort characteristics.

Variable	Category	All^a^	Glioblastoma	Grade 3 astrocytoma
		*N* (%)	*N* (%)	*N* (%)
First surgery^b^	None or surgical procedure uncategorized	1529 (18)	1470 (18)	59 (12)
	Biopsy	2207 (26)	1935 (24)	272 (54)
	Resection	4856 (57)	4686 (58)	170 (34)
Center type	Academic	6161 (72)	5793 (72)	368 (73)
	Nonacademic	1967 (23)	1851 (23)	116 (23)
	Uncategorized	464 (5)	447 (6)	17 (3)
Center volume	Low volume or uncategorized	4588 (53)	4327 (53)	261 (52)
	High volume	4004 (47)	3764 (47)	240 (48)
Comorbidity Index	0	1056 (12)	944 (12)	62 (12)
	1	1607 (19)	1500 (19)	107 (21)
	≥ 2	5929 (69)	5597 (69)	332 (66)
Age at diagnosis, years	66–69	2164 (25)	2027 (25)	137 (27)
	70–74	2328 (27)	2190 (27)	138 (28)
	75–79	1890 (22)	1765 (22)	125 (25)
	80–84	1364 (16)	1294 (16)	70 (14)
	≥ 85	846 (10)	815 (10)	31 (6)
Geographic location	West	2909 (34)	2722 (34)	187 (37)
	Midwest	707 (8)	657 (8)	50 (10)
	South	1480 (17)	1396 (17)	84 (17)
	Northeast	3496 (41)	3316 (41)	180 (36)
Marital status	Married	3843 (45)	3613 (45)	230 (46)
	Unmarried	2220 (26)	2088 (26)	132 (26)
	Unknown	2529 (29)	2390 (30)	139 (28)
Census tract poverty level	≥ 10%	4236 (49)	3985 (49)	242 (48)
Race	White	7771 (90)	7316 (90)	455 (91)
	Black	294 (3)	274 (3)	20 (4)
	Other/Unknown	527 (6)	501 (6)	26 (5)
Sex	Male	4617 (54)	4365 (54)	252 (50)
	Female	3975 (46)	3726 (46)	249 (50)
Year of diagnosis	2008–2010	2533 (29)	2398 (30)	135 (27)
	2011–2013	2583 (30)	2435 (30)	148 (30)
	2014–2017	3476 (40)	3258 (40)	218 (44)

^a^ Agreement with SEER‐Medicare does not allow the reporting of results for fewer than 11 patients due to privacy. Some cells are omitted entirely or collapsed/combined to circumvent back calculation of reportable patients.

^b^ Within 3 months of glioma diagnosis.

### 3.2 Treatment Patterns

**FIGURE 1:**
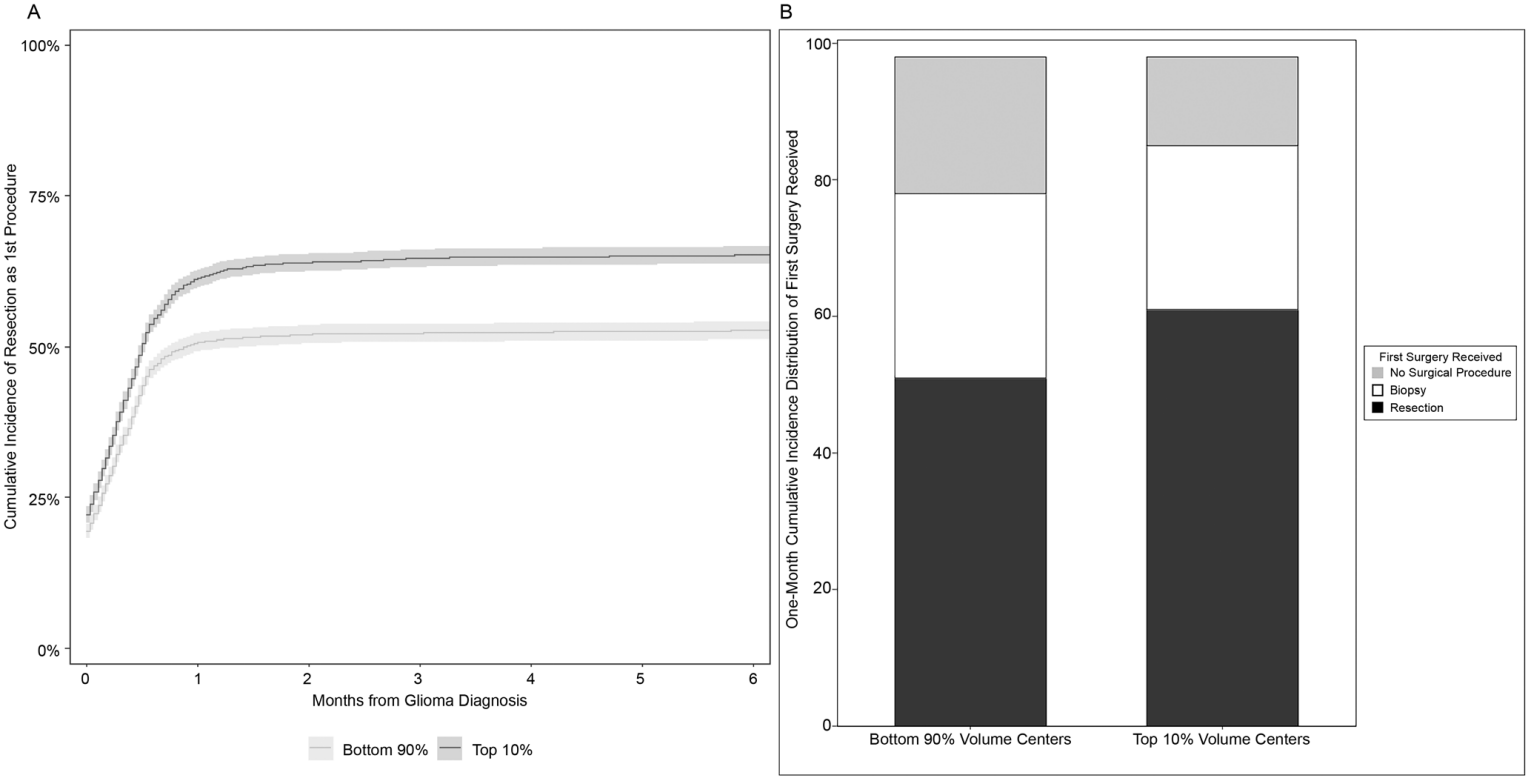
(A) Cumulative incidence of upfront surgery in older patients with high‐grade glioma, stratified by center volume (low volume: Bottom 90% vs. high volume: Top 10%). (B) Histogram of 1‐month cumulative incidence of surgical procedures stratified by center volume.

Using cumulative incidence to account for the competing risk of death, the overall 6‐month incidence of resection was 59% (95% CI: 58%–60%) (Table 2). When stratified by center volume, the 6‐month incidence of resection was 65% for patients treated by top 10% volume centers (95% CI: 64%–67%) and 53% for patients treated by bottom 90% volume centers (95% CI: 51%–54%) (Figure 1A). By center academic status, the 6‐month incidence of resection was 61% for patients treated at academic medical facilities (95% CI: 60%–62%) and 52% for patients treated at nonacademic medical facilities (95% CI: 50%–55%). The distribution of 1‐month cumulative incidence of resection as the first surgical procedure, biopsy as the first surgical procedure, and no surgical procedure, stratified by center volume, is displayed in Figure 1B. Volume and academic center status were highly correlated (*p* < 0.0001, data not shown). In a multivariable model that did not adjust for center volume, academic center status was not statistically significantly associated with resection as the first procedure (HR: 1.09, 95% CI: 0.999–1.20, *p* = 0.09) (data not shown). Furthermore, in the multivariable model assessing the interaction of center academic status and center volume on the probability of resection, there was no association of center academic status with resection for either bottom 90% or top 10% volume centers. However, there was a statistically significant association between top 10% volume centers and the probability of resection for both academic and nonacademic centers. Thus, academic status was removed as a variable of interest and center volume was pursued further. In our final multivariable model adjusting for the full complement of potential confounders, top 10% volume centers maintained an increased association of providing upfront resection compared with bottom 90% volume centers (HR: 1.23; 95% CI: 1.14–1.34) (Table 3). Other expected associations were also identified, including older patients less likely to receive resection and patients with the highest grade glioma more likely to receive resection.

**TABLE 2:** Cumulative incidence of upfront resection.

Population	6‐month cumulative incidence of upfront resection (%)	95% confidence interval
Overall	59	58%–60%
Patients at a top 10% volume center	65	64%–67%
Patients at a bottom 90% volume center	53	51%–54%
Patients at an academic center	61	60%–62%
Patients at a nonacademic center	52	50%–55%

**TABLE 3:** Multivariable predictors of resection in the overall cohort.

Variable	Category	Modeling resection, multivariable^a^	
		HR^a^ (95% CI)	*p* ^a^
Center volume	Bottom 90%	Ref.	Ref.
	Top 10%	1.23 (1.14, 1.34)	< 0.0001
Comorbidity Index	0	Ref.	Ref.
	1	1.08 (0.98, 1.20)	0.13
	2	1.14 (1.04, 1.26)	0.005
	≥ 3	1.20 (1.10, 1.32)	< 0.0001
Age at diagnosis, years	66–69	Ref.	Ref.
	70–74	0.94 (0.88, 1.01)	0.11
	75–79	0.84 (0.78, 0.91)	< 0.0001
	80–84	0.68 (0.62, 0.75)	< 0.0001
	≥ 85	0.37 (0.33, 0.43)	< 0.0001
Marital status	Married	Ref.	Ref.
	Unmarried	0.96 (0.90, 1.02)	0.20
	Unknown	1.03 (0.92, 1.16)	0.60
Census tract poverty level	< 10%	Ref.	Ref.
	≥ 10%	0.97 (0.92, 1.04)	0.39
	Unknown	0.94 (0.78, 1.13)	0.50
Race	White	Ref.	Ref.
	Black	1.02 (0.87, 1.19)	0.85
	Other/Unknown	0.92 (0.82, 1.02)	0.12
Sex	Female	Ref.	Ref.
	Male	1.00 (0.94, 1.06)	0.91
Geographic location	South	Ref.	Ref.
	West	1.12 (0.996, 1.26)	0.06
	Midwest	1.14 (0.98, 1.32)	0.10
	Northeast	1.30 (1.14, 1.49)	< 0.0001
Histology	Grade IV	Ref.	Ref.
	Grade III	0.46 (0.40, 0.54)	< 0.0001

Abbreviations: CI, confidence interval; HR, hazard ratio; Ref, reference.

^a^ Adjusted for comorbidity index in the year prior to glioma diagnosis, provider volume, age at glioma diagnosis, glioma subtype, glioma size, glioma laterality, geographic location, marital status, census tract poverty level, race, sex, radiation treatment (time‐dependent), and chemotherapy (time‐dependent).

There was no association between the main effects of center academic status or center volume, or the interactive effects of center academic status and provider volume on the receipt of biopsy (data not shown).

### 3.3 Overall Survival

Median overall survival was 5.5 months (95% CI: 5.3–5.7) for the overall cohort, 7.1 months (95% CI: 5.9–8.1) for 484 patients with anaplastic astrocytoma, and 5.4 months (95% CI: 5.2–5.6) for 7644 patients with glioblastoma. Survival varied by center volume such that the median OS for patients treated at bottom 90% volume centers was 4.5 months (95% CI: 4.3–4.7) and 6.9 months (95% CI: 6.5–7.1) for patients treated at top 10% volume centers. Patients first treated at an academic medical facility had a median OS of 6.0 months (95% CI: 5.8–6.3) compared to patients first treated at a nonacademic medical facility with a median OS of 4.4 months (95% CI: 4.1–4.6). In the multivariable model assessing the interaction between center volume and resection on overall survival, there was no interaction (*p* = 0.84) such that patients receiving resection, regardless of center volume, were less likely to die during the study period (HR: 0.78. 95% CI: 0.73–0.83, *p* < 0.0001) (Table 4). Other expected associations were also identified during the study period, including younger patients more likely to survive, patients with grade III glioma more likely to survive, and patients receiving either chemotherapy or radiation therapy more likely to survive.

**TABLE 4:** Multivariable predictors of survival in the overall cohort.

Variable	Category	Modeling survival, multivariable^a^	
		HR^a^ (95% CI)	*p* ^a^
Center volume	Bottom 90%	Ref.	Ref.
	Top 10%	0.95 (0.89, 1.02)	0.15
Comorbidity Index	0	Ref.	Ref.
	1	1.10 (0.99, 1.23)	0.08
	2	1.15 (1.03, 1.29)	0.01
	≥ 3	1.40 (1.27, 1.55)	< 0.0001
Age at diagnosis, years	66–69	Ref.	Ref.
	70–74	1.10 (1.02, 1.18)	0.009
	75–79	1.28 (1.18, 1.38)	< 0.0001
	80–84	1.55 (1.41, 1.70)	< 0.0001
	≥ 85	1.52 (1.33, 1.74)	< 0.0001
Marital status	Married	Ref.	Ref.
	Unmarried	1.07 (0.99, 1.14)	0.08
	Unknown	0.94 (0.85, 1.03)	0.18
Census tract poverty level	< 10%	Ref.	Ref.
	≥ 10%	1.12 (1.06, 1.18)	0.0002
	Unknown	1.26 (1.07, 1.48)	0.006
Race	White	Ref.	Ref.
	Black	0.75 (0.64, 0.89)	0.001
	Other/Unknown	0.77 (0.68, 0.88)	0.0001
Sex	Female	Ref.	Ref.
	Male	1.03 (0.98, 1.08)	0.31
Geographic location	South	Ref.	Ref.
	West	0.93 (0.83, 1.03)	0.14
	Midwest	1.05 (0.92, 1.20)	0.44
	Northeast	1.00 (0.90, 1.12)	0.98
Histology	Grade IV	Ref.	Ref.
	Grade III	0.78 (0.70, 0.87)	< 0.0001
Chemotherapy^b^	No	Ref.	Ref.
	Yes (time‐dependent)	0.50 (0.47, 0.53)	< 0.0001
Radiation therapy^b^	No	Ref.	Ref.
	Yes (time‐dependent)	0.44 (0.41, 0.48)	< 0.0001
Surgical resection^b^	No	Ref.	Ref.
	Yes (time‐dependent)	0.78 (0.73, 0.83)	< 0.0001

Abbreviations: CI, confidence interval; HR, hazard ratio; Ref, reference.

^a^ Adjusted for comorbidity index in the year prior to glioma diagnosis, provider volume, age at glioma diagnosis, glioma subtype, glioma size, glioma laterality, geographic location, marital status, census tract poverty level, race, sex, surgical resection (time‐dependent), radiation treatment (time‐dependent), and chemotherapy (time‐dependent).

^b^ Time‐dependent variable.

## 4 Discussion

Utilizing population‐based SEER‐Medicare data, we identified a significant increase (23%) in the likelihood of older patients with high‐grade glioma undergoing a resection when they were treated by higher‐volume providers. However, the survival benefit conferred by resection did not vary by center volume. In a previous cohort study using SEER‐Medicare data of incident cancers in the pancreas, esophagus, lung, colon, rectum, and genitourinary tract, Begg et al. reported an association between low hospital volume and increased mortality overall and, notably, with a quintupling in the rate of operative mortality for esophagectomy [15]. Similarly, Birkmeyer and colleagues highlighted surgical outcome disparities due to variations in socioeconomic status which were largely attributable to hospital differences [16]. Using the Nationwide Inpatient Sample of patients with an intracranial tumor resection, Cowan et al. demonstrated superior surgical resection outcomes for higher‐volume providers [17]. In a recent study by Zhu and colleagues using the national cancer database, they demonstrated that patients with glioblastoma treated at academic centers were more likely to undergo upfront resection, and that upfront resection was associated with improved 30‐ and 90‐day mortality, though the cohort only included patients with glioblastoma, and they did not investigate center volume [26]. Notably, in our study, though volume and academic center status were highly correlated, academic center status was not associated with the first surgical procedure, but volume was. Our findings underscore that high‐volume centers are more inclined to perform resections on high‐grade gliomas, a factor known to correlate with improved prognosis. In contrast, low‐volume centers were more likely to undertreat surgically, either by performing biopsy exclusively or not performing any surgical intervention.

Interestingly, biopsy rates were similar across different care settings, a finding of particular interest given that biopsies are conventionally recommended for tumors deemed unresectable. This suggests two potential implications: (1) Patients with tumors potentially amenable to resection may have been deprived of access to a potentially feasible resective operation by opting for a lower‐volume provider, and (2) patients who forewent biopsy may have become ineligible for nonsurgical treatments requiring pathologic diagnosis (such as standard‐of‐care chemotherapy and/or radiation) despite the prevalence of biopsy procedures in community hospital settings. Previous reports from 2003, 2007, and 2022 demonstrated that older patients with malignant glioma experienced a prognostic benefit from surgical resection [6, 27, 28]. In a prior SEER‐Medicare report on older patients with glioblastoma, we found that older age was associated with a smaller likelihood of receiving standard‐of‐care treatment [29]. Furthermore, in another prior SEER‐Medicare report, we reported that only 40% of older patients with lower‐grade glioma received surgery [6]. Alongside the reduced likelihood of receiving appropriate surgical care, others have shown that these same older patients with brain tumors are less likely to be admitted to high‐volume hospitals and less likely to have access to complex neuro‐oncologic specialty care [14].

A well‐documented shortage of neurosurgeons exists in the United States, which is further exacerbated by inefficiencies in distribution [10, 30, 31]. This challenge was highlighted more than a decade ago, midway through the current cohort diagnosis period, when Rosman et al. determined that current job openings for neurosurgeons would take over 2 years to fill and anticipated that this shortage would persist and likely worsen [30]. Complex neurosurgical oncologic procedures, including glioma resection, constitute a small minority of the overall volume of neurosurgical procedures, and the prevailing trends toward subspecialization have given rise to an increasing population of neurosurgeons with a focus on spine‐related procedures, further intensifying this imbalance. This trend is evident even among academic neurosurgeons, with fellowship training in spinal surgery experiencing a 6.3‐fold increase in recent decades, reaching 18% of all faculty, while neurosurgical oncology‐trained surgeons have remained relatively constant (1.3‐fold difference, comprising approximately 10% of faculty) [32].

Although comparable data for community hospitals are lacking, it is highly likely that the density of surgeons proficient in glioma surgery is significantly lower in these settings despite their contribution to over 80%–90% of all surgical care [12]. Glioma surgeries require a high level of surgical expertise, encompassing skills like preoperative analysis (such as functional MRI, transcranial magnetic stimulation, and tractography), intraoperative electrophysiology (awake and asleep mapping), and intraoperative imaging (MRI, ultrasonography, and fluorophore guidance) to ensure safe and effective resection. Furthermore, a specialized ecosystem is essential, including 24‐h surgical and critical care coverage to manage potential complications. Close multidisciplinary coordination also likely facilitates timely adjuvant treatments and any needed rehabilitation services.

The optimization of patient access to high‐quality surgical neuro‐oncologic care is highly context‐dependent. For patients with access to specialized centers (importantly, without financial and geographic barriers), ensuring timely referral is of paramount importance. Unfortunately, for many patients, such access is limited owing to geography (leading to rural–urban outcome disparities), transportation, caregiver support, and other socioeconomic constraints for both patients and caregivers including employment insecurity, leading to healthcare disparities in neuro‐oncologic care [33, 34, 35]. Strategies for workforce improvement allowing broader access to higher‐level care include telemedicine and collaborative medicine tools including “hub‐and‐spoke” care models [36, 37]. These tools facilitate communication among moderately experienced surgeons with specialist centers who may provide advice and guidance on case management, the development of visiting specialist teams, or models to incentivize surgeon service in nonacademic settings, similar to approaches advocated for national surgical systems in low/middle‐income countries [12]. Such models might involve training initiatives in more rural areas or adjustments to payment schemes.

Further research is needed to explore whether disparities in procedure rates are related to deferred biopsies for patients who are considered suitable candidates for resection but who fail to access this care; for patients who would prefer not to or are unable to travel for resection, enhanced communication among primary/community care providers with patients and specialty surgeons may identify patients who would benefit from biopsy as an intermediate diagnostic option, even if suboptimal. This requires further exploration to ascertain its feasibility and effectiveness.

Our study has certain limitations that warrant consideration. While the linked SEER‐Medicare databases provide comprehensive data, they do not include information on performance status or molecular markers that can guide treatment decisions and affect prognosis. Molecular markers for CNS tumors have only recently been incorporated into SEER registry data. Research utilizing future releases of SEER‐Medicare‐linked data will enable more robust population‐based studies on molecularly defined CNS tumor types [38]. The distribution of resectable tumors across centers may be influenced by a bias, wherein patients initially present with signs indicative of a malignant brain tumor. However, the subjective nature of the anatomic bounds and the frequent lack of correlation between tumor resectability and symptom severity help mitigate this bias. Furthermore, we accounted for medical factors affecting surgical candidacy. We did not ascertain the distribution of neurosurgical procedures relevant to high‐grade glioma patients who were not Medicare beneficiaries. Our assumption was that centers defined as top 10% volume centers or bottom 90% volume centers for patients with high‐grade glioma who were Medicare beneficiaries would not appreciably change when including patients with high‐grade glioma who were not Medicare beneficiaries. We recognize the potential for misclassification and coding omissions inherent in claims‐based research. However, prior literature demonstrates high accuracy and completeness of Medicare claims for surgical treatments of breast cancer as well as high sensitivity and specificity for brain metastasis surgery [39, 40]. We did not have access to the number of neurosurgeons at each center, but we assumed that most centers would not employ more than a few neurosurgeons. Our approach to identifying claims relating to adjuvant treatments was intentionally broad, drawing from prior research where attempts to capture specific treatment details were unsuccessful [6]. These results are, by design, applicable only to patients aged 66 years and older, a demographic that harbors the majority of glioblastoma [41]. We did not consider whether a center identified in our study as providing surgical treatment was in a SEER area. However, our results come from national population‐based data that cover approximately 35% of the US population and, arguably, are representative of the US population who is 66 years and older. In addition, 93% of SEER patients over age 65 have linked Medicare claims data, suggesting our findings are robust and widely applicable to the studied population [19].

## 5 Conclusions

Utilizing the linked SEER‐Medicare database, we characterized surgical patterns and prognosis of older patients with high‐grade glioma using a population‐based study design. We found that high‐volume centers and providers were more likely to treat patients with upfront resection. Strategies to expand access to experienced neurosurgical oncologic care to a broader proportion of the population may improve outcomes in high‐grade glioma.

## Author Contributions

Conceptualization and methodology were performed by Anne S. Reiner, Katherine S. Panageas, and Nelson S. Moss. Supervision was performed by Katherine S. Panageas and Nelson S. Moss. Katherine S. Panageas performed data acquisition. Formal analysis and visualization were performed by Akriti Mishra Meza. The first draft of the manuscript was written by Anne S. Reiner, and all authors commented on previous versions of the manuscript. All authors read and approved the final manuscript.

## Ethics Statement

The Memorial Sloan Kettering institutional review board deemed this study (IRB #X23‐027) exempt from review and waived the need for informed consent.

## Conflicts of Interest

Dr. Moss discloses research funding (to institution) from AstraZeneca and GT Medical Technologies, and consulting fees from AstraZeneca. All other authors declare they have no financial interests. Nonfinancial interests: All authors declare they have no nonfinancial interests.

## Supporting information


**Table S1.** Claims codes for surgery.
**Table S2.** Claims codes for chemotherapy.
**Table S3.** IV codes for systemic therapy drugs.
**Table S4.** Claims codes for radiation.
**Figure S1.** Distribution of Center Volume of Neurosurgical Procedure from 2007 to 2019. Outliers are not shown. Of the 810 providers—143 had more than 42 resections (across 2007–2019) and 66 had more than 25 biopsies (across 2007–2019).

## Data Availability

The datasets used to conduct this study are available upon approval of a research protocol from the National Cancer Institute. Instructions for obtaining these data are available at https://healthcaredelivery.cancer.gov/seermedicare/obtain.
