# Supplementary material for: Provider Volume Impacts Neurosurgical Procedure Selection in Older Patients With High‐Grade Glioma
Source: Cancer Med. 2025 Apr 18;14(8):e70866. doi: 10.1002/cam4.70866 (PMC12007465; doi:10.1002/cam4.70866)

Supplemental Table 1. Claims Codes for Surgery.

| Category | Code_Type | Code | Description |
| --- | --- | --- | --- |
| Craniotomy/Craniectomy | HCPCS/CPT | 61500 | Craniectomy; with excision of tumor or other bone lesion of skull |
| Craniotomy/Craniectomy | HCPCS/CPT | 61510 | Craniectomy, trephination, bone flap craniotomy; for excision of brain tumor, supratentorial, except meningioma |
| Craniotomy/Craniectomy | HCPCS/CPT | 61512 | Craniectomy, trephination, bone flap craniotomy; for excision of meningioma, supratentorial |
| Craniotomy/Craniectomy | HCPCS/CPT | 61516 | Craniectomy, trephination, bone flap craniotomy |
| Craniotomy/Craniectomy | HCPCS/CPT | 61518 | Craniectomy for excision of brain tumor, infratentorial or posterior fossa; except meningioma, cerebellopontine angle tumor, or midline tumor at base of skull |
| Craniotomy/Craniectomy | HCPCS/CPT | 61519 | Craniectomy for excision of brain tumor, infratentorial or posterior fossa; meningioma |
| Craniotomy/Craniectomy | HCPCS/CPT | 61520 | Craniectomy for excision of brain tumor, infratentorial or posterior fossa; cerebellopontine angle tumor |
| Craniotomy/Craniectomy | HCPCS/CPT | 61521 | Craniectomy for excision of brain tumor, infratentorial or posterior fossa; midline tumor at base of skull |
| Craniotomy/Craniectomy | HCPCS/CPT | 61524 | Craniectomy |
| Craniotomy/Craniectomy | HCPCS/CPT | 61526 | Craniectomy, bone flap craniotomy, transtemporal (mastoid) for excision of cerebellopontine angle tumor; |
| Craniotomy/Craniectomy | HCPCS/CPT | 61530 | Craniectomy, bone flap craniotomy, transtemporal (mastoid) for excision of cerebellopontine angle tumor; combined with middle/posterior fossa craniotomy/craniectomy |
| Craniotomy/Craniectomy | HCPCS/CPT | 61534 | Craniotomy with elevation of bone flap |
| Craniotomy/Craniectomy | HCPCS/CPT | 61536 | Craniotomy with elevation of bone flap |
| Craniotomy/Craniectomy | HCPCS/CPT | 61544 | Craniotomy with elevation of bone flap |
| Craniotomy/Craniectomy | HCPCS/CPT | 61545 | Craniotomy with elevation of bone flap |
| skull base procedures | HCPCS/CPT | 61580 | Craniofacial approach to anterior cranial fossa; extradural, including lateral rhinotomy, ethmoidectomy, sphenoidectomy, without maxillectomy or orbital exenteration |
| skull base procedures | HCPCS/CPT | 61581 | Craniofacial approach to anterior cranial fossa; extradural, including lateral rhinotomy, orbital exenteration, ethmoidectomy, sphenoidectomy and/or maxillectomy |
| skull base procedures | HCPCS/CPT | 61582 | Craniofacial approach to anterior cranial fossa; extradural, including unilateral or bifrontal craniotomy, elevation of frontal lobe(s), osteotomy of base of anterior cranial fossa |
| skull base procedures | HCPCS/CPT | 61583 | Craniofacial approach to anterior cranial fossa; intradural, including unilateral or bifrontal craniotomy, elevation or resection of frontal lobe, osteotomy of base of anterior cranial fossa |
| skull base procedures | HCPCS/CPT | 61584 | Orbitocranial approach to anterior cranial fossa, extradural, including supraorbital ridge osteotomy and elevation of frontal and/or temporal lobe(s); without orbital exenteration |
| skull base procedures | HCPCS/CPT | 61585 | Orbitocranial approach to anterior cranial fossa, extradural, including supraorbital ridge osteotomy and elevation of frontal and/or temporal lobe(s); with orbital exenteration |
| skull base procedures | HCPCS/CPT | 61586 | Bicoronal, transzygomatic and/or LeFort I osteotomy approach to anterior cranial fossa with or without internal fixation, without bone graft |
| skull base procedures | HCPCS/CPT | 61590 | auricular approach to middle cranial fossa (parapharyngeal space, infratemporal and midline skull base, nasopharynx), with or without disarticulation of the mandible, including parotidectomy, craniotomy, decompression and/or mobilization of the facial nerve and/or petrous carotid artery |
| skull base procedures | HCPCS/CPT | 61591 | auricular approach to middle cranial fossa (internal auditory meatus, petrous apex, tentorium, cavernous sinus, parasellar area, infratemporal fossa) including mastoidectomy, resection of sigmoid sinus, with or without decompression and/or mobilization of contents of auditory canal or petrous carotid artery |
| skull base procedures | HCPCS/CPT | 61592 | or intradural elevation of temporal lobe |
| skull base procedures | HCPCS/CPT | 61595 | Transtemporal approach to posterior cranial fossa, jugular foramen or midline skull base, including mastoidectomy, decompression of sigmoid sinus and/or facial nerve, with or without mobilization |
| skull base procedures | HCPCS/CPT | 61596 | Transcochlear approach to posterior cranial fossa, jugular foramen or midline skull base, including labyrinthectomy, decompression, with or without mobilization of facial nerve and/or petrous carotid artery |
| skull base procedures | HCPCS/CPT | 61597 | C3 vertebral body(s), decompression of vertebral artery, with or without mobilization |
| skull base procedures | HCPCS/CPT | 61598 | Transpetrosal approach to posterior cranial fossa, clivus or foramen magnum, including ligation of superior petrosal sinus and/or sigmoid sinus |
| Needle biopsy | HCPCS/CPT | 61750 | Stereotactic biopsy, aspiration, or excision, including burr hole(s), for intracranial lesion. |
| Needle biopsy | HCPCS/CPT | 61751 | Stereotactic biopsy, aspiration, or excision, including burr hole(s), for intracranial lesion. |
| Needle biopsy | HCPCS/CPT | 61736 | Laser interstitial thermal therapy (LITT) of lesion, intracranial, including burr hole(s), with magnetic resonance imaging guidance, when performed |
| Needle biopsy | HCPCS/CPT | 61737 | Under Stereotaxis Procedures on the Skull, Meninges, and Brain |
| Needle biopsy | HCPCS/CPT | 61140 | Burr hole(s) or trephine; with biopsy of brain or intracranial lesion |
| MRI | HCPCS/CPT | 70551 | Mri brain stem w/o dye |
| MRI | HCPCS/CPT | 70552 | Mri brain stem w/dye |
| MRI | HCPCS/CPT | 70553 | Mri brain stem w/o & w/dye |
| MRI | HCPCS/CPT | 70557 | Mri brain w/o dye |
| MRI | HCPCS/CPT | 70558 | Mri brain w/dye |
| MRI | HCPCS/CPT | 70559 | Mri brain w/o & w/dye |
| CT | HCPCS/CPT | 70450 | Ct head/brain w/o dye |
| CT | HCPCS/CPT | 70460 | Ct head/brain w/dye |
| CT | HCPCS/CPT | 70470 | Ct head/brain w/o & w/dye |
| PET | HCPCS/CPT | 78608 | BRAIN IMAGING, POSITRON EMISSION TOMOGRAPHY (PET); METABOLIC EVALUATION |
| PET | HCPCS/CPT | 78609 | BRAIN IMAGING, POSITRON EMISSION TOMOGRAPHY (PET); PERFUSION EVALUATION |
| Needle biopsy | ICD9_Proc | 01.13 | Closed [percutaneous] [needle] biopsy of brain |
| Needle biopsy | ICD10_Proc | 00903ZX | Drainage of Brain, Percutaneous Approach, Diagnostic |
| Needle biopsy | ICD10_Proc | 00904ZX | Drainage of Brain, Percutaneous Endoscopic Approach, Diagnostic |
| Needle biopsy | ICD10_Proc | 00B03ZX | Excision of Brain, Percutaneous Approach, Diagnostic |
| Needle biopsy | ICD10_Proc | 00B04ZX | Excision of Brain, Percutaneous Endoscopic Approach, Diagnostic |
| Needle biopsy | ICD10_Proc | 0W913ZX | Drainage of Cranial Cavity, Percutaneous Approach, Diagnostic |
| Needle biopsy | ICD10_Proc | 0W914ZX | Drainage of Cranial Cavity, Percutaneous Endoscopic Approach, Diagnostic |
| Not sure biopsy or resection | ICD9_Proc | 01.59 | BRAIN EXCISION/BIOPSY |
| Not sure biopsy or resection | ICD10_Proc | 00500ZZ | Destruction of Brain, Open Approach |
| Not sure biopsy or resection | ICD10_Proc | 00503ZZ | Destruction of Brain, Percutaneous Approach |
| Not sure biopsy or resection | ICD10_Proc | 00504ZZ | Destruction of Brain, Percutaneous Endoscopic Approach |
| Not sure biopsy or resection | ICD10_Proc | 00B00ZZ | Excision of Brain, Open Approach |
| Not sure biopsy or resection | ICD10_Proc | 00B03ZZ | Excision of Brain, Percutaneous Approach |
| Not sure biopsy or resection | ICD10_Proc | 00B04ZZ | Excision of Brain, Percutaneous Endoscopic Approach |

Supplemental Table 2. Claims Codes for Chemotherapy.

| Treatment Type | Code Type | Code | Description |
| --- | --- | --- | --- |
| Chemo | ICD9_proc | 99.25 | Injection or infusion of cancer chemotherapeutic substance |
| Chemo | ICD9_dx | V58.1 | Encounter for antineoplastic chemotherapy and immunotherapy |
| Chemo | ICD9_dx | V58.11 | Encounter for antineoplastic chemotherapy |
| Chemo | CPT | 36260 | Insertion of implantable intra-arterial infusion pump (eg, for chemotherapy of liver) |
| Chemo | CPT | 36640 | Arterial catheterization for prolonged infusion therapy (chemotherapy), cutdown |
| Chemo | CPT | 51720 | Bladder instillation of anticarcinogenic agent (including retention time) |
| Chemo | CPT | 96567 | Photodynamic therapy |
| Chemo | CPT | 96570 | Photodynamic therapy |
| Chemo | CPT | 96571 | Photodynamic therapy |
| Chemo | HCPCS | J8540 | Dexamethasone, oral, 0.25 mg |
| Chemo | HCPCS | J8562 | Fludarabine phosphate, oral, 10 mg |
| Chemo | HCPCS | J8565 | Gefitinib, oral, 250 mg |
| Chemo | HCPCS | J8600 | Melphalan, oral, 2 mg |
| Chemo | HCPCS | J8999 | Prescription drug, oral, chemotherapeutic, NOS |
| Chemo | HCPCS | C8953 | CHEMOTX ADM, IV PUSH |
| Chemo | HCPCS | C8954 | CHEMOTX ADM, IV INF UP TO 1H |
| Chemo | HCPCS | C8955 | CHEMOTX ADM, IV INF, ADDL HR |
| Chemo | ICD10_proc | 3E0K705 | Introduction of other antineoplastic into genitoutinary tract, via natural or artificial opening |
| Chemo | ICD10_proc | 3E0K805 | Introduction of other antineoplastic into genitoutinary tract, via natural or artificial opening endoscopic |
| Chemo | HCPCS | G0498 | Chemotherapy administration, intravenous infusion technique; initiation of infusion in the office/clinic setting using office/clinic pump/supplies, with continuation of the infusion in the community setting (e.g., home, domiciliary, rest home or assisted living) using a portable pump provided by the office/clinic, includes follow up office/clinic visit at the conclusion of the infusion |
| Chemo | ICD9_proc | 00.10 | INTRODUCTION OF OTHER ANTINEOPLASTIC INTO GU TRACT, ENDO |
| Chemo | ICD9_proc | 00.15 | High-dose infusion interleukin-2 |
| Chemo | ICD9_proc | 00.18 | Infusion of immunosuppressive antibody therapy |
| Chemo | HCPCS | C9257 | Injection, bevacizumab, 0.25 mg |
| Chemo | HCPCS | Q0083 | CHEMO BY OTHER THAN INFUSION |
| Chemo | HCPCS | Q0084 | CHEMOTHERAPY BY INFUSION |
| Chemo | HCPCS | Q0085 | CHEMO BY BOTH INFUSION AND O |
| Chemo | ICD10_dx | Z51.11 | ENCOUNTER FOR ANTINEOPLASTIC CHEMOTHERAPY |
| Chemo | ICD10_proc | 3E02305 | INTRODUCE OF OTH ANTINEOPLASTIC INTO MUSCLE, PERC APPROACH |
| Chemo | ICD10_proc | 3E0A305 | INTRODUCE OTH ANTINEOPLASTIC IN BONE MARROW, PERC |
| Chemo | ICD10_proc | 3E0F305 | INTRODUCE OTH ANTINEOPLASTIC IN RESP TRACT, PERC |
| Chemo | ICD10_proc | 3E0F705 | INTRODUCE OF OTH ANTINEOPLASTIC INTO RESP TRACT, VIA OPENING |
| Chemo | ICD10_proc | 3E0F805 | INTRODUCTION OF OTHER ANTINEOPLASTIC INTO RESP TRACT, ENDO |
| Chemo | ICD10_proc | 3E0G305 | INTRODUCTION OF OTH ANTINEOPLASTIC INTO UP GI, PERC APPROACH |
| Chemo | ICD10_proc | 3E0G705 | INTRODUCTION OF OTHER ANTINEOPLASTIC INTO UP GI, VIA OPENING |
| Chemo | ICD10_proc | 3E0G805 | INTRODUCTION OF OTHER ANTINEOPLASTIC INTO UPPER GI, ENDO |
| Chemo | ICD10_proc | 3E0H305 | INTRODUCE OF OTH ANTINEOPLASTIC INTO LOW GI, PERC APPROACH |
| Chemo | ICD10_proc | 3E0H705 | INTRODUCTION OF OTH ANTINEOPLASTIC INTO LOW GI, VIA OPENING |
| Chemo | ICD10_proc | 3E0H805 | INTRODUCTION OF OTHER ANTINEOPLASTIC INTO LOWER GI, ENDO |
| Chemo | ICD10_proc | 3E0J305 | INTRODUCE OTH ANTINEOPLASTIC IN BIL/PANC TRACT, PERC |
| Chemo | ICD10_proc | 3E0J705 | INTRODUCE OTH ANTINEOPLASTIC IN BIL/PANC TRACT, VIA OPENING |
| Chemo | ICD10_proc | 3E0J805 | INTRODUCTION OF OTH ANTINEOPLASTIC INTO BIL/PANC TRACT, ENDO |
| Chemo | ICD10_proc | 3E0K305 | INTRODUCE OF OTH ANTINEOPLASTIC INTO GU TRACT, PERC APPROACH |
| Chemo | ICD10_proc | 3E01305 | INTRODUCTION OF OTH ANTINEOPLASTIC INTO SUBCU, PERC APPROACH |
| Chemo | ICD10_proc | 3E0L305 | INTRODUCE OTH ANTINEOPLASTIC IN PLEURAL CAV, PERC |
| Chemo | ICD10_proc | 3E0L705 | INTRODUCE OTH ANTINEOPLASTIC IN PLEURAL CAV, VIA OPENING |
| Chemo | ICD10_proc | 3E0M305 | INTRODUCE OTH ANTINEOPLASTIC IN PERITON CAV, PERC |
| Chemo | ICD10_proc | 3E0M705 | INTRODUCE OTH ANTINEOPLASTIC IN PERITON CAV, VIA OPENING |
| Chemo | ICD10_proc | 3E0Q005 | INTRODUCE OTH ANTINEOPLASTIC IN CRAN CAV/BRAIN, OPEN |
| Chemo | ICD10_proc | 3E0Q305 | INTRODUCE OTH ANTINEOPLASTIC IN CRAN CAV/BRAIN, PERC |
| Chemo | ICD10_proc | 3E0Q705 | INTRODUCE OTH ANTINEOPLASTIC IN CRAN CAV/BRAIN, VIA OPENING |
| Chemo | ICD10_proc | 3E0R305 | INTRODUCE OTH ANTINEOPLASTIC IN SPINAL CANAL, PERC |
| Chemo | ICD10_proc | 3E0S305 | INTRODUCE OTH ANTINEOPLASTIC IN EPIDURAL SPACE, PERC |
| Chemo | ICD10_proc | 3E0V305 | INTRODUCTION OF OTH ANTINEOPLASTIC INTO BONE, PERC APPROACH |
| Chemo | ICD10_proc | 3E0W305 | INTRODUCTION OF OTH ANTINEOPLASTIC INTO LYMPH, PERC APPROACH |
| Chemo | ICD10_proc | 3E00X05 | INTRODUCE OTH ANTINEOPLASTIC IN SKIN/MUCOUS MEM, EXTERN |
| Chemo | ICD10_proc | 3E0M3BZ | INTRODUCTION OF ANESTHETIC INTO PERITON CAV, PERC APPROACH |
| Chemo | ICD10_proc | 3E00XTZ | INTRODUCE DESTR AGENT IN SKIN/MUCOUS MEM, EXTERN |
| Chemo | ICD10_proc | 3E03305 | INTRODUCE OTH ANTINEOPLASTIC IN PERIPH VEIN, PERC |
| Chemo | ICD10_proc | 3E04305 | INTRODUCE OTH ANTINEOPLASTIC IN CENTRAL VEIN, PERC |
| Chemo | ICD10_proc | XW033B3 | INTRODUCE CYTARAB/DAUNORUB IN PERIPH VEIN, PERC, NEW TECH 3 |
| Chemo | ICD10_proc | XW033C3 | INTRODUCE AUTO CAR T CELL IN PERIPH VEIN, PERC, NEW TECH 3 |
| Chemo | ICD10_proc | XW03351 | INTRODUCE BLINATUMOMAB IN PERIPH VEIN, PERC, NEW TECH 1 |
| Chemo | ICD10_proc | XW043B3 | INTRODUCE CYTARAB/DAUNORUB IN CENTRAL VEIN, PERC, NEW TECH 3 |
| Chemo | ICD10_proc | XW043C3 | INTRODUCE AUTO CAR T CELL IN CENTRAL VEIN, PERC, NEW TECH 3 |
| Chemo | ICD10_proc | XW04351 | INTRODUCE BLINATUMOMAB IN CENTRAL VEIN, PERC, NEW TECH 1 |
| immuno | ICD9_dx | V58.12 | Encounter for antineoplastic immunotherapy |
| immuno | ICD10_dx | Z51.12 | Encounter for antineoplastic immunotherapy |
| Chemo | CPT | 96401 | Chemotherapy administration, subcutaneous or intramuscular; non-hormonal anti-neoplastic |
| Chemo | CPT | 96402 | Chemotherapy administration, subcutaneous or intramuscular; hormonal anti-neoplastic |
| Chemo | CPT | 96405 | Chemotherapy administration; intralesional, up to and including 7 lesions |
| Chemo | CPT | 96406 | Chemotherapy administration; intralesional, more than 7 lesions |
| Chemo | CPT | 96409 | Chemotherapy administration; intravenous, push technique, single or initial substance/drug |
| Chemo | CPT | 96411 | Chemotherapy administration; intravenous, push technique, each additional substance/drug (List separately in addition to code for primary procedure) |
| Chemo | CPT | 96413 | Chemotherapy administration, intravenous infusion technique; up to 1 hour, single or initial substance/drug |
| Chemo | CPT | 96415 | Chemotherapy administration, intravenous infusion technique; each additional hour (List separately in addition to code for primary procedure) |
| Chemo | CPT | 96416 | Chemotherapy administration, intravenous infusion technique; initiation of prolonged chemotherapy infusion (more than 8 hours), requiring use of a portable or implantable pump |
| Chemo | CPT | 96417 | Chemotherapy administration, intravenous infusion technique; each additional sequential infusion (different substance/drug), up to 1 hour (List separately in addition to code for primary procedure) |
| Chemo | CPT | 96420 | Chemotherapy administration, intra-arterial; push technique |
| Chemo | CPT | 96422 | Chemotherapy administration, intra-arterial; infusion technique, up to 1 hour |
| Chemo | CPT | 96423 | Chemotherapy administration, intra-arterial; infusion technique, each additional hour (List separately in addition to code for primary procedure) |
| Chemo | CPT | 96425 | Chemotherapy administration, intra-arterial; infusion technique, initiation of prolonged infusion (more than 8 hours), requiring the use of a portable or implantable pump |
| Chemo | CPT | 96440 | Chemotherapy administration into pleural cavity, requiring and including thoracentesis |
| Chemo | CPT | 96446 | Chemotherapy administration into the peritoneal cavity via indwelling port or catheter |
| Chemo | CPT | 96450 | Chemotherapy administration, into CNS (e.g., intrathecal), requiring and including spinal puncture |
| Chemo | CPT | 96521 | Refilling and maintenance of portable pump |
| Chemo | CPT | 96522 | Refilling and maintenance of implantable pump or reservoir for drug delivery, systemic (e.g., intravenous, intra-arterial) |
| Chemo | CPT | 96523 | Irrigation of implanted venous access device for drug delivery systems |
| Chemo | CPT | 96542 | Chemotherapy injection, subarachnoid or intraventricular via subcutaneous reservoir, single or multiple agents |
| Chemo | CPT | 96549 | Unlisted chemotherapy procedure |
| Chemo | CPT | 96567 | Photodynamic therapy by external application of light to destroy premalignant and/or malignant lesions of the skin and adjacent mucosa (eg, lip) by activation of photosensitive drug(s), each phototherapy exposure session |
| Chemo | CPT | 96570 | Photodynamic therapy by endoscopic application of light to ablate abnormal tissue via activation of photosensitive drug(s); first 30 minutes (List separately in addition to code for endoscopy or bronchoscopy procedures of lung and gastrointestinal tract) |
| Chemo | CPT | 96571 | Photodynamic therapy by endoscopic application of light to ablate abnormal tissue via activation of photosensitive drug(s); each additional 15 minutes (List separately in addition to code for endoscopy or bronchoscopy procedures of lung and gastrointestinal tract) |
| Chemo | HCPCS | J7502 | Cyclosporine, oral, 100 mg |
| Chemo | CPT | 90586 | Bacillus Calmette-Guerin vaccine (BCG) for bladder cancer, live, for intravesical use |
| Chemo | HCPCS | C9131 | Injection, ado-trastuzumab emtansine, 1 mg |
| Chemo | HCPCS | C1178 | Injection, busulfan, per 6 mg |
| Chemo | HCPCS | C9262 | Fludarabine phosphate, oral, 1 mg |
| Chemo | HCPCS | C9265 | Injection, romidepsin, 1 mg |
| Chemo | HCPCS | C9287 | Injection, brentuximab vedotin, 1 mg |
| Chemo | HCPCS | C9235 | Injection, panitumumab, 10 mg |
| Chemo | HCPCS | C9284 | Injection, ipilimumab, 1 mg |

Supplemental Table 3. IV codes for systemic therapy drugs.

| **HCPCS code** | | | | | | | | | | |
| --- | --- | --- | --- | --- | --- | --- | --- | --- | --- | --- |
| A9543 | J8602 | J8752 | J8902 | J9052 | J9202 | J9352 | J9502 | J9652 | J9802 | J9952 |
| A9606 | J8603 | J8753 | J8903 | J9053 | J9203 | J9353 | J9503 | J9653 | J9803 | J9953 |
| C9016 | J8604 | J8754 | J8904 | J9054 | J9204 | J9354 | J9504 | J9654 | J9804 | J9954 |
| C9021 | J8605 | J8755 | J8905 | J9055 | J9205 | J9355 | J9505 | J9655 | J9805 | J9955 |
| C9024 | J8606 | J8756 | J8906 | J9056 | J9206 | J9356 | J9506 | J9656 | J9806 | J9956 |
| C9025 | J8607 | J8757 | J8907 | J9057 | J9207 | J9357 | J9507 | J9657 | J9807 | J9957 |
| C9027 | J8608 | J8758 | J8908 | J9058 | J9208 | J9358 | J9508 | J9658 | J9808 | J9958 |
| C9028 | J8609 | J8759 | J8909 | J9059 | J9209 | J9359 | J9509 | J9659 | J9809 | J9959 |
| C9030 | J8610 | J8760 | J8910 | J9060 | J9210 | J9360 | J9510 | J9660 | J9810 | J9960 |
| C9038 | J8611 | J8761 | J8911 | J9061 | J9211 | J9361 | J9511 | J9661 | J9811 | J9961 |
| C9042 | J8612 | J8762 | J8912 | J9062 | J9212 | J9362 | J9512 | J9662 | J9812 | J9962 |
| C9044 | J8613 | J8763 | J8913 | J9063 | J9213 | J9363 | J9513 | J9663 | J9813 | J9963 |
| C9045 | J8614 | J8764 | J8914 | J9064 | J9214 | J9364 | J9514 | J9664 | J9814 | J9964 |
| C9049 | J8615 | J8765 | J8915 | J9065 | J9215 | J9365 | J9515 | J9665 | J9815 | J9965 |
| C9050 | J8616 | J8766 | J8916 | J9066 | J9216 | J9366 | J9516 | J9666 | J9816 | J9966 |
| C9062 | J8617 | J8767 | J8917 | J9067 | J9217 | J9367 | J9517 | J9667 | J9817 | J9967 |
| C9064 | J8618 | J8768 | J8918 | J9068 | J9218 | J9368 | J9518 | J9668 | J9818 | J9968 |
| C9066 | J8619 | J8769 | J8919 | J9069 | J9219 | J9369 | J9519 | J9669 | J9819 | J9969 |
| C9131 | J8620 | J8770 | J8920 | J9070 | J9220 | J9370 | J9520 | J9670 | J9820 | J9970 |
| C9257 | J8621 | J8771 | J8921 | J9071 | J9221 | J9371 | J9521 | J9671 | J9821 | J9971 |
| C9416 | J8622 | J8772 | J8922 | J9072 | J9222 | J9372 | J9522 | J9672 | J9822 | J9972 |
| C9442 | J8623 | J8773 | J8923 | J9073 | J9223 | J9373 | J9523 | J9673 | J9823 | J9973 |
| C9449 | J8624 | J8774 | J8924 | J9074 | J9224 | J9374 | J9524 | J9674 | J9824 | J9974 |
| C9453 | J8625 | J8775 | J8925 | J9075 | J9225 | J9375 | J9525 | J9675 | J9825 | J9975 |
| C9467 | J8626 | J8776 | J8926 | J9076 | J9226 | J9376 | J9526 | J9676 | J9826 | J9976 |
| C9472 | J8627 | J8777 | J8927 | J9077 | J9227 | J9377 | J9527 | J9677 | J9827 | J9977 |
| C9474 | J8628 | J8778 | J8928 | J9078 | J9228 | J9378 | J9528 | J9678 | J9828 | J9978 |
| C9475 | J8629 | J8779 | J8929 | J9079 | J9229 | J9379 | J9529 | J9679 | J9829 | J9979 |
| C9476 | J8630 | J8780 | J8930 | J9080 | J9230 | J9380 | J9530 | J9680 | J9830 | J9980 |
| C9477 | J8631 | J8781 | J8931 | J9081 | J9231 | J9381 | J9531 | J9681 | J9831 | J9981 |
| C9480 | J8632 | J8782 | J8932 | J9082 | J9232 | J9382 | J9532 | J9682 | J9832 | J9982 |
| C9483 | J8633 | J8783 | J8933 | J9083 | J9233 | J9383 | J9533 | J9683 | J9833 | J9983 |
| C9485 | J8634 | J8784 | J8934 | J9084 | J9234 | J9384 | J9534 | J9684 | J9834 | J9984 |
| C9491 | J8635 | J8785 | J8935 | J9085 | J9235 | J9385 | J9535 | J9685 | J9835 | J9985 |
| C9492 | J8636 | J8786 | J8936 | J9086 | J9236 | J9386 | J9536 | J9686 | J9836 | J9986 |
| J0202 | J8637 | J8787 | J8937 | J9087 | J9237 | J9387 | J9537 | J9687 | J9837 | J9987 |
| J0594 | J8638 | J8788 | J8938 | J9088 | J9238 | J9388 | J9538 | J9688 | J9838 | J9988 |
| J0640 | J8639 | J8789 | J8939 | J9089 | J9239 | J9389 | J9539 | J9689 | J9839 | J9989 |
| J1100 | J8640 | J8790 | J8940 | J9090 | J9240 | J9390 | J9540 | J9690 | J9840 | J9990 |
| J1950 | J8641 | J8791 | J8941 | J9091 | J9241 | J9391 | J9541 | J9691 | J9841 | J9991 |
| J2353 | J8642 | J8792 | J8942 | J9092 | J9242 | J9392 | J9542 | J9692 | J9842 | J9992 |
| J2354 | J8643 | J8793 | J8943 | J9093 | J9243 | J9393 | J9543 | J9693 | J9843 | J9993 |
| J2920 | J8644 | J8794 | J8944 | J9094 | J9244 | J9394 | J9544 | J9694 | J9844 | J9994 |
| J2930 | J8645 | J8795 | J8945 | J9095 | J9245 | J9395 | J9545 | J9695 | J9845 | J9995 |
| J3315 | J8646 | J8796 | J8946 | J9096 | J9246 | J9396 | J9546 | J9696 | J9846 | J9996 |
| J3316 | J8647 | J8797 | J8947 | J9097 | J9247 | J9397 | J9547 | J9697 | J9847 | J9997 |
| J7509 | J8648 | J8798 | J8948 | J9098 | J9248 | J9398 | J9548 | J9698 | J9848 | J9998 |
| J7520 | J8649 | J8799 | J8949 | J9099 | J9249 | J9399 | J9549 | J9699 | J9849 | J9999 |
| J7527 | J8650 | J8800 | J8950 | J9100 | J9250 | J9400 | J9550 | J9700 | J9850 | Q2040 |
| J8501 | J8651 | J8801 | J8951 | J9101 | J9251 | J9401 | J9551 | J9701 | J9851 | Q2041 |
| J8502 | J8652 | J8802 | J8952 | J9102 | J9252 | J9402 | J9552 | J9702 | J9852 | Q2042 |
| J8503 | J8653 | J8803 | J8953 | J9103 | J9253 | J9403 | J9553 | J9703 | J9853 | Q2043 |
| J8504 | J8654 | J8804 | J8954 | J9104 | J9254 | J9404 | J9554 | J9704 | J9854 | Q2049 |
| J8505 | J8655 | J8805 | J8955 | J9105 | J9255 | J9405 | J9555 | J9705 | J9855 | Q2050 |
| J8506 | J8656 | J8806 | J8956 | J9106 | J9256 | J9406 | J9556 | J9706 | J9856 | Q5107 |
| J8507 | J8657 | J8807 | J8957 | J9107 | J9257 | J9407 | J9557 | J9707 | J9857 | Q5112 |
| J8508 | J8658 | J8808 | J8958 | J9108 | J9258 | J9408 | J9558 | J9708 | J9858 | Q5113 |
| J8509 | J8659 | J8809 | J8959 | J9109 | J9259 | J9409 | J9559 | J9709 | J9859 | Q5114 |
| J8510 | J8660 | J8810 | J8960 | J9110 | J9260 | J9410 | J9560 | J9710 | J9860 | Q5115 |
| J8511 | J8661 | J8811 | J8961 | J9111 | J9261 | J9411 | J9561 | J9711 | J9861 | Q5116 |
| J8512 | J8662 | J8812 | J8962 | J9112 | J9262 | J9412 | J9562 | J9712 | J9862 | Q5117 |
| J8513 | J8663 | J8813 | J8963 | J9113 | J9263 | J9413 | J9563 | J9713 | J9863 | Q5118 |
| J8514 | J8664 | J8814 | J8964 | J9114 | J9264 | J9414 | J9564 | J9714 | J9864 | Q5119 |
| J8515 | J8665 | J8815 | J8965 | J9115 | J9265 | J9415 | J9565 | J9715 | J9865 | Q5123 |
| J8516 | J8666 | J8816 | J8966 | J9116 | J9266 | J9416 | J9566 | J9716 | J9866 |  |
| J8517 | J8667 | J8817 | J8967 | J9117 | J9267 | J9417 | J9567 | J9717 | J9867 |  |
| J8518 | J8668 | J8818 | J8968 | J9118 | J9268 | J9418 | J9568 | J9718 | J9868 |  |
| J8519 | J8669 | J8819 | J8969 | J9119 | J9269 | J9419 | J9569 | J9719 | J9869 |  |
| J8520 | J8670 | J8820 | J8970 | J9120 | J9270 | J9420 | J9570 | J9720 | J9870 |  |
| J8521 | J8671 | J8821 | J8971 | J9121 | J9271 | J9421 | J9571 | J9721 | J9871 |  |
| J8522 | J8672 | J8822 | J8972 | J9122 | J9272 | J9422 | J9572 | J9722 | J9872 |  |
| J8523 | J8673 | J8823 | J8973 | J9123 | J9273 | J9423 | J9573 | J9723 | J9873 |  |
| J8524 | J8674 | J8824 | J8974 | J9124 | J9274 | J9424 | J9574 | J9724 | J9874 |  |
| J8525 | J8675 | J8825 | J8975 | J9125 | J9275 | J9425 | J9575 | J9725 | J9875 |  |
| J8526 | J8676 | J8826 | J8976 | J9126 | J9276 | J9426 | J9576 | J9726 | J9876 |  |
| J8527 | J8677 | J8827 | J8977 | J9127 | J9277 | J9427 | J9577 | J9727 | J9877 |  |
| J8528 | J8678 | J8828 | J8978 | J9128 | J9278 | J9428 | J9578 | J9728 | J9878 |  |
| J8529 | J8679 | J8829 | J8979 | J9129 | J9279 | J9429 | J9579 | J9729 | J9879 |  |
| J8530 | J8680 | J8830 | J8980 | J9130 | J9280 | J9430 | J9580 | J9730 | J9880 |  |
| J8531 | J8681 | J8831 | J8981 | J9131 | J9281 | J9431 | J9581 | J9731 | J9881 |  |
| J8532 | J8682 | J8832 | J8982 | J9132 | J9282 | J9432 | J9582 | J9732 | J9882 |  |
| J8533 | J8683 | J8833 | J8983 | J9133 | J9283 | J9433 | J9583 | J9733 | J9883 |  |
| J8534 | J8684 | J8834 | J8984 | J9134 | J9284 | J9434 | J9584 | J9734 | J9884 |  |
| J8535 | J8685 | J8835 | J8985 | J9135 | J9285 | J9435 | J9585 | J9735 | J9885 |  |
| J8536 | J8686 | J8836 | J8986 | J9136 | J9286 | J9436 | J9586 | J9736 | J9886 |  |
| J8537 | J8687 | J8837 | J8987 | J9137 | J9287 | J9437 | J9587 | J9737 | J9887 |  |
| J8538 | J8688 | J8838 | J8988 | J9138 | J9288 | J9438 | J9588 | J9738 | J9888 |  |
| J8539 | J8689 | J8839 | J8989 | J9139 | J9289 | J9439 | J9589 | J9739 | J9889 |  |
| J8540 | J8690 | J8840 | J8990 | J9140 | J9290 | J9440 | J9590 | J9740 | J9890 |  |
| J8541 | J8691 | J8841 | J8991 | J9141 | J9291 | J9441 | J9591 | J9741 | J9891 |  |
| J8542 | J8692 | J8842 | J8992 | J9142 | J9292 | J9442 | J9592 | J9742 | J9892 |  |
| J8543 | J8693 | J8843 | J8993 | J9143 | J9293 | J9443 | J9593 | J9743 | J9893 |  |
| J8544 | J8694 | J8844 | J8994 | J9144 | J9294 | J9444 | J9594 | J9744 | J9894 |  |
| J8545 | J8695 | J8845 | J8995 | J9145 | J9295 | J9445 | J9595 | J9745 | J9895 |  |
| J8546 | J8696 | J8846 | J8996 | J9146 | J9296 | J9446 | J9596 | J9746 | J9896 |  |
| J8547 | J8697 | J8847 | J8997 | J9147 | J9297 | J9447 | J9597 | J9747 | J9897 |  |
| J8548 | J8698 | J8848 | J8998 | J9148 | J9298 | J9448 | J9598 | J9748 | J9898 |  |
| J8549 | J8699 | J8849 | J8999 | J9149 | J9299 | J9449 | J9599 | J9749 | J9899 |  |
| J8550 | J8700 | J8850 | J9000 | J9150 | J9300 | J9450 | J9600 | J9750 | J9900 |  |
| J8551 | J8701 | J8851 | J9001 | J9151 | J9301 | J9451 | J9601 | J9751 | J9901 |  |
| J8552 | J8702 | J8852 | J9002 | J9152 | J9302 | J9452 | J9602 | J9752 | J9902 |  |
| J8553 | J8703 | J8853 | J9003 | J9153 | J9303 | J9453 | J9603 | J9753 | J9903 |  |
| J8554 | J8704 | J8854 | J9004 | J9154 | J9304 | J9454 | J9604 | J9754 | J9904 |  |
| J8555 | J8705 | J8855 | J9005 | J9155 | J9305 | J9455 | J9605 | J9755 | J9905 |  |
| J8556 | J8706 | J8856 | J9006 | J9156 | J9306 | J9456 | J9606 | J9756 | J9906 |  |
| J8557 | J8707 | J8857 | J9007 | J9157 | J9307 | J9457 | J9607 | J9757 | J9907 |  |
| J8558 | J8708 | J8858 | J9008 | J9158 | J9308 | J9458 | J9608 | J9758 | J9908 |  |
| J8559 | J8709 | J8859 | J9009 | J9159 | J9309 | J9459 | J9609 | J9759 | J9909 |  |
| J8560 | J8710 | J8860 | J9010 | J9160 | J9310 | J9460 | J9610 | J9760 | J9910 |  |
| J8561 | J8711 | J8861 | J9011 | J9161 | J9311 | J9461 | J9611 | J9761 | J9911 |  |
| J8562 | J8712 | J8862 | J9012 | J9162 | J9312 | J9462 | J9612 | J9762 | J9912 |  |
| J8563 | J8713 | J8863 | J9013 | J9163 | J9313 | J9463 | J9613 | J9763 | J9913 |  |
| J8564 | J8714 | J8864 | J9014 | J9164 | J9314 | J9464 | J9614 | J9764 | J9914 |  |
| J8565 | J8715 | J8865 | J9015 | J9165 | J9315 | J9465 | J9615 | J9765 | J9915 |  |
| J8566 | J8716 | J8866 | J9016 | J9166 | J9316 | J9466 | J9616 | J9766 | J9916 |  |
| J8567 | J8717 | J8867 | J9017 | J9167 | J9317 | J9467 | J9617 | J9767 | J9917 |  |
| J8568 | J8718 | J8868 | J9018 | J9168 | J9318 | J9468 | J9618 | J9768 | J9918 |  |
| J8569 | J8719 | J8869 | J9019 | J9169 | J9319 | J9469 | J9619 | J9769 | J9919 |  |
| J8570 | J8720 | J8870 | J9020 | J9170 | J9320 | J9470 | J9620 | J9770 | J9920 |  |
| J8571 | J8721 | J8871 | J9021 | J9171 | J9321 | J9471 | J9621 | J9771 | J9921 |  |
| J8572 | J8722 | J8872 | J9022 | J9172 | J9322 | J9472 | J9622 | J9772 | J9922 |  |
| J8573 | J8723 | J8873 | J9023 | J9173 | J9323 | J9473 | J9623 | J9773 | J9923 |  |
| J8574 | J8724 | J8874 | J9024 | J9174 | J9324 | J9474 | J9624 | J9774 | J9924 |  |
| J8575 | J8725 | J8875 | J9025 | J9175 | J9325 | J9475 | J9625 | J9775 | J9925 |  |
| J8576 | J8726 | J8876 | J9026 | J9176 | J9326 | J9476 | J9626 | J9776 | J9926 |  |
| J8577 | J8727 | J8877 | J9027 | J9177 | J9327 | J9477 | J9627 | J9777 | J9927 |  |
| J8578 | J8728 | J8878 | J9028 | J9178 | J9328 | J9478 | J9628 | J9778 | J9928 |  |
| J8579 | J8729 | J8879 | J9029 | J9179 | J9329 | J9479 | J9629 | J9779 | J9929 |  |
| J8580 | J8730 | J8880 | J9030 | J9180 | J9330 | J9480 | J9630 | J9780 | J9930 |  |
| J8581 | J8731 | J8881 | J9031 | J9181 | J9331 | J9481 | J9631 | J9781 | J9931 |  |
| J8582 | J8732 | J8882 | J9032 | J9182 | J9332 | J9482 | J9632 | J9782 | J9932 |  |
| J8583 | J8733 | J8883 | J9033 | J9183 | J9333 | J9483 | J9633 | J9783 | J9933 |  |
| J8584 | J8734 | J8884 | J9034 | J9184 | J9334 | J9484 | J9634 | J9784 | J9934 |  |
| J8585 | J8735 | J8885 | J9035 | J9185 | J9335 | J9485 | J9635 | J9785 | J9935 |  |
| J8586 | J8736 | J8886 | J9036 | J9186 | J9336 | J9486 | J9636 | J9786 | J9936 |  |
| J8587 | J8737 | J8887 | J9037 | J9187 | J9337 | J9487 | J9637 | J9787 | J9937 |  |
| J8588 | J8738 | J8888 | J9038 | J9188 | J9338 | J9488 | J9638 | J9788 | J9938 |  |
| J8589 | J8739 | J8889 | J9039 | J9189 | J9339 | J9489 | J9639 | J9789 | J9939 |  |
| J8590 | J8740 | J8890 | J9040 | J9190 | J9340 | J9490 | J9640 | J9790 | J9940 |  |
| J8591 | J8741 | J8891 | J9041 | J9191 | J9341 | J9491 | J9641 | J9791 | J9941 |  |
| J8592 | J8742 | J8892 | J9042 | J9192 | J9342 | J9492 | J9642 | J9792 | J9942 |  |
| J8593 | J8743 | J8893 | J9043 | J9193 | J9343 | J9493 | J9643 | J9793 | J9943 |  |
| J8594 | J8744 | J8894 | J9044 | J9194 | J9344 | J9494 | J9644 | J9794 | J9944 |  |
| J8595 | J8745 | J8895 | J9045 | J9195 | J9345 | J9495 | J9645 | J9795 | J9945 |  |
| J8596 | J8746 | J8896 | J9046 | J9196 | J9346 | J9496 | J9646 | J9796 | J9946 |  |
| J8597 | J8747 | J8897 | J9047 | J9197 | J9347 | J9497 | J9647 | J9797 | J9947 |  |
| J8598 | J8748 | J8898 | J9048 | J9198 | J9348 | J9498 | J9648 | J9798 | J9948 |  |
| J8599 | J8749 | J8899 | J9049 | J9199 | J9349 | J9499 | J9649 | J9799 | J9949 |  |
| J8600 | J8750 | J8900 | J9050 | J9200 | J9350 | J9500 | J9650 | J9800 | J9950 |  |
| J8601 | J8751 | J8901 | J9051 | J9201 | J9351 | J9501 | J9651 | J9801 | J9951 |  |

Supplemental Table 4. Claims Codes for Radiation.

| Code Type | Code | Description |
| --- | --- | --- |
| HCPCS/CPT | 0073T | DELIVERY COMP IMRT |
| HCPCS/CPT | 0182T | HDR ELECT BRACHYTHERAPY |
| HCPCS/CPT | 0190T | PLACE INTRAOC RADIATION SRC |
| HCPCS/CPT | 20555 | PLACE NDL MUSC/TIS FOR RT |
| HCPCS/CPT | 55920 | PLACE NEEDLES PELVIC FOR RT |
| HCPCS/CPT | 57155 | INSERT UTERI TANDEM/OVOIDS |
| HCPCS/CPT | 58346 | INSERT HEYMAN UTERI CAPSULE |
| HCPCS/CPT | 77371 | SRS MULTISOURCE |
| HCPCS/CPT | 77372 | SRS LINEAR BASED |
| HCPCS/CPT | 77373 | SBRT DELIVERY |
| HCPCS/CPT | 77399 | EXTERNAL RADIATION DOSIMETRY |
| HCPCS/CPT | 77401 | RADIATION TREATMENT DELIVERY |
| HCPCS/CPT | 77402 | RADIATION TREATMENT DELIVERY |
| HCPCS/CPT | 77403 | RADIATION TREATMENT DELIVERY |
| HCPCS/CPT | 77404 | RADIATION TREATMENT DELIVERY |
| HCPCS/CPT | 77406 | RADIATION TREATMENT DELIVERY |
| HCPCS/CPT | 77407 | RADIATION TREATMENT DELIVERY |
| HCPCS/CPT | 77408 | RADIATION TREATMENT DELIVERY |
| HCPCS/CPT | 77409 | RADIATION TREATMENT DELIVERY |
| HCPCS/CPT | 77411 | RADIATION TREATMENT DELIVERY |
| HCPCS/CPT | 77412 | RADIATION TREATMENT DELIVERY |
| HCPCS/CPT | 77413 | RADIATION TREATMENT DELIVERY |
| HCPCS/CPT | 77414 | RADIATION TREATMENT DELIVERY |
| HCPCS/CPT | 77416 | RADIATION TREATMENT DELIVERY |
| HCPCS/CPT | 77417 | RADIOLOGY PORT FILM(S) |
| HCPCS/CPT | 77418 | NULL |
| HCPCS/CPT | 77421 | STEREOSCOPIC X-RAY GUIDANCE |
| HCPCS/CPT | 77422 | NEUTRON BEAM TX SIMPLE |
| HCPCS/CPT | 77423 | NEUTRON BEAM TX COMPLEX |
| HCPCS/CPT | 77427 | RADIATION TX MANAGEMENT X5 |
| HCPCS/CPT | 77431 | RADIATION THERAPY MANAGEMENT |
| HCPCS/CPT | 77432 | STEREOTACTIC RADIATION TRMT |
| HCPCS/CPT | 77435 | SBRT MANAGEMENT |
| HCPCS/CPT | 77470 | SPECIAL RADIATION TREATMENT |
| HCPCS/CPT | 77499 | RADIATION THERAPY MANAGEMENT |
| HCPCS/CPT | 77520 | PROTON TRMT SIMPLE W/O COMP |
| HCPCS/CPT | 77522 | PROTON TRMT SIMPLE W/COMP |
| HCPCS/CPT | 77523 | PROTON TRMT INTERMEDIATE |
| HCPCS/CPT | 77525 | PROTON TREATMENT COMPLEX |
| HCPCS/CPT | 77600 | HYPERTHERMIA TREATMENT |
| HCPCS/CPT | 77605 | HYPERTHERMIA TREATMENT |
| HCPCS/CPT | 77610 | NULL |
| HCPCS/CPT | 77615 | HYPERTHERMIA TREATMENT |
| HCPCS/CPT | 77620 | HYPERTHERMIA TREATMENT |
| HCPCS/CPT | 77750 | INFUSE RADIOACTIVE MATERIALS |
| HCPCS/CPT | 77761 | APPLY INTRCAV RADIAT SIMPLE |
| HCPCS/CPT | 77762 | APPLY INTRCAV RADIAT INTERM |
| HCPCS/CPT | 77763 | APPLY INTRCAV RADIAT COMPL |
| HCPCS/CPT | 77776 | APPLY INTERSTIT RADIAT SIMPL |
| HCPCS/CPT | 77777 | APPLY INTERSTIT RADIAT INTER |
| HCPCS/CPT | 77778 | APPLY INTERSTIT RADIAT COMPL |
| HCPCS/CPT | 77785 | HDR BRACHYTX 1 CHANNEL |
| HCPCS/CPT | 77786 | HDR BRACHYTX 2-12 CHANNEL |
| HCPCS/CPT | 77787 | HDR BRACHYTX OVER 12 CHAN |
| HCPCS/CPT | 77789 | APPLY SURFACE RADIATION |
| HCPCS/CPT | 77790 | RADIATION HANDLING |
| HCPCS/CPT | 77799 | RADIUM/RADIOISOTOPE THERAPY |
| HCPCS/CPT | 79200 | NUCLEAR RX INTRACAV ADMIN |
| HCPCS/CPT | 79300 | NUCLR RX INTERSTIT COLLOID |
| HCPCS/CPT | 79440 | NUCLEAR RX INTRA-ARTICULAR |
| HCPCS/CPT | 79999 | NUCLEAR MEDICINE THERAPY |
| HCPCS/CPT | 92974 | CATH PLACE CARDIO BRACHYTX |
| HCPCS/CPT | C9725 | PLACE ENDORECTAL APP |
| HCPCS/CPT | C9726 | RXT BREAST APPL PLACE/REMOV |
| HCPCS/CPT | G0173 | LINEAR ACC STEREO RADSUR COM |
| HCPCS/CPT | G0251 | LINEAR ACC BASED STERO RADIO |
| HCPCS/CPT | G0339 | ROBOT LIN-RADSURG COM, FIRST |
| HCPCS/CPT | G0340 | ROBT LIN-RADSURG FRACTX 2-5 |
| HCPCS/CPT | S8049 | INTRAOPERATIVE RADIATION THE |
| HCPCS/CPT | 55859 | PERCUT/NEEDLE INSERT, PROS |
| HCPCS/CPT | 77781 | HIGH INTENSITY BRACHYTHERAPY |
| HCPCS/CPT | 77782 | HIGH INTENSITY BRACHYTHERAPY |
| HCPCS/CPT | 77783 | HIGH INTENSITY BRACHYTHERAPY |
| HCPCS/CPT | 77784 | HIGH INTENSITY BRACHYTHERAPY |
| HCPCS/CPT | G0242 | MULTISOURCE PHOTON STER PLAN |
| HCPCS/CPT | G0243 | MULTISOUR PHOTON STERO TREAT |
| HCPCS/CPT | 0082T | STEREOTACTIC RAD DELIVERY |
| HCPCS/CPT | 0083T | STEREOTACTIC RAD TX MNGMT |
| HCPCS/CPT | G0338 | LINEAR ACCELERATOR STERO PLN |
| HCPCS/CPT | 32553 | INS MARK THOR FOR RT PERQ |
| HCPCS/CPT | 49411 | INS MARK ABD/PEL FOR RT PERQ |
| HCPCS/CPT | 49412 | INS DEVICE FOR RT GUIDE OPEN |
| HCPCS/CPT | 57156 | INS VAG BRACHYTX DEVICE |
| HCPCS/CPT | 77385 | NTSTY MODUL RAD TX DLVR SMPL |
| HCPCS/CPT | 77386 | NTSTY MODUL RAD TX DLVR CPLX |
| HCPCS/CPT | 77387 | GUIDANCE FOR RADIAJ TX DLVR |
| HCPCS/CPT | 77424 | IO RAD TX DELIVERY BY X-RAY |
| HCPCS/CPT | 77425 | IO RAD TX DELIVER BY ELCTRNS |
| HCPCS/CPT | 77469 | IO RADIATION TX MANAGEMENT |
| HCPCS/CPT | G0458 | LDR PROSTATE BRACHY COMP RAT |
| HCPCS/CPT | G6001 | ECHO GUIDANCE RADIOTHERAPY |
| HCPCS/CPT | G6003 | RADIATION TREATMENT DELIVERY |
| HCPCS/CPT | G6004 | RADIATION TREATMENT DELIVERY |
| HCPCS/CPT | G6005 | RADIATION TREATMENT DELIVERY |
| HCPCS/CPT | G6006 | RADIATION TREATMENT DELIVERY |
| HCPCS/CPT | G6007 | RADIATION TREATMENT DELIVERY |
| HCPCS/CPT | G6008 | RADIATION TREATMENT DELIVERY |
| HCPCS/CPT | G6009 | RADIATION TREATMENT DELIVERY |
| HCPCS/CPT | G6010 | RADIATION TREATMENT DELIVERY |
| HCPCS/CPT | G6011 | RADIATION TREATMENT DELIVERY |
| HCPCS/CPT | G6012 | RADIATION TREATMENT DELIVERY |
| HCPCS/CPT | G6013 | RADIATION TREATMENT DELIVERY |
| HCPCS/CPT | G6014 | RADIATION TREATMENT DELIVERY |
| HCPCS/CPT | G6015 | RADIATION TX DELIVERY IMRT |
| HCPCS/CPT | G6016 | DELIVERY COMP IMRT |
| HCPCS/CPT | 32701 | THORAX STEREO RAD TARGETW/TX |
| HCPCS/CPT | 77290 | Therapeutic radiology simulation-aided field setting; complex |
| HCPCS/CPT | 77300 | Basic radiation dosimetry calculation, central axis depth dose calculation, TDF, NSD, gap calculation, off axis factor, tissue inhomogeneity factors, calculation of non-ionizing radiation surface and depth dose, as required during course of treatment, only when prescribed by the treating physician |
| HCPCS/CPT | 77301 | Intensity modulated radiotherapy plan, including dose-volume histograms for target and critical structure partial tolerance specifications |
| HCPCS/CPT | 77334 | Treatment devices, design and construction; complex (irregular blocks, special shields, compensators, wedges, molds or casts) |
| HCPCS/CPT | 77336 | Continuing medical physics consultation, including assessment of treatment parameters, quality assurance of dose delivery, and review of patient treatment documentation in support of the radiation oncologist, reported per week of therapy |
| HCPCS/CPT | 77338 | Multi-leaf collimator (MLC) device(s) for intensity modulated radiation therapy (IMRT), design and construction per IMRT plan |
| HCPCS/CPT | G0174 | IMRT |
| HCPCS/CPT | 0197T | Intra-fraction localization and tracking of target or patient motion during delivery of radiation therapy |
| HCPCS/CPT | 77261 | Radiation Oncology Treatment |
| HCPCS/CPT | 77262 | Radiation Oncology Treatment |
| HCPCS/CPT | 77263 | Radiation Oncology Treatment |
| HCPCS/CPT | 77264 | Radiation Oncology Treatment |
| HCPCS/CPT | 77265 | Radiation Oncology Treatment |
| HCPCS/CPT | 77266 | Radiation Oncology Treatment |
| HCPCS/CPT | 77267 | Radiation Oncology Treatment |
| HCPCS/CPT | 77268 | Radiation Oncology Treatment |
| HCPCS/CPT | 77269 | Radiation Oncology Treatment |
| HCPCS/CPT | 77270 | Radiation Oncology Treatment |
| HCPCS/CPT | 77271 | Radiation Oncology Treatment |
| HCPCS/CPT | 77272 | Radiation Oncology Treatment |
| HCPCS/CPT | 77273 | Radiation Oncology Treatment |
| HCPCS/CPT | 77274 | Radiation Oncology Treatment |
| HCPCS/CPT | 77275 | Radiation Oncology Treatment |
| HCPCS/CPT | 77276 | Radiation Oncology Treatment |
| HCPCS/CPT | 77277 | Radiation Oncology Treatment |
| HCPCS/CPT | 77278 | Radiation Oncology Treatment |
| HCPCS/CPT | 77279 | Radiation Oncology Treatment |
| HCPCS/CPT | 77280 | Radiation Oncology Treatment |
| HCPCS/CPT | 77281 | Radiation Oncology Treatment |
| HCPCS/CPT | 77282 | Radiation Oncology Treatment |
| HCPCS/CPT | 77283 | Radiation Oncology Treatment |
| HCPCS/CPT | 77284 | Radiation Oncology Treatment |
| HCPCS/CPT | 77285 | Radiation Oncology Treatment |
| HCPCS/CPT | 77286 | Radiation Oncology Treatment |
| HCPCS/CPT | 77287 | Radiation Oncology Treatment |
| HCPCS/CPT | 77288 | Radiation Oncology Treatment |
| HCPCS/CPT | 77289 | Radiation Oncology Treatment |
| HCPCS/CPT | 77291 | Radiation Oncology Treatment |
| HCPCS/CPT | 77292 | Radiation Oncology Treatment |
| HCPCS/CPT | 77293 | Radiation Oncology Treatment |
| HCPCS/CPT | 77294 | Radiation Oncology Treatment |
| HCPCS/CPT | 77295 | Radiation Oncology Treatment |
| HCPCS/CPT | 77296 | Radiation Oncology Treatment |
| HCPCS/CPT | 77297 | Radiation Oncology Treatment |
| HCPCS/CPT | 77298 | Radiation Oncology Treatment |
| HCPCS/CPT | 77299 | Radiation Oncology Treatment |
| HCPCS/CPT | 77302 | Radiation Oncology Treatment |
| HCPCS/CPT | 77303 | Radiation Oncology Treatment |
| HCPCS/CPT | 77304 | Radiation Oncology Treatment |
| HCPCS/CPT | 77305 | Radiation Oncology Treatment |
| HCPCS/CPT | 77306 | Radiation Oncology Treatment |
| HCPCS/CPT | 77307 | Radiation Oncology Treatment |
| HCPCS/CPT | 77308 | Radiation Oncology Treatment |
| HCPCS/CPT | 77309 | Radiation Oncology Treatment |
| HCPCS/CPT | 77310 | Radiation Oncology Treatment |
| HCPCS/CPT | 77311 | Radiation Oncology Treatment |
| HCPCS/CPT | 77312 | Radiation Oncology Treatment |
| HCPCS/CPT | 77313 | Radiation Oncology Treatment |
| HCPCS/CPT | 77314 | Radiation Oncology Treatment |
| HCPCS/CPT | 77315 | Radiation Oncology Treatment |
| HCPCS/CPT | 77316 | Radiation Oncology Treatment |
| HCPCS/CPT | 77317 | Radiation Oncology Treatment |
| HCPCS/CPT | 77318 | Radiation Oncology Treatment |
| HCPCS/CPT | 77319 | Radiation Oncology Treatment |
| HCPCS/CPT | 77320 | Radiation Oncology Treatment |
| HCPCS/CPT | 77321 | Radiation Oncology Treatment |
| HCPCS/CPT | 77322 | Radiation Oncology Treatment |
| HCPCS/CPT | 77323 | Radiation Oncology Treatment |
| HCPCS/CPT | 77324 | Radiation Oncology Treatment |
| HCPCS/CPT | 77325 | Radiation Oncology Treatment |
| HCPCS/CPT | 77326 | Radiation Oncology Treatment |
| HCPCS/CPT | 77327 | Radiation Oncology Treatment |
| HCPCS/CPT | 77328 | Radiation Oncology Treatment |
| HCPCS/CPT | 77329 | Radiation Oncology Treatment |
| HCPCS/CPT | 77330 | Radiation Oncology Treatment |
| HCPCS/CPT | 77331 | Radiation Oncology Treatment |
| HCPCS/CPT | 77332 | Radiation Oncology Treatment |
| HCPCS/CPT | 77333 | Radiation Oncology Treatment |
| HCPCS/CPT | 77335 | Radiation Oncology Treatment |
| HCPCS/CPT | 77337 | Radiation Oncology Treatment |
| HCPCS/CPT | 77339 | Radiation Oncology Treatment |
| HCPCS/CPT | 77340 | Radiation Oncology Treatment |
| HCPCS/CPT | 77341 | Radiation Oncology Treatment |
| HCPCS/CPT | 77342 | Radiation Oncology Treatment |
| HCPCS/CPT | 77343 | Radiation Oncology Treatment |
| HCPCS/CPT | 77344 | Radiation Oncology Treatment |
| HCPCS/CPT | 77345 | Radiation Oncology Treatment |
| HCPCS/CPT | 77346 | Radiation Oncology Treatment |
| HCPCS/CPT | 77347 | Radiation Oncology Treatment |
| HCPCS/CPT | 77348 | Radiation Oncology Treatment |
| HCPCS/CPT | 77349 | Radiation Oncology Treatment |
| HCPCS/CPT | 77350 | Radiation Oncology Treatment |
| HCPCS/CPT | 77351 | Radiation Oncology Treatment |
| HCPCS/CPT | 77352 | Radiation Oncology Treatment |
| HCPCS/CPT | 77353 | Radiation Oncology Treatment |
| HCPCS/CPT | 77354 | Radiation Oncology Treatment |
| HCPCS/CPT | 77355 | Radiation Oncology Treatment |
| HCPCS/CPT | 77356 | Radiation Oncology Treatment |
| HCPCS/CPT | 77357 | Radiation Oncology Treatment |
| HCPCS/CPT | 77358 | Radiation Oncology Treatment |
| HCPCS/CPT | 77359 | Radiation Oncology Treatment |
| HCPCS/CPT | 77360 | Radiation Oncology Treatment |
| HCPCS/CPT | 77361 | Radiation Oncology Treatment |
| HCPCS/CPT | 77362 | Radiation Oncology Treatment |
| HCPCS/CPT | 77363 | Radiation Oncology Treatment |
| HCPCS/CPT | 77364 | Radiation Oncology Treatment |
| HCPCS/CPT | 77365 | Radiation Oncology Treatment |
| HCPCS/CPT | 77366 | Radiation Oncology Treatment |
| HCPCS/CPT | 77367 | Radiation Oncology Treatment |
| HCPCS/CPT | 77368 | Radiation Oncology Treatment |
| HCPCS/CPT | 77369 | Radiation Oncology Treatment |
| HCPCS/CPT | 77370 | Radiation Oncology Treatment |
| HCPCS/CPT | 77374 | Radiation Oncology Treatment |
| HCPCS/CPT | 77375 | Radiation Oncology Treatment |
| HCPCS/CPT | 77376 | Radiation Oncology Treatment |
| HCPCS/CPT | 77377 | Radiation Oncology Treatment |
| HCPCS/CPT | 77378 | Radiation Oncology Treatment |
| HCPCS/CPT | 77379 | Radiation Oncology Treatment |
| HCPCS/CPT | 77380 | Radiation Oncology Treatment |
| HCPCS/CPT | 77381 | Radiation Oncology Treatment |
| HCPCS/CPT | 77382 | Radiation Oncology Treatment |
| HCPCS/CPT | 77383 | Radiation Oncology Treatment |
| HCPCS/CPT | 77384 | Radiation Oncology Treatment |
| HCPCS/CPT | 77388 | Radiation Oncology Treatment |
| HCPCS/CPT | 77389 | Radiation Oncology Treatment |
| HCPCS/CPT | 77390 | Radiation Oncology Treatment |
| HCPCS/CPT | 77391 | Radiation Oncology Treatment |
| HCPCS/CPT | 77392 | Radiation Oncology Treatment |
| HCPCS/CPT | 77393 | Radiation Oncology Treatment |
| HCPCS/CPT | 77394 | Radiation Oncology Treatment |
| HCPCS/CPT | 77395 | Radiation Oncology Treatment |
| HCPCS/CPT | 77396 | Radiation Oncology Treatment |
| HCPCS/CPT | 77397 | Radiation Oncology Treatment |
| HCPCS/CPT | 77398 | Radiation Oncology Treatment |
| ICD9_proc | 92.21 | Superficial radiation |
| ICD9_proc | 92.22 | Orthovoltage radiation |
| ICD9_proc | 92.23 | Radioisotopic teleradiotherapy |
| ICD9_proc | 92.24 | Teleradiotherapy using photons |
| ICD9_proc | 92.25 | Teleradiotherapy using electrons |
| ICD9_proc | 92.26 | Teleradiotherapy of other particulate radiation |
| ICD9_proc | 92.27 | Implantation or insertion of radioactive elements |
| ICD9_proc | 92.28 | Injection or instillation of radioisotopes |
| ICD9_proc | 92.29 | Other radiotherapeutic procedure |
| ICD9_proc | 92.41 | Intra-operative electron radiation therapy |
| ICD9_dx | V58.0 | Radiotherapy |
| ICD10_proc | DBY07ZZ | CONTACT RADIATION OF TRACHEA |
| ICD10_proc | DBY17ZZ | CONTACT RADIATION OF BRONCHUS |
| ICD10_proc | DBY27ZZ | CONTACT RADIATION OF LUNG |
| ICD10_proc | DBY57ZZ | CONTACT RADIATION OF PLEURA |
| ICD10_proc | DBY67ZZ | CONTACT RADIATION OF MEDIASTINUM |
| ICD10_proc | DBY77ZZ | CONTACT RADIATION OF CHEST WALL |
| ICD10_proc | DBY87ZZ | CONTACT RADIATION OF DIAPHRAGM |
| ICD10_proc | DDY07ZZ | CONTACT RADIATION OF ESOPHAGUS |
| ICD10_proc | DDY17ZZ | CONTACT RADIATION OF STOMACH |
| ICD10_proc | DDY27ZZ | CONTACT RADIATION OF DUODENUM |
| ICD10_proc | DDY37ZZ | CONTACT RADIATION OF JEJUNUM |
| ICD10_proc | DDY47ZZ | CONTACT RADIATION OF ILEUM |
| ICD10_proc | DDY57ZZ | CONTACT RADIATION OF COLON |
| ICD10_proc | DDY77ZZ | CONTACT RADIATION OF RECTUM |
| ICD10_proc | DFY07ZZ | CONTACT RADIATION OF LIVER |
| ICD10_proc | DFY17ZZ | CONTACT RADIATION OF GALLBLADDER |
| ICD10_proc | DFY27ZZ | CONTACT RADIATION OF BILE DUCTS |
| ICD10_proc | DFY37ZZ | CONTACT RADIATION OF PANCREAS |
| ICD10_proc | DGY07ZZ | CONTACT RADIATION OF PITUITARY GLAND |
| ICD10_proc | DGY17ZZ | CONTACT RADIATION OF PINEAL BODY |
| ICD10_proc | DGY27ZZ | CONTACT RADIATION OF ADRENAL GLANDS |
| ICD10_proc | DGY47ZZ | CONTACT RADIATION OF PARATHYROID GLANDS |
| ICD10_proc | DGY57ZZ | CONTACT RADIATION OF THYROID |
| ICD10_proc | DHYB7ZZ | CONTACT RADIATION OF LEG SKIN |
| ICD10_proc | DHY27ZZ | CONTACT RADIATION OF FACE SKIN |
| ICD10_proc | DHY37ZZ | CONTACT RADIATION OF NECK SKIN |
| ICD10_proc | DHY47ZZ | CONTACT RADIATION OF ARM SKIN |
| ICD10_proc | DHY67ZZ | CONTACT RADIATION OF CHEST SKIN |
| ICD10_proc | DHY77ZZ | CONTACT RADIATION OF BACK SKIN |
| ICD10_proc | DHY87ZZ | CONTACT RADIATION OF ABDOMEN SKIN |
| ICD10_proc | DHY97ZZ | CONTACT RADIATION OF BUTTOCK SKIN |
| ICD10_proc | DMY07ZZ | CONTACT RADIATION OF LEFT BREAST |
| ICD10_proc | DMY17ZZ | CONTACT RADIATION OF RIGHT BREAST |
| ICD10_proc | DPYB7ZZ | CONTACT RADIATION OF TIBIA/FIBULA |
| ICD10_proc | DPYC7ZZ | CONTACT RADIATION OF OTHER BONE |
| ICD10_proc | DPY07ZZ | CONTACT RADIATION OF SKULL |
| ICD10_proc | DPY27ZZ | CONTACT RADIATION OF MAXILLA |
| ICD10_proc | DPY37ZZ | CONTACT RADIATION OF MANDIBLE |
| ICD10_proc | DPY47ZZ | CONTACT RADIATION OF STERNUM |
| ICD10_proc | DPY57ZZ | CONTACT RADIATION OF RIB(S) |
| ICD10_proc | DPY67ZZ | CONTACT RADIATION OF HUMERUS |
| ICD10_proc | DPY77ZZ | CONTACT RADIATION OF RADIUS/ULNA |
| ICD10_proc | DPY87ZZ | CONTACT RADIATION OF PELVIC BONES |
| ICD10_proc | DPY97ZZ | CONTACT RADIATION OF FEMUR |
| ICD10_proc | DTY07ZZ | CONTACT RADIATION OF KIDNEY |
| ICD10_proc | DTY17ZZ | CONTACT RADIATION OF URETER |
| ICD10_proc | DTY27ZZ | CONTACT RADIATION OF BLADDER |
| ICD10_proc | DTY37ZZ | CONTACT RADIATION OF URETHRA |
| ICD10_proc | DUY07ZZ | CONTACT RADIATION OF OVARY |
| ICD10_proc | DUY17ZZ | CONTACT RADIATION OF CERVIX |
| ICD10_proc | DUY27ZZ | CONTACT RADIATION OF UTERUS |
| ICD10_proc | DVY07ZZ | CONTACT RADIATION OF PROSTATE |
| ICD10_proc | DVY17ZZ | CONTACT RADIATION OF TESTIS |
| ICD10_proc | DWY17ZZ | CONTACT RADIATION OF HEAD AND NECK |
| ICD10_proc | DWY27ZZ | CONTACT RADIATION OF CHEST |
| ICD10_proc | DWY37ZZ | CONTACT RADIATION OF ABDOMEN |
| ICD10_proc | DWY47ZZ | CONTACT RADIATION OF HEMIBODY |
| ICD10_proc | DWY57ZZ | CONTACT RADIATION OF WHOLE BODY |
| ICD10_proc | DWY67ZZ | CONTACT RADIATION OF PELVIC REGION |
| ICD10_proc | D0Y07ZZ | CONTACT RADIATION OF BRAIN |
| ICD10_proc | D0Y17ZZ | CONTACT RADIATION OF BRAIN STEM |
| ICD10_proc | D0Y67ZZ | CONTACT RADIATION OF SPINAL CORD |
| ICD10_proc | D0Y77ZZ | CONTACT RADIATION OF PERIPHERAL NERVE |
| ICD10_proc | D8Y07ZZ | CONTACT RADIATION OF EYE |
| ICD10_proc | D9YB7ZZ | CONTACT RADIATION OF LARYNX |
| ICD10_proc | D9YD7ZZ | CONTACT RADIATION OF NASOPHARYNX |
| ICD10_proc | D9YF7ZZ | CONTACT RADIATION OF OROPHARYNX |
| ICD10_proc | D9Y07ZZ | CONTACT RADIATION OF EAR |
| ICD10_proc | D9Y17ZZ | CONTACT RADIATION OF NOSE |
| ICD10_proc | D9Y37ZZ | CONTACT RADIATION OF HYPOPHARYNX |
| ICD10_proc | D9Y47ZZ | CONTACT RADIATION OF MOUTH |
| ICD10_proc | D9Y57ZZ | CONTACT RADIATION OF TONGUE |
| ICD10_proc | D9Y67ZZ | CONTACT RADIATION OF SALIVARY GLANDS |
| ICD10_proc | D9Y77ZZ | CONTACT RADIATION OF SINUSES |
| ICD10_proc | D9Y87ZZ | CONTACT RADIATION OF HARD PALATE |
| ICD10_proc | D9Y97ZZ | CONTACT RADIATION OF SOFT PALATE |
| ICD10_proc | DB000ZZ | BEAM RADIATION OF TRACHEA USING PHOTONS <1 MEV |
| ICD10_proc | DB010ZZ | BEAM RADIATION OF BRONCHUS USING PHOTONS <1 MEV |
| ICD10_proc | DB020ZZ | BEAM RADIATION OF LUNG USING PHOTONS <1 MEV |
| ICD10_proc | DB050ZZ | BEAM RADIATION OF PLEURA USING PHOTONS <1 MEV |
| ICD10_proc | DB060ZZ | BEAM RADIATION OF MEDIASTINUM USING PHOTONS <1 MEV |
| ICD10_proc | DB070ZZ | BEAM RADIATION OF CHEST WALL USING PHOTONS <1 MEV |
| ICD10_proc | DB080ZZ | BEAM RADIATION OF DIAPHRAGM USING PHOTONS <1 MEV |
| ICD10_proc | DD030ZZ | BEAM RADIATION OF JEJUNUM USING PHOTONS <1 MEV |
| ICD10_proc | DD040ZZ | BEAM RADIATION OF ILEUM USING PHOTONS <1 MEV |
| ICD10_proc | DD050ZZ | BEAM RADIATION OF COLON USING PHOTONS <1 MEV |
| ICD10_proc | DD070ZZ | BEAM RADIATION OF RECTUM USING PHOTONS <1 MEV |
| ICD10_proc | DF000ZZ | BEAM RADIATION OF LIVER USING PHOTONS <1 MEV |
| ICD10_proc | DF010ZZ | BEAM RADIATION OF GALLBLADDER USING PHOTONS <1 MEV |
| ICD10_proc | DF020ZZ | BEAM RADIATION OF BILE DUCTS USING PHOTONS <1 MEV |
| ICD10_proc | DF030ZZ | BEAM RADIATION OF PANCREAS USING PHOTONS <1 MEV |
| ICD10_proc | DG000ZZ | BEAM RADIATION OF PITUITARY GLAND USING PHOTONS <1 MEV |
| ICD10_proc | DG010ZZ | BEAM RADIATION OF PINEAL BODY USING PHOTONS <1 MEV |
| ICD10_proc | DG020ZZ | BEAM RADIATION OF ADRENAL GLANDS USING PHOTONS <1 MEV |
| ICD10_proc | DG040ZZ | BEAM RADIATION OF PARATHYROID GLANDS USING PHOTONS <1 MEV |
| ICD10_proc | DG050ZZ | BEAM RADIATION OF THYROID USING PHOTONS <1 MEV |
| ICD10_proc | DH0B0ZZ | BEAM RADIATION OF LEG SKIN USING PHOTONS <1 MEV |
| ICD10_proc | DH020ZZ | BEAM RADIATION OF FACE SKIN USING PHOTONS <1 MEV |
| ICD10_proc | DH030ZZ | BEAM RADIATION OF NECK SKIN USING PHOTONS <1 MEV |
| ICD10_proc | DH040ZZ | BEAM RADIATION OF ARM SKIN USING PHOTONS <1 MEV |
| ICD10_proc | DH060ZZ | BEAM RADIATION OF CHEST SKIN USING PHOTONS <1 MEV |
| ICD10_proc | DH070ZZ | BEAM RADIATION OF BACK SKIN USING PHOTONS <1 MEV |
| ICD10_proc | DH080ZZ | BEAM RADIATION OF ABDOMEN SKIN USING PHOTONS <1 MEV |
| ICD10_proc | DH090ZZ | BEAM RADIATION OF BUTTOCK SKIN USING PHOTONS <1 MEV |
| ICD10_proc | DM000ZZ | BEAM RADIATION OF LEFT BREAST USING PHOTONS <1 MEV |
| ICD10_proc | DP020ZZ | BEAM RADIATION OF MAXILLA USING PHOTONS <1 MEV |
| ICD10_proc | DP030ZZ | BEAM RADIATION OF MANDIBLE USING PHOTONS <1 MEV |
| ICD10_proc | DP040ZZ | BEAM RADIATION OF STERNUM USING PHOTONS <1 MEV |
| ICD10_proc | DP060ZZ | BEAM RADIATION OF HUMERUS USING PHOTONS <1 MEV |
| ICD10_proc | DP070ZZ | BEAM RADIATION OF RADIUS/ULNA USING PHOTONS <1 MEV |
| ICD10_proc | D0070ZZ | BEAM RADIATION OF PERIPHERAL NERVE USING PHOTONS <1 MEV |
| ICD10_proc | D7000ZZ | BEAM RADIATION OF BONE MARROW USING PHOTONS <1 MEV |
| ICD10_proc | D7010ZZ | BEAM RADIATION OF THYMUS USING PHOTONS <1 MEV |
| ICD10_proc | D7020ZZ | BEAM RADIATION OF SPLEEN USING PHOTONS <1 MEV |
| ICD10_proc | D7030ZZ | BEAM RADIATION OF NECK LYMPHATICS USING PHOTONS <1 MEV |
| ICD10_proc | D7040ZZ | BEAM RADIATION OF AXILLARY LYMPHATICS USING PHOTONS <1 MEV |
| ICD10_proc | D7050ZZ | BEAM RADIATION OF THORAX LYMPHATICS USING PHOTONS <1 MEV |
| ICD10_proc | D7060ZZ | BEAM RADIATION OF ABDOMEN LYMPHATICS USING PHOTONS <1 MEV |
| ICD10_proc | D7070ZZ | BEAM RADIATION OF PELVIS LYMPHATICS USING PHOTONS <1 MEV |
| ICD10_proc | D7080ZZ | BEAM RADIATION OF INGUINAL LYMPHATICS USING PHOTONS <1 MEV |
| ICD10_proc | D8000ZZ | BEAM RADIATION OF EYE USING PHOTONS <1 MEV |
| ICD10_proc | D90B0ZZ | BEAM RADIATION OF LARYNX USING PHOTONS <1 MEV |
| ICD10_proc | D90D0ZZ | BEAM RADIATION OF NASOPHARYNX USING PHOTONS <1 MEV |
| ICD10_proc | D90F0ZZ | BEAM RADIATION OF OROPHARYNX USING PHOTONS <1 MEV |
| ICD10_proc | D9000ZZ | BEAM RADIATION OF EAR USING PHOTONS <1 MEV |
| ICD10_proc | D9010ZZ | BEAM RADIATION OF NOSE USING PHOTONS <1 MEV |
| ICD10_proc | D9030ZZ | BEAM RADIATION OF HYPOPHARYNX USING PHOTONS <1 MEV |
| ICD10_proc | D9040ZZ | BEAM RADIATION OF MOUTH USING PHOTONS <1 MEV |
| ICD10_proc | D9050ZZ | BEAM RADIATION OF TONGUE USING PHOTONS <1 MEV |
| ICD10_proc | D9060ZZ | BEAM RADIATION OF SALIVARY GLANDS USING PHOTONS <1 MEV |
| ICD10_proc | D9070ZZ | BEAM RADIATION OF SINUSES USING PHOTONS <1 MEV |
| ICD10_proc | D9080ZZ | BEAM RADIATION OF HARD PALATE USING PHOTONS <1 MEV |
| ICD10_proc | D9090ZZ | BEAM RADIATION OF SOFT PALATE USING PHOTONS <1 MEV |
| ICD10_proc | DB10BBZ | LDR BRACHYTHERAPY OF TRACHEA USING PALLADIUM 103 |
| ICD10_proc | DB10BCZ | LDR BRACHYTHERAPY OF TRACHEA USING CALIFORNIUM 252 |
| ICD10_proc | DB10BYZ | LDR BRACHYTHERAPY OF TRACHEA USING OTH ISOTOPE |
| ICD10_proc | DB10B7Z | LDR BRACHYTHERAPY OF TRACHEA USING CESIUM 137 |
| ICD10_proc | DB10B8Z | LDR BRACHYTHERAPY OF TRACHEA USING IRIDIUM 192 |
| ICD10_proc | DB10B9Z | LDR BRACHYTHERAPY OF TRACHEA USING IODINE 125 |
| ICD10_proc | DB109BZ | HDR BRACHYTHERAPY OF TRACHEA USING PALLADIUM 103 |
| ICD10_proc | DB109CZ | HDR BRACHYTHERAPY OF TRACHEA USING CALIFORNIUM 252 |
| ICD10_proc | DB109YZ | HDR BRACHYTHERAPY OF TRACHEA USING OTH ISOTOPE |
| ICD10_proc | DB1097Z | HDR BRACHYTHERAPY OF TRACHEA USING CESIUM 137 |
| ICD10_proc | DB1098Z | HDR BRACHYTHERAPY OF TRACHEA USING IRIDIUM 192 |
| ICD10_proc | DB1099Z | HDR BRACHYTHERAPY OF TRACHEA USING IODINE 125 |
| ICD10_proc | DB11BBZ | LDR BRACHYTHERAPY OF BRONCHUS USING PALLADIUM 103 |
| ICD10_proc | DB11BCZ | LDR BRACHYTHERAPY OF BRONCHUS USING CALIFORNIUM 252 |
| ICD10_proc | DB11BYZ | LDR BRACHYTHERAPY OF BRONCHUS USING OTH ISOTOPE |
| ICD10_proc | DB11B7Z | LDR BRACHYTHERAPY OF BRONCHUS USING CESIUM 137 |
| ICD10_proc | DB11B8Z | LDR BRACHYTHERAPY OF BRONCHUS USING IRIDIUM 192 |
| ICD10_proc | DB11B9Z | LDR BRACHYTHERAPY OF BRONCHUS USING IODINE 125 |
| ICD10_proc | DB119BZ | HDR BRACHYTHERAPY OF BRONCHUS USING PALLADIUM 103 |
| ICD10_proc | DB119CZ | HDR BRACHYTHERAPY OF BRONCHUS USING CALIFORNIUM 252 |
| ICD10_proc | DB119YZ | HDR BRACHYTHERAPY OF BRONCHUS USING OTH ISOTOPE |
| ICD10_proc | DB1197Z | HDR BRACHYTHERAPY OF BRONCHUS USING CESIUM 137 |
| ICD10_proc | DB1198Z | HDR BRACHYTHERAPY OF BRONCHUS USING IRIDIUM 192 |
| ICD10_proc | DB1199Z | HDR BRACHYTHERAPY OF BRONCHUS USING IODINE 125 |
| ICD10_proc | DB12BBZ | LDR BRACHYTHERAPY OF LUNG USING PALLADIUM 103 |
| ICD10_proc | DB12BCZ | LDR BRACHYTHERAPY OF LUNG USING CALIFORNIUM 252 |
| ICD10_proc | DB12BYZ | LOW DOSE RATE (LDR) BRACHYTHERAPY OF LUNG USING OTH ISOTOPE |
| ICD10_proc | DB12B7Z | LOW DOSE RATE (LDR) BRACHYTHERAPY OF LUNG USING CESIUM 137 |
| ICD10_proc | DB12B8Z | LOW DOSE RATE (LDR) BRACHYTHERAPY OF LUNG USING IRIDIUM 192 |
| ICD10_proc | DB12B9Z | LOW DOSE RATE (LDR) BRACHYTHERAPY OF LUNG USING IODINE 125 |
| ICD10_proc | DB129BZ | HDR BRACHYTHERAPY OF LUNG USING PALLADIUM 103 |
| ICD10_proc | DB129CZ | HDR BRACHYTHERAPY OF LUNG USING CALIFORNIUM 252 |
| ICD10_proc | DB129YZ | HIGH DOSE RATE (HDR) BRACHYTHERAPY OF LUNG USING OTH ISOTOPE |
| ICD10_proc | DB1297Z | HIGH DOSE RATE (HDR) BRACHYTHERAPY OF LUNG USING CESIUM 137 |
| ICD10_proc | DB1298Z | HIGH DOSE RATE (HDR) BRACHYTHERAPY OF LUNG USING IRIDIUM 192 |
| ICD10_proc | DB1299Z | HIGH DOSE RATE (HDR) BRACHYTHERAPY OF LUNG USING IODINE 125 |
| ICD10_proc | DB15BBZ | LDR BRACHYTHERAPY OF PLEURA USING PALLADIUM 103 |
| ICD10_proc | DB15BCZ | LDR BRACHYTHERAPY OF PLEURA USING CALIFORNIUM 252 |
| ICD10_proc | DB15BYZ | LDR BRACHYTHERAPY OF PLEURA USING OTH ISOTOPE |
| ICD10_proc | DB15B7Z | LOW DOSE RATE (LDR) BRACHYTHERAPY OF PLEURA USING CESIUM 137 |
| ICD10_proc | DB15B8Z | LDR BRACHYTHERAPY OF PLEURA USING IRIDIUM 192 |
| ICD10_proc | DB15B9Z | LOW DOSE RATE (LDR) BRACHYTHERAPY OF PLEURA USING IODINE 125 |
| ICD10_proc | DB159BZ | HDR BRACHYTHERAPY OF PLEURA USING PALLADIUM 103 |
| ICD10_proc | DB159CZ | HDR BRACHYTHERAPY OF PLEURA USING CALIFORNIUM 252 |
| ICD10_proc | DB159YZ | HDR BRACHYTHERAPY OF PLEURA USING OTH ISOTOPE |
| ICD10_proc | DB1597Z | HDR BRACHYTHERAPY OF PLEURA USING CESIUM 137 |
| ICD10_proc | DB1598Z | HDR BRACHYTHERAPY OF PLEURA USING IRIDIUM 192 |
| ICD10_proc | DB1599Z | HDR BRACHYTHERAPY OF PLEURA USING IODINE 125 |
| ICD10_proc | DB16BBZ | LDR BRACHYTHERAPY OF MEDIASTINUM USING PALLADIUM 103 |
| ICD10_proc | DB16BCZ | LDR BRACHYTHERAPY OF MEDIASTINUM USING CALIFORNIUM 252 |
| ICD10_proc | DB16BYZ | LDR BRACHYTHERAPY OF MEDIASTINUM USING OTH ISOTOPE |
| ICD10_proc | DB16B7Z | LDR BRACHYTHERAPY OF MEDIASTINUM USING CESIUM 137 |
| ICD10_proc | DB16B8Z | LDR BRACHYTHERAPY OF MEDIASTINUM USING IRIDIUM 192 |
| ICD10_proc | DB16B9Z | LDR BRACHYTHERAPY OF MEDIASTINUM USING IODINE 125 |
| ICD10_proc | DB169BZ | HDR BRACHYTHERAPY OF MEDIASTINUM USING PALLADIUM 103 |
| ICD10_proc | DB169CZ | HDR BRACHYTHERAPY OF MEDIASTINUM USING CALIFORNIUM 252 |
| ICD10_proc | DB169YZ | HDR BRACHYTHERAPY OF MEDIASTINUM USING OTH ISOTOPE |
| ICD10_proc | DB1697Z | HDR BRACHYTHERAPY OF MEDIASTINUM USING CESIUM 137 |
| ICD10_proc | DB1698Z | HDR BRACHYTHERAPY OF MEDIASTINUM USING IRIDIUM 192 |
| ICD10_proc | DB1699Z | HDR BRACHYTHERAPY OF MEDIASTINUM USING IODINE 125 |
| ICD10_proc | DB17BBZ | LDR BRACHYTHERAPY OF CHEST WALL USING PALLADIUM 103 |
| ICD10_proc | DB17BCZ | LDR BRACHYTHERAPY OF CHEST WALL USING CALIFORNIUM 252 |
| ICD10_proc | DB17BYZ | LDR BRACHYTHERAPY OF CHEST WALL USING OTH ISOTOPE |
| ICD10_proc | DB17B7Z | LDR BRACHYTHERAPY OF CHEST WALL USING CESIUM 137 |
| ICD10_proc | DB17B8Z | LDR BRACHYTHERAPY OF CHEST WALL USING IRIDIUM 192 |
| ICD10_proc | DB17B9Z | LDR BRACHYTHERAPY OF CHEST WALL USING IODINE 125 |
| ICD10_proc | DB179BZ | HDR BRACHYTHERAPY OF CHEST WALL USING PALLADIUM 103 |
| ICD10_proc | DB179CZ | HDR BRACHYTHERAPY OF CHEST WALL USING CALIFORNIUM 252 |
| ICD10_proc | DB179YZ | HDR BRACHYTHERAPY OF CHEST WALL USING OTH ISOTOPE |
| ICD10_proc | DB1797Z | HDR BRACHYTHERAPY OF CHEST WALL USING CESIUM 137 |
| ICD10_proc | DB1798Z | HDR BRACHYTHERAPY OF CHEST WALL USING IRIDIUM 192 |
| ICD10_proc | DB1799Z | HDR BRACHYTHERAPY OF CHEST WALL USING IODINE 125 |
| ICD10_proc | DB18BBZ | LDR BRACHYTHERAPY OF DIAPHRAGM USING PALLADIUM 103 |
| ICD10_proc | DB18BCZ | LDR BRACHYTHERAPY OF DIAPHRAGM USING CALIFORNIUM 252 |
| ICD10_proc | DB18BYZ | LDR BRACHYTHERAPY OF DIAPHRAGM USING OTH ISOTOPE |
| ICD10_proc | DB18B7Z | LDR BRACHYTHERAPY OF DIAPHRAGM USING CESIUM 137 |
| ICD10_proc | DB18B8Z | LDR BRACHYTHERAPY OF DIAPHRAGM USING IRIDIUM 192 |
| ICD10_proc | DB18B9Z | LDR BRACHYTHERAPY OF DIAPHRAGM USING IODINE 125 |
| ICD10_proc | DB189BZ | HDR BRACHYTHERAPY OF DIAPHRAGM USING PALLADIUM 103 |
| ICD10_proc | DB189CZ | HDR BRACHYTHERAPY OF DIAPHRAGM USING CALIFORNIUM 252 |
| ICD10_proc | DB189YZ | HDR BRACHYTHERAPY OF DIAPHRAGM USING OTH ISOTOPE |
| ICD10_proc | DB1897Z | HDR BRACHYTHERAPY OF DIAPHRAGM USING CESIUM 137 |
| ICD10_proc | DB1898Z | HDR BRACHYTHERAPY OF DIAPHRAGM USING IRIDIUM 192 |
| ICD10_proc | DB1899Z | HDR BRACHYTHERAPY OF DIAPHRAGM USING IODINE 125 |
| ICD10_proc | DD10BBZ | LDR BRACHYTHERAPY OF ESOPHAGUS USING PALLADIUM 103 |
| ICD10_proc | DD10BCZ | LDR BRACHYTHERAPY OF ESOPHAGUS USING CALIFORNIUM 252 |
| ICD10_proc | DD10BYZ | LDR BRACHYTHERAPY OF ESOPHAGUS USING OTH ISOTOPE |
| ICD10_proc | DD10B7Z | LDR BRACHYTHERAPY OF ESOPHAGUS USING CESIUM 137 |
| ICD10_proc | DD10B8Z | LDR BRACHYTHERAPY OF ESOPHAGUS USING IRIDIUM 192 |
| ICD10_proc | DD10B9Z | LDR BRACHYTHERAPY OF ESOPHAGUS USING IODINE 125 |
| ICD10_proc | DD109BZ | HDR BRACHYTHERAPY OF ESOPHAGUS USING PALLADIUM 103 |
| ICD10_proc | DD109CZ | HDR BRACHYTHERAPY OF ESOPHAGUS USING CALIFORNIUM 252 |
| ICD10_proc | DD109YZ | HDR BRACHYTHERAPY OF ESOPHAGUS USING OTH ISOTOPE |
| ICD10_proc | DD1097Z | HDR BRACHYTHERAPY OF ESOPHAGUS USING CESIUM 137 |
| ICD10_proc | DD1098Z | HDR BRACHYTHERAPY OF ESOPHAGUS USING IRIDIUM 192 |
| ICD10_proc | DD1099Z | HDR BRACHYTHERAPY OF ESOPHAGUS USING IODINE 125 |
| ICD10_proc | DD11BBZ | LDR BRACHYTHERAPY OF STOMACH USING PALLADIUM 103 |
| ICD10_proc | DD11BCZ | LDR BRACHYTHERAPY OF STOMACH USING CALIFORNIUM 252 |
| ICD10_proc | DD11BYZ | LDR BRACHYTHERAPY OF STOMACH USING OTH ISOTOPE |
| ICD10_proc | DD11B7Z | LDR BRACHYTHERAPY OF STOMACH USING CESIUM 137 |
| ICD10_proc | DD11B8Z | LDR BRACHYTHERAPY OF STOMACH USING IRIDIUM 192 |
| ICD10_proc | DD11B9Z | LDR BRACHYTHERAPY OF STOMACH USING IODINE 125 |
| ICD10_proc | DD119BZ | HDR BRACHYTHERAPY OF STOMACH USING PALLADIUM 103 |
| ICD10_proc | DD119CZ | HDR BRACHYTHERAPY OF STOMACH USING CALIFORNIUM 252 |
| ICD10_proc | DD119YZ | HDR BRACHYTHERAPY OF STOMACH USING OTH ISOTOPE |
| ICD10_proc | DD1197Z | HDR BRACHYTHERAPY OF STOMACH USING CESIUM 137 |
| ICD10_proc | DD1198Z | HDR BRACHYTHERAPY OF STOMACH USING IRIDIUM 192 |
| ICD10_proc | DD1199Z | HDR BRACHYTHERAPY OF STOMACH USING IODINE 125 |
| ICD10_proc | DD12BBZ | LDR BRACHYTHERAPY OF DUODENUM USING PALLADIUM 103 |
| ICD10_proc | DD12BCZ | LDR BRACHYTHERAPY OF DUODENUM USING CALIFORNIUM 252 |
| ICD10_proc | DD12BYZ | LDR BRACHYTHERAPY OF DUODENUM USING OTH ISOTOPE |
| ICD10_proc | DD12B7Z | LDR BRACHYTHERAPY OF DUODENUM USING CESIUM 137 |
| ICD10_proc | DD12B8Z | LDR BRACHYTHERAPY OF DUODENUM USING IRIDIUM 192 |
| ICD10_proc | DD12B9Z | LDR BRACHYTHERAPY OF DUODENUM USING IODINE 125 |
| ICD10_proc | DD129BZ | HDR BRACHYTHERAPY OF DUODENUM USING PALLADIUM 103 |
| ICD10_proc | DD129CZ | HDR BRACHYTHERAPY OF DUODENUM USING CALIFORNIUM 252 |
| ICD10_proc | DD129YZ | HDR BRACHYTHERAPY OF DUODENUM USING OTH ISOTOPE |
| ICD10_proc | DD1297Z | HDR BRACHYTHERAPY OF DUODENUM USING CESIUM 137 |
| ICD10_proc | DD1298Z | HDR BRACHYTHERAPY OF DUODENUM USING IRIDIUM 192 |
| ICD10_proc | DD1299Z | HDR BRACHYTHERAPY OF DUODENUM USING IODINE 125 |
| ICD10_proc | DD13BBZ | LDR BRACHYTHERAPY OF JEJUNUM USING PALLADIUM 103 |
| ICD10_proc | DD13BCZ | LDR BRACHYTHERAPY OF JEJUNUM USING CALIFORNIUM 252 |
| ICD10_proc | DD13BYZ | LDR BRACHYTHERAPY OF JEJUNUM USING OTH ISOTOPE |
| ICD10_proc | DD13B7Z | LDR BRACHYTHERAPY OF JEJUNUM USING CESIUM 137 |
| ICD10_proc | DD13B8Z | LDR BRACHYTHERAPY OF JEJUNUM USING IRIDIUM 192 |
| ICD10_proc | DD13B9Z | LDR BRACHYTHERAPY OF JEJUNUM USING IODINE 125 |
| ICD10_proc | DD139BZ | HDR BRACHYTHERAPY OF JEJUNUM USING PALLADIUM 103 |
| ICD10_proc | DD139CZ | HDR BRACHYTHERAPY OF JEJUNUM USING CALIFORNIUM 252 |
| ICD10_proc | DD139YZ | HDR BRACHYTHERAPY OF JEJUNUM USING OTH ISOTOPE |
| ICD10_proc | DD1397Z | HDR BRACHYTHERAPY OF JEJUNUM USING CESIUM 137 |
| ICD10_proc | DD1398Z | HDR BRACHYTHERAPY OF JEJUNUM USING IRIDIUM 192 |
| ICD10_proc | DD1399Z | HDR BRACHYTHERAPY OF JEJUNUM USING IODINE 125 |
| ICD10_proc | DD14BBZ | LDR BRACHYTHERAPY OF ILEUM USING PALLADIUM 103 |
| ICD10_proc | DD14BCZ | LDR BRACHYTHERAPY OF ILEUM USING CALIFORNIUM 252 |
| ICD10_proc | DD14BYZ | LOW DOSE RATE (LDR) BRACHYTHERAPY OF ILEUM USING OTH ISOTOPE |
| ICD10_proc | DD14B7Z | LOW DOSE RATE (LDR) BRACHYTHERAPY OF ILEUM USING CESIUM 137 |
| ICD10_proc | DD14B8Z | LOW DOSE RATE (LDR) BRACHYTHERAPY OF ILEUM USING IRIDIUM 192 |
| ICD10_proc | DD14B9Z | LOW DOSE RATE (LDR) BRACHYTHERAPY OF ILEUM USING IODINE 125 |
| ICD10_proc | DD149BZ | HDR BRACHYTHERAPY OF ILEUM USING PALLADIUM 103 |
| ICD10_proc | DD149CZ | HDR BRACHYTHERAPY OF ILEUM USING CALIFORNIUM 252 |
| ICD10_proc | DD149YZ | HDR BRACHYTHERAPY OF ILEUM USING OTH ISOTOPE |
| ICD10_proc | DD1497Z | HIGH DOSE RATE (HDR) BRACHYTHERAPY OF ILEUM USING CESIUM 137 |
| ICD10_proc | DD1498Z | HDR BRACHYTHERAPY OF ILEUM USING IRIDIUM 192 |
| ICD10_proc | DD1499Z | HIGH DOSE RATE (HDR) BRACHYTHERAPY OF ILEUM USING IODINE 125 |
| ICD10_proc | DD15BBZ | LDR BRACHYTHERAPY OF COLON USING PALLADIUM 103 |
| ICD10_proc | DD15BCZ | LDR BRACHYTHERAPY OF COLON USING CALIFORNIUM 252 |
| ICD10_proc | DD15BYZ | LOW DOSE RATE (LDR) BRACHYTHERAPY OF COLON USING OTH ISOTOPE |
| ICD10_proc | DD15B7Z | LOW DOSE RATE (LDR) BRACHYTHERAPY OF COLON USING CESIUM 137 |
| ICD10_proc | DD15B8Z | LOW DOSE RATE (LDR) BRACHYTHERAPY OF COLON USING IRIDIUM 192 |
| ICD10_proc | DD15B9Z | LOW DOSE RATE (LDR) BRACHYTHERAPY OF COLON USING IODINE 125 |
| ICD10_proc | DD159BZ | HDR BRACHYTHERAPY OF COLON USING PALLADIUM 103 |
| ICD10_proc | DD159CZ | HDR BRACHYTHERAPY OF COLON USING CALIFORNIUM 252 |
| ICD10_proc | DD159YZ | HDR BRACHYTHERAPY OF COLON USING OTH ISOTOPE |
| ICD10_proc | DD1597Z | HIGH DOSE RATE (HDR) BRACHYTHERAPY OF COLON USING CESIUM 137 |
| ICD10_proc | DD1598Z | HDR BRACHYTHERAPY OF COLON USING IRIDIUM 192 |
| ICD10_proc | DD1599Z | HIGH DOSE RATE (HDR) BRACHYTHERAPY OF COLON USING IODINE 125 |
| ICD10_proc | DD17BBZ | LDR BRACHYTHERAPY OF RECTUM USING PALLADIUM 103 |
| ICD10_proc | DD17BCZ | LDR BRACHYTHERAPY OF RECTUM USING CALIFORNIUM 252 |
| ICD10_proc | DD17BYZ | LDR BRACHYTHERAPY OF RECTUM USING OTH ISOTOPE |
| ICD10_proc | DD17B7Z | LOW DOSE RATE (LDR) BRACHYTHERAPY OF RECTUM USING CESIUM 137 |
| ICD10_proc | DD17B8Z | LDR BRACHYTHERAPY OF RECTUM USING IRIDIUM 192 |
| ICD10_proc | DD17B9Z | LOW DOSE RATE (LDR) BRACHYTHERAPY OF RECTUM USING IODINE 125 |
| ICD10_proc | DD179BZ | HDR BRACHYTHERAPY OF RECTUM USING PALLADIUM 103 |
| ICD10_proc | DD179CZ | HDR BRACHYTHERAPY OF RECTUM USING CALIFORNIUM 252 |
| ICD10_proc | DD179YZ | HDR BRACHYTHERAPY OF RECTUM USING OTH ISOTOPE |
| ICD10_proc | DD1797Z | HDR BRACHYTHERAPY OF RECTUM USING CESIUM 137 |
| ICD10_proc | DD1798Z | HDR BRACHYTHERAPY OF RECTUM USING IRIDIUM 192 |
| ICD10_proc | DD1799Z | HDR BRACHYTHERAPY OF RECTUM USING IODINE 125 |
| ICD10_proc | DF10BBZ | LDR BRACHYTHERAPY OF LIVER USING PALLADIUM 103 |
| ICD10_proc | DF10BCZ | LDR BRACHYTHERAPY OF LIVER USING CALIFORNIUM 252 |
| ICD10_proc | DF10BYZ | LOW DOSE RATE (LDR) BRACHYTHERAPY OF LIVER USING OTH ISOTOPE |
| ICD10_proc | DF10B7Z | LOW DOSE RATE (LDR) BRACHYTHERAPY OF LIVER USING CESIUM 137 |
| ICD10_proc | DF10B8Z | LOW DOSE RATE (LDR) BRACHYTHERAPY OF LIVER USING IRIDIUM 192 |
| ICD10_proc | DF10B9Z | LOW DOSE RATE (LDR) BRACHYTHERAPY OF LIVER USING IODINE 125 |
| ICD10_proc | DF109BZ | HDR BRACHYTHERAPY OF LIVER USING PALLADIUM 103 |
| ICD10_proc | DF109CZ | HDR BRACHYTHERAPY OF LIVER USING CALIFORNIUM 252 |
| ICD10_proc | DF109YZ | HDR BRACHYTHERAPY OF LIVER USING OTH ISOTOPE |
| ICD10_proc | DF1097Z | HIGH DOSE RATE (HDR) BRACHYTHERAPY OF LIVER USING CESIUM 137 |
| ICD10_proc | DF1098Z | HDR BRACHYTHERAPY OF LIVER USING IRIDIUM 192 |
| ICD10_proc | DF1099Z | HIGH DOSE RATE (HDR) BRACHYTHERAPY OF LIVER USING IODINE 125 |
| ICD10_proc | DF11BBZ | LDR BRACHYTHERAPY OF GALLBLADDER USING PALLADIUM 103 |
| ICD10_proc | DF11BCZ | LDR BRACHYTHERAPY OF GALLBLADDER USING CALIFORNIUM 252 |
| ICD10_proc | DF11BYZ | LDR BRACHYTHERAPY OF GALLBLADDER USING OTH ISOTOPE |
| ICD10_proc | DF11B7Z | LDR BRACHYTHERAPY OF GALLBLADDER USING CESIUM 137 |
| ICD10_proc | DF11B8Z | LDR BRACHYTHERAPY OF GALLBLADDER USING IRIDIUM 192 |
| ICD10_proc | DF11B9Z | LDR BRACHYTHERAPY OF GALLBLADDER USING IODINE 125 |
| ICD10_proc | DF119BZ | HDR BRACHYTHERAPY OF GALLBLADDER USING PALLADIUM 103 |
| ICD10_proc | DF119CZ | HDR BRACHYTHERAPY OF GALLBLADDER USING CALIFORNIUM 252 |
| ICD10_proc | DF119YZ | HDR BRACHYTHERAPY OF GALLBLADDER USING OTH ISOTOPE |
| ICD10_proc | DF1197Z | HDR BRACHYTHERAPY OF GALLBLADDER USING CESIUM 137 |
| ICD10_proc | DF1198Z | HDR BRACHYTHERAPY OF GALLBLADDER USING IRIDIUM 192 |
| ICD10_proc | DF1199Z | HDR BRACHYTHERAPY OF GALLBLADDER USING IODINE 125 |
| ICD10_proc | DF12BBZ | LDR BRACHYTHERAPY OF BILE DUCTS USING PALLADIUM 103 |
| ICD10_proc | DF12BCZ | LDR BRACHYTHERAPY OF BILE DUCTS USING CALIFORNIUM 252 |
| ICD10_proc | DF12BYZ | LDR BRACHYTHERAPY OF BILE DUCTS USING OTH ISOTOPE |
| ICD10_proc | DF12B7Z | LDR BRACHYTHERAPY OF BILE DUCTS USING CESIUM 137 |
| ICD10_proc | DF12B8Z | LDR BRACHYTHERAPY OF BILE DUCTS USING IRIDIUM 192 |
| ICD10_proc | DF12B9Z | LDR BRACHYTHERAPY OF BILE DUCTS USING IODINE 125 |
| ICD10_proc | DF129BZ | HDR BRACHYTHERAPY OF BILE DUCTS USING PALLADIUM 103 |
| ICD10_proc | DF129CZ | HDR BRACHYTHERAPY OF BILE DUCTS USING CALIFORNIUM 252 |
| ICD10_proc | DF129YZ | HDR BRACHYTHERAPY OF BILE DUCTS USING OTH ISOTOPE |
| ICD10_proc | DF1297Z | HDR BRACHYTHERAPY OF BILE DUCTS USING CESIUM 137 |
| ICD10_proc | DF1298Z | HDR BRACHYTHERAPY OF BILE DUCTS USING IRIDIUM 192 |
| ICD10_proc | DF1299Z | HDR BRACHYTHERAPY OF BILE DUCTS USING IODINE 125 |
| ICD10_proc | DF13BBZ | LDR BRACHYTHERAPY OF PANCREAS USING PALLADIUM 103 |
| ICD10_proc | DF13BCZ | LDR BRACHYTHERAPY OF PANCREAS USING CALIFORNIUM 252 |
| ICD10_proc | DF13BYZ | LDR BRACHYTHERAPY OF PANCREAS USING OTH ISOTOPE |
| ICD10_proc | DF13B7Z | LDR BRACHYTHERAPY OF PANCREAS USING CESIUM 137 |
| ICD10_proc | DF13B8Z | LDR BRACHYTHERAPY OF PANCREAS USING IRIDIUM 192 |
| ICD10_proc | DF13B9Z | LDR BRACHYTHERAPY OF PANCREAS USING IODINE 125 |
| ICD10_proc | DF139BZ | HDR BRACHYTHERAPY OF PANCREAS USING PALLADIUM 103 |
| ICD10_proc | DF139CZ | HDR BRACHYTHERAPY OF PANCREAS USING CALIFORNIUM 252 |
| ICD10_proc | DF139YZ | HDR BRACHYTHERAPY OF PANCREAS USING OTH ISOTOPE |
| ICD10_proc | DF1397Z | HDR BRACHYTHERAPY OF PANCREAS USING CESIUM 137 |
| ICD10_proc | DF1398Z | HDR BRACHYTHERAPY OF PANCREAS USING IRIDIUM 192 |
| ICD10_proc | DF1399Z | HDR BRACHYTHERAPY OF PANCREAS USING IODINE 125 |
| ICD10_proc | DG10BBZ | LDR BRACHYTHERAPY OF PITUITARY GLAND USING PALLADIUM 103 |
| ICD10_proc | DG10BCZ | LDR BRACHYTHERAPY OF PITUITARY GLAND USING CALIFORNIUM 252 |
| ICD10_proc | DG10BYZ | LDR BRACHYTHERAPY OF PITUITARY GLAND USING OTH ISOTOPE |
| ICD10_proc | DG10B7Z | LDR BRACHYTHERAPY OF PITUITARY GLAND USING CESIUM 137 |
| ICD10_proc | DG10B8Z | LDR BRACHYTHERAPY OF PITUITARY GLAND USING IRIDIUM 192 |
| ICD10_proc | DG10B9Z | LDR BRACHYTHERAPY OF PITUITARY GLAND USING IODINE 125 |
| ICD10_proc | DG109BZ | HDR BRACHYTHERAPY OF PITUITARY GLAND USING PALLADIUM 103 |
| ICD10_proc | DG109CZ | HDR BRACHYTHERAPY OF PITUITARY GLAND USING CALIFORNIUM 252 |
| ICD10_proc | DG109YZ | HDR BRACHYTHERAPY OF PITUITARY GLAND USING OTH ISOTOPE |
| ICD10_proc | DG1097Z | HDR BRACHYTHERAPY OF PITUITARY GLAND USING CESIUM 137 |
| ICD10_proc | DG1098Z | HDR BRACHYTHERAPY OF PITUITARY GLAND USING IRIDIUM 192 |
| ICD10_proc | DG1099Z | HDR BRACHYTHERAPY OF PITUITARY GLAND USING IODINE 125 |
| ICD10_proc | DG11BBZ | LDR BRACHYTHERAPY OF PINEAL BODY USING PALLADIUM 103 |
| ICD10_proc | DG11BCZ | LDR BRACHYTHERAPY OF PINEAL BODY USING CALIFORNIUM 252 |
| ICD10_proc | DG11BYZ | LDR BRACHYTHERAPY OF PINEAL BODY USING OTH ISOTOPE |
| ICD10_proc | DG11B7Z | LDR BRACHYTHERAPY OF PINEAL BODY USING CESIUM 137 |
| ICD10_proc | DG11B8Z | LDR BRACHYTHERAPY OF PINEAL BODY USING IRIDIUM 192 |
| ICD10_proc | DG11B9Z | LDR BRACHYTHERAPY OF PINEAL BODY USING IODINE 125 |
| ICD10_proc | DG119BZ | HDR BRACHYTHERAPY OF PINEAL BODY USING PALLADIUM 103 |
| ICD10_proc | DG119CZ | HDR BRACHYTHERAPY OF PINEAL BODY USING CALIFORNIUM 252 |
| ICD10_proc | DG119YZ | HDR BRACHYTHERAPY OF PINEAL BODY USING OTH ISOTOPE |
| ICD10_proc | DG1197Z | HDR BRACHYTHERAPY OF PINEAL BODY USING CESIUM 137 |
| ICD10_proc | DG1198Z | HDR BRACHYTHERAPY OF PINEAL BODY USING IRIDIUM 192 |
| ICD10_proc | DG1199Z | HDR BRACHYTHERAPY OF PINEAL BODY USING IODINE 125 |
| ICD10_proc | DG12BBZ | LDR BRACHYTHERAPY OF ADRENAL GLANDS USING PALLADIUM 103 |
| ICD10_proc | DG12BCZ | LDR BRACHYTHERAPY OF ADRENAL GLANDS USING CALIFORNIUM 252 |
| ICD10_proc | DG12BYZ | LDR BRACHYTHERAPY OF ADRENAL GLANDS USING OTH ISOTOPE |
| ICD10_proc | DG12B7Z | LDR BRACHYTHERAPY OF ADRENAL GLANDS USING CESIUM 137 |
| ICD10_proc | DG12B8Z | LDR BRACHYTHERAPY OF ADRENAL GLANDS USING IRIDIUM 192 |
| ICD10_proc | DG12B9Z | LDR BRACHYTHERAPY OF ADRENAL GLANDS USING IODINE 125 |
| ICD10_proc | DG129BZ | HDR BRACHYTHERAPY OF ADRENAL GLANDS USING PALLADIUM 103 |
| ICD10_proc | DG129CZ | HDR BRACHYTHERAPY OF ADRENAL GLANDS USING CALIFORNIUM 252 |
| ICD10_proc | DG129YZ | HDR BRACHYTHERAPY OF ADRENAL GLANDS USING OTH ISOTOPE |
| ICD10_proc | DG1297Z | HDR BRACHYTHERAPY OF ADRENAL GLANDS USING CESIUM 137 |
| ICD10_proc | DG1298Z | HDR BRACHYTHERAPY OF ADRENAL GLANDS USING IRIDIUM 192 |
| ICD10_proc | DG1299Z | HDR BRACHYTHERAPY OF ADRENAL GLANDS USING IODINE 125 |
| ICD10_proc | DG14BBZ | LDR BRACHYTHERAPY OF PARATHYROID GLANDS USING PALLADIUM 103 |
| ICD10_proc | DG14BCZ | LDR BRACHYTHERAPY OF PARATHYROID GLANDS W CALIFORNIUM 252 |
| ICD10_proc | DG14BYZ | LDR BRACHYTHERAPY OF PARATHYROID GLANDS USING OTH ISOTOPE |
| ICD10_proc | DG14B7Z | LDR BRACHYTHERAPY OF PARATHYROID GLANDS USING CESIUM 137 |
| ICD10_proc | DG14B8Z | LDR BRACHYTHERAPY OF PARATHYROID GLANDS USING IRIDIUM 192 |
| ICD10_proc | DG14B9Z | LDR BRACHYTHERAPY OF PARATHYROID GLANDS USING IODINE 125 |
| ICD10_proc | DG149BZ | HDR BRACHYTHERAPY OF PARATHYROID GLANDS USING PALLADIUM 103 |
| ICD10_proc | DG149CZ | HDR BRACHYTHERAPY OF PARATHYROID GLANDS W CALIFORNIUM 252 |
| ICD10_proc | DG149YZ | HDR BRACHYTHERAPY OF PARATHYROID GLANDS USING OTH ISOTOPE |
| ICD10_proc | DG1497Z | HDR BRACHYTHERAPY OF PARATHYROID GLANDS USING CESIUM 137 |
| ICD10_proc | DG1498Z | HDR BRACHYTHERAPY OF PARATHYROID GLANDS USING IRIDIUM 192 |
| ICD10_proc | DG1499Z | HDR BRACHYTHERAPY OF PARATHYROID GLANDS USING IODINE 125 |
| ICD10_proc | DG15BBZ | LDR BRACHYTHERAPY OF THYROID USING PALLADIUM 103 |
| ICD10_proc | DG15BCZ | LDR BRACHYTHERAPY OF THYROID USING CALIFORNIUM 252 |
| ICD10_proc | DG15BYZ | LDR BRACHYTHERAPY OF THYROID USING OTH ISOTOPE |
| ICD10_proc | DG15B7Z | LDR BRACHYTHERAPY OF THYROID USING CESIUM 137 |
| ICD10_proc | DG15B8Z | LDR BRACHYTHERAPY OF THYROID USING IRIDIUM 192 |
| ICD10_proc | DG15B9Z | LDR BRACHYTHERAPY OF THYROID USING IODINE 125 |
| ICD10_proc | DG159BZ | HDR BRACHYTHERAPY OF THYROID USING PALLADIUM 103 |
| ICD10_proc | DG159CZ | HDR BRACHYTHERAPY OF THYROID USING CALIFORNIUM 252 |
| ICD10_proc | DG159YZ | HDR BRACHYTHERAPY OF THYROID USING OTH ISOTOPE |
| ICD10_proc | DG1597Z | HDR BRACHYTHERAPY OF THYROID USING CESIUM 137 |
| ICD10_proc | DG1598Z | HDR BRACHYTHERAPY OF THYROID USING IRIDIUM 192 |
| ICD10_proc | DG1599Z | HDR BRACHYTHERAPY OF THYROID USING IODINE 125 |
| ICD10_proc | DM10BBZ | LDR BRACHYTHERAPY OF L BREAST USING PALLADIUM 103 |
| ICD10_proc | DM10BCZ | LDR BRACHYTHERAPY OF L BREAST USING CALIFORNIUM 252 |
| ICD10_proc | DM10BYZ | LDR BRACHYTHERAPY OF L BREAST USING OTH ISOTOPE |
| ICD10_proc | DM10B7Z | LDR BRACHYTHERAPY OF L BREAST USING CESIUM 137 |
| ICD10_proc | DM10B8Z | LDR BRACHYTHERAPY OF L BREAST USING IRIDIUM 192 |
| ICD10_proc | DM10B9Z | LDR BRACHYTHERAPY OF L BREAST USING IODINE 125 |
| ICD10_proc | DM109BZ | HDR BRACHYTHERAPY OF L BREAST USING PALLADIUM 103 |
| ICD10_proc | DM109CZ | HDR BRACHYTHERAPY OF L BREAST USING CALIFORNIUM 252 |
| ICD10_proc | DM109YZ | HDR BRACHYTHERAPY OF L BREAST USING OTH ISOTOPE |
| ICD10_proc | DM1097Z | HDR BRACHYTHERAPY OF L BREAST USING CESIUM 137 |
| ICD10_proc | DM1098Z | HDR BRACHYTHERAPY OF L BREAST USING IRIDIUM 192 |
| ICD10_proc | DM1099Z | HDR BRACHYTHERAPY OF L BREAST USING IODINE 125 |
| ICD10_proc | DM11BBZ | LDR BRACHYTHERAPY OF R BREAST USING PALLADIUM 103 |
| ICD10_proc | DM11BCZ | LDR BRACHYTHERAPY OF R BREAST USING CALIFORNIUM 252 |
| ICD10_proc | DM11BYZ | LDR BRACHYTHERAPY OF R BREAST USING OTH ISOTOPE |
| ICD10_proc | DM11B7Z | LDR BRACHYTHERAPY OF R BREAST USING CESIUM 137 |
| ICD10_proc | DM11B8Z | LDR BRACHYTHERAPY OF R BREAST USING IRIDIUM 192 |
| ICD10_proc | DM11B9Z | LDR BRACHYTHERAPY OF R BREAST USING IODINE 125 |
| ICD10_proc | DM119BZ | HDR BRACHYTHERAPY OF R BREAST USING PALLADIUM 103 |
| ICD10_proc | DM119CZ | HDR BRACHYTHERAPY OF R BREAST USING CALIFORNIUM 252 |
| ICD10_proc | DM119YZ | HDR BRACHYTHERAPY OF R BREAST USING OTH ISOTOPE |
| ICD10_proc | DM1197Z | HDR BRACHYTHERAPY OF R BREAST USING CESIUM 137 |
| ICD10_proc | DM1198Z | HDR BRACHYTHERAPY OF R BREAST USING IRIDIUM 192 |
| ICD10_proc | DM1199Z | HDR BRACHYTHERAPY OF R BREAST USING IODINE 125 |
| ICD10_proc | DT10BBZ | LDR BRACHYTHERAPY OF KIDNEY USING PALLADIUM 103 |
| ICD10_proc | DT10BCZ | LDR BRACHYTHERAPY OF KIDNEY USING CALIFORNIUM 252 |
| ICD10_proc | DT10BYZ | LDR BRACHYTHERAPY OF KIDNEY USING OTH ISOTOPE |
| ICD10_proc | DT10B7Z | LOW DOSE RATE (LDR) BRACHYTHERAPY OF KIDNEY USING CESIUM 137 |
| ICD10_proc | DT10B8Z | LDR BRACHYTHERAPY OF KIDNEY USING IRIDIUM 192 |
| ICD10_proc | DT10B9Z | LOW DOSE RATE (LDR) BRACHYTHERAPY OF KIDNEY USING IODINE 125 |
| ICD10_proc | DT109BZ | HDR BRACHYTHERAPY OF KIDNEY USING PALLADIUM 103 |
| ICD10_proc | DT109CZ | HDR BRACHYTHERAPY OF KIDNEY USING CALIFORNIUM 252 |
| ICD10_proc | DT109YZ | HDR BRACHYTHERAPY OF KIDNEY USING OTH ISOTOPE |
| ICD10_proc | DT1097Z | HDR BRACHYTHERAPY OF KIDNEY USING CESIUM 137 |
| ICD10_proc | DT1098Z | HDR BRACHYTHERAPY OF KIDNEY USING IRIDIUM 192 |
| ICD10_proc | DT1099Z | HDR BRACHYTHERAPY OF KIDNEY USING IODINE 125 |
| ICD10_proc | DT11BBZ | LDR BRACHYTHERAPY OF URETER USING PALLADIUM 103 |
| ICD10_proc | DT11BCZ | LDR BRACHYTHERAPY OF URETER USING CALIFORNIUM 252 |
| ICD10_proc | DT11BYZ | LDR BRACHYTHERAPY OF URETER USING OTH ISOTOPE |
| ICD10_proc | DT11B7Z | LOW DOSE RATE (LDR) BRACHYTHERAPY OF URETER USING CESIUM 137 |
| ICD10_proc | DT11B8Z | LDR BRACHYTHERAPY OF URETER USING IRIDIUM 192 |
| ICD10_proc | DT11B9Z | LOW DOSE RATE (LDR) BRACHYTHERAPY OF URETER USING IODINE 125 |
| ICD10_proc | DT119BZ | HDR BRACHYTHERAPY OF URETER USING PALLADIUM 103 |
| ICD10_proc | DT119CZ | HDR BRACHYTHERAPY OF URETER USING CALIFORNIUM 252 |
| ICD10_proc | DT119YZ | HDR BRACHYTHERAPY OF URETER USING OTH ISOTOPE |
| ICD10_proc | DT1197Z | HDR BRACHYTHERAPY OF URETER USING CESIUM 137 |
| ICD10_proc | DT1198Z | HDR BRACHYTHERAPY OF URETER USING IRIDIUM 192 |
| ICD10_proc | DT1199Z | HDR BRACHYTHERAPY OF URETER USING IODINE 125 |
| ICD10_proc | DT12BBZ | LDR BRACHYTHERAPY OF BLADDER USING PALLADIUM 103 |
| ICD10_proc | DT12BCZ | LDR BRACHYTHERAPY OF BLADDER USING CALIFORNIUM 252 |
| ICD10_proc | DT12BYZ | LDR BRACHYTHERAPY OF BLADDER USING OTH ISOTOPE |
| ICD10_proc | DT12B7Z | LDR BRACHYTHERAPY OF BLADDER USING CESIUM 137 |
| ICD10_proc | DT12B8Z | LDR BRACHYTHERAPY OF BLADDER USING IRIDIUM 192 |
| ICD10_proc | DT12B9Z | LDR BRACHYTHERAPY OF BLADDER USING IODINE 125 |
| ICD10_proc | DT129BZ | HDR BRACHYTHERAPY OF BLADDER USING PALLADIUM 103 |
| ICD10_proc | DT129CZ | HDR BRACHYTHERAPY OF BLADDER USING CALIFORNIUM 252 |
| ICD10_proc | DT129YZ | HDR BRACHYTHERAPY OF BLADDER USING OTH ISOTOPE |
| ICD10_proc | DT1297Z | HDR BRACHYTHERAPY OF BLADDER USING CESIUM 137 |
| ICD10_proc | DT1298Z | HDR BRACHYTHERAPY OF BLADDER USING IRIDIUM 192 |
| ICD10_proc | DT1299Z | HDR BRACHYTHERAPY OF BLADDER USING IODINE 125 |
| ICD10_proc | DT13BBZ | LDR BRACHYTHERAPY OF URETHRA USING PALLADIUM 103 |
| ICD10_proc | DT13BCZ | LDR BRACHYTHERAPY OF URETHRA USING CALIFORNIUM 252 |
| ICD10_proc | DT13BYZ | LDR BRACHYTHERAPY OF URETHRA USING OTH ISOTOPE |
| ICD10_proc | DT13B7Z | LDR BRACHYTHERAPY OF URETHRA USING CESIUM 137 |
| ICD10_proc | DT13B8Z | LDR BRACHYTHERAPY OF URETHRA USING IRIDIUM 192 |
| ICD10_proc | DT13B9Z | LDR BRACHYTHERAPY OF URETHRA USING IODINE 125 |
| ICD10_proc | DT139BZ | HDR BRACHYTHERAPY OF URETHRA USING PALLADIUM 103 |
| ICD10_proc | DT139CZ | HDR BRACHYTHERAPY OF URETHRA USING CALIFORNIUM 252 |
| ICD10_proc | DT139YZ | HDR BRACHYTHERAPY OF URETHRA USING OTH ISOTOPE |
| ICD10_proc | DT1397Z | HDR BRACHYTHERAPY OF URETHRA USING CESIUM 137 |
| ICD10_proc | DT1398Z | HDR BRACHYTHERAPY OF URETHRA USING IRIDIUM 192 |
| ICD10_proc | DT1399Z | HDR BRACHYTHERAPY OF URETHRA USING IODINE 125 |
| ICD10_proc | DU10BBZ | LDR BRACHYTHERAPY OF OVARY USING PALLADIUM 103 |
| ICD10_proc | DU10BCZ | LDR BRACHYTHERAPY OF OVARY USING CALIFORNIUM 252 |
| ICD10_proc | DU10BYZ | LOW DOSE RATE (LDR) BRACHYTHERAPY OF OVARY USING OTH ISOTOPE |
| ICD10_proc | DU10B7Z | LOW DOSE RATE (LDR) BRACHYTHERAPY OF OVARY USING CESIUM 137 |
| ICD10_proc | DU10B8Z | LOW DOSE RATE (LDR) BRACHYTHERAPY OF OVARY USING IRIDIUM 192 |
| ICD10_proc | DU10B9Z | LOW DOSE RATE (LDR) BRACHYTHERAPY OF OVARY USING IODINE 125 |
| ICD10_proc | DU109BZ | HDR BRACHYTHERAPY OF OVARY USING PALLADIUM 103 |
| ICD10_proc | DU109CZ | HDR BRACHYTHERAPY OF OVARY USING CALIFORNIUM 252 |
| ICD10_proc | DU109YZ | HDR BRACHYTHERAPY OF OVARY USING OTH ISOTOPE |
| ICD10_proc | DU1097Z | HIGH DOSE RATE (HDR) BRACHYTHERAPY OF OVARY USING CESIUM 137 |
| ICD10_proc | DU1098Z | HDR BRACHYTHERAPY OF OVARY USING IRIDIUM 192 |
| ICD10_proc | DU1099Z | HIGH DOSE RATE (HDR) BRACHYTHERAPY OF OVARY USING IODINE 125 |
| ICD10_proc | DU11BBZ | LDR BRACHYTHERAPY OF CERVIX USING PALLADIUM 103 |
| ICD10_proc | DU11BCZ | LDR BRACHYTHERAPY OF CERVIX USING CALIFORNIUM 252 |
| ICD10_proc | DU11BYZ | LDR BRACHYTHERAPY OF CERVIX USING OTH ISOTOPE |
| ICD10_proc | DU11B7Z | LOW DOSE RATE (LDR) BRACHYTHERAPY OF CERVIX USING CESIUM 137 |
| ICD10_proc | DU11B8Z | LDR BRACHYTHERAPY OF CERVIX USING IRIDIUM 192 |
| ICD10_proc | DU11B9Z | LOW DOSE RATE (LDR) BRACHYTHERAPY OF CERVIX USING IODINE 125 |
| ICD10_proc | DU119BZ | HDR BRACHYTHERAPY OF CERVIX USING PALLADIUM 103 |
| ICD10_proc | DU119CZ | HDR BRACHYTHERAPY OF CERVIX USING CALIFORNIUM 252 |
| ICD10_proc | DU119YZ | HDR BRACHYTHERAPY OF CERVIX USING OTH ISOTOPE |
| ICD10_proc | DU1197Z | HDR BRACHYTHERAPY OF CERVIX USING CESIUM 137 |
| ICD10_proc | DU1198Z | HDR BRACHYTHERAPY OF CERVIX USING IRIDIUM 192 |
| ICD10_proc | DU1199Z | HDR BRACHYTHERAPY OF CERVIX USING IODINE 125 |
| ICD10_proc | DU12BBZ | LDR BRACHYTHERAPY OF UTERUS USING PALLADIUM 103 |
| ICD10_proc | DU12BCZ | LDR BRACHYTHERAPY OF UTERUS USING CALIFORNIUM 252 |
| ICD10_proc | DU12BYZ | LDR BRACHYTHERAPY OF UTERUS USING OTH ISOTOPE |
| ICD10_proc | DU12B7Z | LOW DOSE RATE (LDR) BRACHYTHERAPY OF UTERUS USING CESIUM 137 |
| ICD10_proc | DU12B8Z | LDR BRACHYTHERAPY OF UTERUS USING IRIDIUM 192 |
| ICD10_proc | DU12B9Z | LOW DOSE RATE (LDR) BRACHYTHERAPY OF UTERUS USING IODINE 125 |
| ICD10_proc | DU129BZ | HDR BRACHYTHERAPY OF UTERUS USING PALLADIUM 103 |
| ICD10_proc | DU129CZ | HDR BRACHYTHERAPY OF UTERUS USING CALIFORNIUM 252 |
| ICD10_proc | DU129YZ | HDR BRACHYTHERAPY OF UTERUS USING OTH ISOTOPE |
| ICD10_proc | DU1297Z | HDR BRACHYTHERAPY OF UTERUS USING CESIUM 137 |
| ICD10_proc | DU1298Z | HDR BRACHYTHERAPY OF UTERUS USING IRIDIUM 192 |
| ICD10_proc | DU1299Z | HDR BRACHYTHERAPY OF UTERUS USING IODINE 125 |
| ICD10_proc | DV10BBZ | LDR BRACHYTHERAPY OF PROSTATE USING PALLADIUM 103 |
| ICD10_proc | DV10BCZ | LDR BRACHYTHERAPY OF PROSTATE USING CALIFORNIUM 252 |
| ICD10_proc | DV10BYZ | LDR BRACHYTHERAPY OF PROSTATE USING OTH ISOTOPE |
| ICD10_proc | DV10B7Z | LDR BRACHYTHERAPY OF PROSTATE USING CESIUM 137 |
| ICD10_proc | DV10B8Z | LDR BRACHYTHERAPY OF PROSTATE USING IRIDIUM 192 |
| ICD10_proc | DV10B9Z | LDR BRACHYTHERAPY OF PROSTATE USING IODINE 125 |
| ICD10_proc | DV109BZ | HDR BRACHYTHERAPY OF PROSTATE USING PALLADIUM 103 |
| ICD10_proc | DV109CZ | HDR BRACHYTHERAPY OF PROSTATE USING CALIFORNIUM 252 |
| ICD10_proc | DV109YZ | HDR BRACHYTHERAPY OF PROSTATE USING OTH ISOTOPE |
| ICD10_proc | DV1097Z | HDR BRACHYTHERAPY OF PROSTATE USING CESIUM 137 |
| ICD10_proc | DV1098Z | HDR BRACHYTHERAPY OF PROSTATE USING IRIDIUM 192 |
| ICD10_proc | DV1099Z | HDR BRACHYTHERAPY OF PROSTATE USING IODINE 125 |
| ICD10_proc | DV11BBZ | LDR BRACHYTHERAPY OF TESTIS USING PALLADIUM 103 |
| ICD10_proc | DV11BCZ | LDR BRACHYTHERAPY OF TESTIS USING CALIFORNIUM 252 |
| ICD10_proc | DV11BYZ | LDR BRACHYTHERAPY OF TESTIS USING OTH ISOTOPE |
| ICD10_proc | DV11B7Z | LOW DOSE RATE (LDR) BRACHYTHERAPY OF TESTIS USING CESIUM 137 |
| ICD10_proc | DV11B8Z | LDR BRACHYTHERAPY OF TESTIS USING IRIDIUM 192 |
| ICD10_proc | DV11B9Z | LOW DOSE RATE (LDR) BRACHYTHERAPY OF TESTIS USING IODINE 125 |
| ICD10_proc | DV119BZ | HDR BRACHYTHERAPY OF TESTIS USING PALLADIUM 103 |
| ICD10_proc | DV119CZ | HDR BRACHYTHERAPY OF TESTIS USING CALIFORNIUM 252 |
| ICD10_proc | DV119YZ | HDR BRACHYTHERAPY OF TESTIS USING OTH ISOTOPE |
| ICD10_proc | DV1197Z | HDR BRACHYTHERAPY OF TESTIS USING CESIUM 137 |
| ICD10_proc | DV1198Z | HDR BRACHYTHERAPY OF TESTIS USING IRIDIUM 192 |
| ICD10_proc | DV1199Z | HDR BRACHYTHERAPY OF TESTIS USING IODINE 125 |
| ICD10_proc | DW11BBZ | LDR BRACHYTHERAPY OF HEAD & NECK USING PALLADIUM 103 |
| ICD10_proc | DW11BCZ | LDR BRACHYTHERAPY OF HEAD & NECK USING CALIFORNIUM 252 |
| ICD10_proc | DW11BYZ | LDR BRACHYTHERAPY OF HEAD & NECK USING OTH ISOTOPE |
| ICD10_proc | DW11B7Z | LDR BRACHYTHERAPY OF HEAD & NECK USING CESIUM 137 |
| ICD10_proc | DW11B8Z | LDR BRACHYTHERAPY OF HEAD & NECK USING IRIDIUM 192 |
| ICD10_proc | DW11B9Z | LDR BRACHYTHERAPY OF HEAD & NECK USING IODINE 125 |
| ICD10_proc | DW119BZ | HDR BRACHYTHERAPY OF HEAD & NECK USING PALLADIUM 103 |
| ICD10_proc | DW119CZ | HDR BRACHYTHERAPY OF HEAD & NECK USING CALIFORNIUM 252 |
| ICD10_proc | DW119YZ | HDR BRACHYTHERAPY OF HEAD & NECK USING OTH ISOTOPE |
| ICD10_proc | DW1197Z | HDR BRACHYTHERAPY OF HEAD & NECK USING CESIUM 137 |
| ICD10_proc | DW1198Z | HDR BRACHYTHERAPY OF HEAD & NECK USING IRIDIUM 192 |
| ICD10_proc | DW1199Z | HDR BRACHYTHERAPY OF HEAD & NECK USING IODINE 125 |
| ICD10_proc | DW12BBZ | LDR BRACHYTHERAPY OF CHEST USING PALLADIUM 103 |
| ICD10_proc | DW12BCZ | LDR BRACHYTHERAPY OF CHEST USING CALIFORNIUM 252 |
| ICD10_proc | DW12BYZ | LOW DOSE RATE (LDR) BRACHYTHERAPY OF CHEST USING OTH ISOTOPE |
| ICD10_proc | DW12B7Z | LOW DOSE RATE (LDR) BRACHYTHERAPY OF CHEST USING CESIUM 137 |
| ICD10_proc | DW12B8Z | LOW DOSE RATE (LDR) BRACHYTHERAPY OF CHEST USING IRIDIUM 192 |
| ICD10_proc | DW12B9Z | LOW DOSE RATE (LDR) BRACHYTHERAPY OF CHEST USING IODINE 125 |
| ICD10_proc | DW129BZ | HDR BRACHYTHERAPY OF CHEST USING PALLADIUM 103 |
| ICD10_proc | DW129CZ | HDR BRACHYTHERAPY OF CHEST USING CALIFORNIUM 252 |
| ICD10_proc | DW129YZ | HDR BRACHYTHERAPY OF CHEST USING OTH ISOTOPE |
| ICD10_proc | DW1297Z | HIGH DOSE RATE (HDR) BRACHYTHERAPY OF CHEST USING CESIUM 137 |
| ICD10_proc | DW1298Z | HDR BRACHYTHERAPY OF CHEST USING IRIDIUM 192 |
| ICD10_proc | DW1299Z | HIGH DOSE RATE (HDR) BRACHYTHERAPY OF CHEST USING IODINE 125 |
| ICD10_proc | DW13BBZ | LOW DOSE RATE (LDR) BRACHYTHERAPY OF ABD USING PALLADIUM 103 |
| ICD10_proc | DW13BCZ | LDR BRACHYTHERAPY OF ABD USING CALIFORNIUM 252 |
| ICD10_proc | DW13BYZ | LOW DOSE RATE (LDR) BRACHYTHERAPY OF ABD USING OTH ISOTOPE |
| ICD10_proc | DW13B7Z | LOW DOSE RATE (LDR) BRACHYTHERAPY OF ABD USING CESIUM 137 |
| ICD10_proc | DW13B8Z | LOW DOSE RATE (LDR) BRACHYTHERAPY OF ABD USING IRIDIUM 192 |
| ICD10_proc | DW13B9Z | LOW DOSE RATE (LDR) BRACHYTHERAPY OF ABD USING IODINE 125 |
| ICD10_proc | DW139BZ | HDR BRACHYTHERAPY OF ABD USING PALLADIUM 103 |
| ICD10_proc | DW139CZ | HDR BRACHYTHERAPY OF ABD USING CALIFORNIUM 252 |
| ICD10_proc | DW139YZ | HIGH DOSE RATE (HDR) BRACHYTHERAPY OF ABD USING OTH ISOTOPE |
| ICD10_proc | DW1397Z | HIGH DOSE RATE (HDR) BRACHYTHERAPY OF ABD USING CESIUM 137 |
| ICD10_proc | DW1398Z | HIGH DOSE RATE (HDR) BRACHYTHERAPY OF ABD USING IRIDIUM 192 |
| ICD10_proc | DW1399Z | HIGH DOSE RATE (HDR) BRACHYTHERAPY OF ABD USING IODINE 125 |
| ICD10_proc | DW16BBZ | LDR BRACHYTHERAPY OF PELVIC REGION USING PALLADIUM 103 |
| ICD10_proc | DW16BCZ | LDR BRACHYTHERAPY OF PELVIC REGION USING CALIFORNIUM 252 |
| ICD10_proc | DW16BYZ | LDR BRACHYTHERAPY OF PELVIC REGION USING OTH ISOTOPE |
| ICD10_proc | DW16B7Z | LDR BRACHYTHERAPY OF PELVIC REGION USING CESIUM 137 |
| ICD10_proc | DW16B8Z | LDR BRACHYTHERAPY OF PELVIC REGION USING IRIDIUM 192 |
| ICD10_proc | DW16B9Z | LDR BRACHYTHERAPY OF PELVIC REGION USING IODINE 125 |
| ICD10_proc | DW169BZ | HDR BRACHYTHERAPY OF PELVIC REGION USING PALLADIUM 103 |
| ICD10_proc | DW169CZ | HDR BRACHYTHERAPY OF PELVIC REGION USING CALIFORNIUM 252 |
| ICD10_proc | DW169YZ | HDR BRACHYTHERAPY OF PELVIC REGION USING OTH ISOTOPE |
| ICD10_proc | DW1697Z | HDR BRACHYTHERAPY OF PELVIC REGION USING CESIUM 137 |
| ICD10_proc | DW1698Z | HDR BRACHYTHERAPY OF PELVIC REGION USING IRIDIUM 192 |
| ICD10_proc | DW1699Z | HDR BRACHYTHERAPY OF PELVIC REGION USING IODINE 125 |
| ICD10_proc | D010BBZ | LDR BRACHYTHERAPY OF BRAIN USING PALLADIUM 103 |
| ICD10_proc | D010BCZ | LDR BRACHYTHERAPY OF BRAIN USING CALIFORNIUM 252 |
| ICD10_proc | D010BYZ | LOW DOSE RATE (LDR) BRACHYTHERAPY OF BRAIN USING OTH ISOTOPE |
| ICD10_proc | D010B7Z | LOW DOSE RATE (LDR) BRACHYTHERAPY OF BRAIN USING CESIUM 137 |
| ICD10_proc | D010B8Z | LOW DOSE RATE (LDR) BRACHYTHERAPY OF BRAIN USING IRIDIUM 192 |
| ICD10_proc | D010B9Z | LOW DOSE RATE (LDR) BRACHYTHERAPY OF BRAIN USING IODINE 125 |
| ICD10_proc | D0109BZ | HDR BRACHYTHERAPY OF BRAIN USING PALLADIUM 103 |
| ICD10_proc | D0109CZ | HDR BRACHYTHERAPY OF BRAIN USING CALIFORNIUM 252 |
| ICD10_proc | D0109YZ | HDR BRACHYTHERAPY OF BRAIN USING OTH ISOTOPE |
| ICD10_proc | D01097Z | HIGH DOSE RATE (HDR) BRACHYTHERAPY OF BRAIN USING CESIUM 137 |
| ICD10_proc | D01098Z | HDR BRACHYTHERAPY OF BRAIN USING IRIDIUM 192 |
| ICD10_proc | D01099Z | HIGH DOSE RATE (HDR) BRACHYTHERAPY OF BRAIN USING IODINE 125 |
| ICD10_proc | D011BBZ | LDR BRACHYTHERAPY OF BRAIN STEM USING PALLADIUM 103 |
| ICD10_proc | D011BCZ | LDR BRACHYTHERAPY OF BRAIN STEM USING CALIFORNIUM 252 |
| ICD10_proc | D011BYZ | LDR BRACHYTHERAPY OF BRAIN STEM USING OTH ISOTOPE |
| ICD10_proc | D011B7Z | LDR BRACHYTHERAPY OF BRAIN STEM USING CESIUM 137 |
| ICD10_proc | D011B8Z | LDR BRACHYTHERAPY OF BRAIN STEM USING IRIDIUM 192 |
| ICD10_proc | D011B9Z | LDR BRACHYTHERAPY OF BRAIN STEM USING IODINE 125 |
| ICD10_proc | D0119BZ | HDR BRACHYTHERAPY OF BRAIN STEM USING PALLADIUM 103 |
| ICD10_proc | D0119CZ | HDR BRACHYTHERAPY OF BRAIN STEM USING CALIFORNIUM 252 |
| ICD10_proc | D0119YZ | HDR BRACHYTHERAPY OF BRAIN STEM USING OTH ISOTOPE |
| ICD10_proc | D01197Z | HDR BRACHYTHERAPY OF BRAIN STEM USING CESIUM 137 |
| ICD10_proc | D01198Z | HDR BRACHYTHERAPY OF BRAIN STEM USING IRIDIUM 192 |
| ICD10_proc | D01199Z | HDR BRACHYTHERAPY OF BRAIN STEM USING IODINE 125 |
| ICD10_proc | D016BBZ | LDR BRACHYTHERAPY OF SPINAL CORD USING PALLADIUM 103 |
| ICD10_proc | D016BCZ | LDR BRACHYTHERAPY OF SPINAL CORD USING CALIFORNIUM 252 |
| ICD10_proc | D016BYZ | LDR BRACHYTHERAPY OF SPINAL CORD USING OTH ISOTOPE |
| ICD10_proc | D016B7Z | LDR BRACHYTHERAPY OF SPINAL CORD USING CESIUM 137 |
| ICD10_proc | D016B8Z | LDR BRACHYTHERAPY OF SPINAL CORD USING IRIDIUM 192 |
| ICD10_proc | D016B9Z | LDR BRACHYTHERAPY OF SPINAL CORD USING IODINE 125 |
| ICD10_proc | D0169BZ | HDR BRACHYTHERAPY OF SPINAL CORD USING PALLADIUM 103 |
| ICD10_proc | D0169CZ | HDR BRACHYTHERAPY OF SPINAL CORD USING CALIFORNIUM 252 |
| ICD10_proc | D0169YZ | HDR BRACHYTHERAPY OF SPINAL CORD USING OTH ISOTOPE |
| ICD10_proc | D01697Z | HDR BRACHYTHERAPY OF SPINAL CORD USING CESIUM 137 |
| ICD10_proc | D01698Z | HDR BRACHYTHERAPY OF SPINAL CORD USING IRIDIUM 192 |
| ICD10_proc | D01699Z | HDR BRACHYTHERAPY OF SPINAL CORD USING IODINE 125 |
| ICD10_proc | D017BBZ | LDR BRACHYTHERAPY OF PERIPHERAL NERVE USING PALLADIUM 103 |
| ICD10_proc | D017BCZ | LDR BRACHYTHERAPY OF PERIPHERAL NERVE USING CALIFORNIUM 252 |
| ICD10_proc | D017BYZ | LDR BRACHYTHERAPY OF PERIPHERAL NERVE USING OTH ISOTOPE |
| ICD10_proc | D017B7Z | LDR BRACHYTHERAPY OF PERIPHERAL NERVE USING CESIUM 137 |
| ICD10_proc | D017B8Z | LDR BRACHYTHERAPY OF PERIPHERAL NERVE USING IRIDIUM 192 |
| ICD10_proc | D017B9Z | LDR BRACHYTHERAPY OF PERIPHERAL NERVE USING IODINE 125 |
| ICD10_proc | D0179BZ | HDR BRACHYTHERAPY OF PERIPHERAL NERVE USING PALLADIUM 103 |
| ICD10_proc | D0179CZ | HDR BRACHYTHERAPY OF PERIPHERAL NERVE USING CALIFORNIUM 252 |
| ICD10_proc | D0179YZ | HDR BRACHYTHERAPY OF PERIPHERAL NERVE USING OTH ISOTOPE |
| ICD10_proc | D01797Z | HDR BRACHYTHERAPY OF PERIPHERAL NERVE USING CESIUM 137 |
| ICD10_proc | D01798Z | HDR BRACHYTHERAPY OF PERIPHERAL NERVE USING IRIDIUM 192 |
| ICD10_proc | D01799Z | HDR BRACHYTHERAPY OF PERIPHERAL NERVE USING IODINE 125 |
| ICD10_proc | D710BBZ | LDR BRACHYTHERAPY OF BONE MARROW USING PALLADIUM 103 |
| ICD10_proc | D710BCZ | LDR BRACHYTHERAPY OF BONE MARROW USING CALIFORNIUM 252 |
| ICD10_proc | D710BYZ | LDR BRACHYTHERAPY OF BONE MARROW USING OTH ISOTOPE |
| ICD10_proc | D710B7Z | LDR BRACHYTHERAPY OF BONE MARROW USING CESIUM 137 |
| ICD10_proc | D710B8Z | LDR BRACHYTHERAPY OF BONE MARROW USING IRIDIUM 192 |
| ICD10_proc | D710B9Z | LDR BRACHYTHERAPY OF BONE MARROW USING IODINE 125 |
| ICD10_proc | D7109BZ | HDR BRACHYTHERAPY OF BONE MARROW USING PALLADIUM 103 |
| ICD10_proc | D7109CZ | HDR BRACHYTHERAPY OF BONE MARROW USING CALIFORNIUM 252 |
| ICD10_proc | D7109YZ | HDR BRACHYTHERAPY OF BONE MARROW USING OTH ISOTOPE |
| ICD10_proc | D71097Z | HDR BRACHYTHERAPY OF BONE MARROW USING CESIUM 137 |
| ICD10_proc | D71098Z | HDR BRACHYTHERAPY OF BONE MARROW USING IRIDIUM 192 |
| ICD10_proc | D71099Z | HDR BRACHYTHERAPY OF BONE MARROW USING IODINE 125 |
| ICD10_proc | D711BBZ | LDR BRACHYTHERAPY OF THYMUS USING PALLADIUM 103 |
| ICD10_proc | D711BCZ | LDR BRACHYTHERAPY OF THYMUS USING CALIFORNIUM 252 |
| ICD10_proc | D711BYZ | LDR BRACHYTHERAPY OF THYMUS USING OTH ISOTOPE |
| ICD10_proc | D711B7Z | LOW DOSE RATE (LDR) BRACHYTHERAPY OF THYMUS USING CESIUM 137 |
| ICD10_proc | D711B8Z | LDR BRACHYTHERAPY OF THYMUS USING IRIDIUM 192 |
| ICD10_proc | D711B9Z | LOW DOSE RATE (LDR) BRACHYTHERAPY OF THYMUS USING IODINE 125 |
| ICD10_proc | D7119BZ | HDR BRACHYTHERAPY OF THYMUS USING PALLADIUM 103 |
| ICD10_proc | D7119CZ | HDR BRACHYTHERAPY OF THYMUS USING CALIFORNIUM 252 |
| ICD10_proc | D7119YZ | HDR BRACHYTHERAPY OF THYMUS USING OTH ISOTOPE |
| ICD10_proc | D71197Z | HDR BRACHYTHERAPY OF THYMUS USING CESIUM 137 |
| ICD10_proc | D71198Z | HDR BRACHYTHERAPY OF THYMUS USING IRIDIUM 192 |
| ICD10_proc | D71199Z | HDR BRACHYTHERAPY OF THYMUS USING IODINE 125 |
| ICD10_proc | D712BBZ | LDR BRACHYTHERAPY OF SPLEEN USING PALLADIUM 103 |
| ICD10_proc | D712BCZ | LDR BRACHYTHERAPY OF SPLEEN USING CALIFORNIUM 252 |
| ICD10_proc | D712BYZ | LDR BRACHYTHERAPY OF SPLEEN USING OTH ISOTOPE |
| ICD10_proc | D712B7Z | LOW DOSE RATE (LDR) BRACHYTHERAPY OF SPLEEN USING CESIUM 137 |
| ICD10_proc | D712B8Z | LDR BRACHYTHERAPY OF SPLEEN USING IRIDIUM 192 |
| ICD10_proc | D712B9Z | LOW DOSE RATE (LDR) BRACHYTHERAPY OF SPLEEN USING IODINE 125 |
| ICD10_proc | D7129BZ | HDR BRACHYTHERAPY OF SPLEEN USING PALLADIUM 103 |
| ICD10_proc | D7129CZ | HDR BRACHYTHERAPY OF SPLEEN USING CALIFORNIUM 252 |
| ICD10_proc | D7129YZ | HDR BRACHYTHERAPY OF SPLEEN USING OTH ISOTOPE |
| ICD10_proc | D71297Z | HDR BRACHYTHERAPY OF SPLEEN USING CESIUM 137 |
| ICD10_proc | D71298Z | HDR BRACHYTHERAPY OF SPLEEN USING IRIDIUM 192 |
| ICD10_proc | D71299Z | HDR BRACHYTHERAPY OF SPLEEN USING IODINE 125 |
| ICD10_proc | D713BBZ | LDR BRACHYTHERAPY OF NECK LYMPH USING PALLADIUM 103 |
| ICD10_proc | D713BCZ | LDR BRACHYTHERAPY OF NECK LYMPH USING CALIFORNIUM 252 |
| ICD10_proc | D713BYZ | LDR BRACHYTHERAPY OF NECK LYMPH USING OTH ISOTOPE |
| ICD10_proc | D713B7Z | LDR BRACHYTHERAPY OF NECK LYMPH USING CESIUM 137 |
| ICD10_proc | D713B8Z | LDR BRACHYTHERAPY OF NECK LYMPH USING IRIDIUM 192 |
| ICD10_proc | D713B9Z | LDR BRACHYTHERAPY OF NECK LYMPH USING IODINE 125 |
| ICD10_proc | D7139BZ | HDR BRACHYTHERAPY OF NECK LYMPH USING PALLADIUM 103 |
| ICD10_proc | D7139CZ | HDR BRACHYTHERAPY OF NECK LYMPH USING CALIFORNIUM 252 |
| ICD10_proc | D7139YZ | HDR BRACHYTHERAPY OF NECK LYMPH USING OTH ISOTOPE |
| ICD10_proc | D71397Z | HDR BRACHYTHERAPY OF NECK LYMPH USING CESIUM 137 |
| ICD10_proc | D71398Z | HDR BRACHYTHERAPY OF NECK LYMPH USING IRIDIUM 192 |
| ICD10_proc | D71399Z | HDR BRACHYTHERAPY OF NECK LYMPH USING IODINE 125 |
| ICD10_proc | D714BBZ | LDR BRACHYTHERAPY OF AXILLA LYMPH USING PALLADIUM 103 |
| ICD10_proc | D714BCZ | LDR BRACHYTHERAPY OF AXILLA LYMPH USING CALIFORNIUM 252 |
| ICD10_proc | D714BYZ | LDR BRACHYTHERAPY OF AXILLA LYMPH USING OTH ISOTOPE |
| ICD10_proc | D714B7Z | LDR BRACHYTHERAPY OF AXILLA LYMPH USING CESIUM 137 |
| ICD10_proc | D714B8Z | LDR BRACHYTHERAPY OF AXILLA LYMPH USING IRIDIUM 192 |
| ICD10_proc | D714B9Z | LDR BRACHYTHERAPY OF AXILLA LYMPH USING IODINE 125 |
| ICD10_proc | D7149BZ | HDR BRACHYTHERAPY OF AXILLA LYMPH USING PALLADIUM 103 |
| ICD10_proc | D7149CZ | HDR BRACHYTHERAPY OF AXILLA LYMPH USING CALIFORNIUM 252 |
| ICD10_proc | D7149YZ | HDR BRACHYTHERAPY OF AXILLA LYMPH USING OTH ISOTOPE |
| ICD10_proc | D71497Z | HDR BRACHYTHERAPY OF AXILLA LYMPH USING CESIUM 137 |
| ICD10_proc | D71498Z | HDR BRACHYTHERAPY OF AXILLA LYMPH USING IRIDIUM 192 |
| ICD10_proc | D71499Z | HDR BRACHYTHERAPY OF AXILLA LYMPH USING IODINE 125 |
| ICD10_proc | D715BBZ | LDR BRACHYTHERAPY OF THORAX LYMPH USING PALLADIUM 103 |
| ICD10_proc | D715BCZ | LDR BRACHYTHERAPY OF THORAX LYMPH USING CALIFORNIUM 252 |
| ICD10_proc | D715BYZ | LDR BRACHYTHERAPY OF THORAX LYMPH USING OTH ISOTOPE |
| ICD10_proc | D715B7Z | LDR BRACHYTHERAPY OF THORAX LYMPH USING CESIUM 137 |
| ICD10_proc | D715B8Z | LDR BRACHYTHERAPY OF THORAX LYMPH USING IRIDIUM 192 |
| ICD10_proc | D715B9Z | LDR BRACHYTHERAPY OF THORAX LYMPH USING IODINE 125 |
| ICD10_proc | D7159BZ | HDR BRACHYTHERAPY OF THORAX LYMPH USING PALLADIUM 103 |
| ICD10_proc | D7159CZ | HDR BRACHYTHERAPY OF THORAX LYMPH USING CALIFORNIUM 252 |
| ICD10_proc | D7159YZ | HDR BRACHYTHERAPY OF THORAX LYMPH USING OTH ISOTOPE |
| ICD10_proc | D71597Z | HDR BRACHYTHERAPY OF THORAX LYMPH USING CESIUM 137 |
| ICD10_proc | D71598Z | HDR BRACHYTHERAPY OF THORAX LYMPH USING IRIDIUM 192 |
| ICD10_proc | D71599Z | HDR BRACHYTHERAPY OF THORAX LYMPH USING IODINE 125 |
| ICD10_proc | D716BBZ | LDR BRACHYTHERAPY OF ABD LYMPH USING PALLADIUM 103 |
| ICD10_proc | D716BCZ | LDR BRACHYTHERAPY OF ABD LYMPH USING CALIFORNIUM 252 |
| ICD10_proc | D716BYZ | LDR BRACHYTHERAPY OF ABD LYMPH USING OTH ISOTOPE |
| ICD10_proc | D716B7Z | LDR BRACHYTHERAPY OF ABD LYMPH USING CESIUM 137 |
| ICD10_proc | D716B8Z | LDR BRACHYTHERAPY OF ABD LYMPH USING IRIDIUM 192 |
| ICD10_proc | D716B9Z | LDR BRACHYTHERAPY OF ABD LYMPH USING IODINE 125 |
| ICD10_proc | D7169BZ | HDR BRACHYTHERAPY OF ABD LYMPH USING PALLADIUM 103 |
| ICD10_proc | D7169CZ | HDR BRACHYTHERAPY OF ABD LYMPH USING CALIFORNIUM 252 |
| ICD10_proc | D7169YZ | HDR BRACHYTHERAPY OF ABD LYMPH USING OTH ISOTOPE |
| ICD10_proc | D71697Z | HDR BRACHYTHERAPY OF ABD LYMPH USING CESIUM 137 |
| ICD10_proc | D71698Z | HDR BRACHYTHERAPY OF ABD LYMPH USING IRIDIUM 192 |
| ICD10_proc | D71699Z | HDR BRACHYTHERAPY OF ABD LYMPH USING IODINE 125 |
| ICD10_proc | D717BBZ | LDR BRACHYTHERAPY OF PELVIS LYMPH USING PALLADIUM 103 |
| ICD10_proc | D717BCZ | LDR BRACHYTHERAPY OF PELVIS LYMPH USING CALIFORNIUM 252 |
| ICD10_proc | D717BYZ | LDR BRACHYTHERAPY OF PELVIS LYMPH USING OTH ISOTOPE |
| ICD10_proc | D717B7Z | LDR BRACHYTHERAPY OF PELVIS LYMPH USING CESIUM 137 |
| ICD10_proc | D717B8Z | LDR BRACHYTHERAPY OF PELVIS LYMPH USING IRIDIUM 192 |
| ICD10_proc | D717B9Z | LDR BRACHYTHERAPY OF PELVIS LYMPH USING IODINE 125 |
| ICD10_proc | D7179BZ | HDR BRACHYTHERAPY OF PELVIS LYMPH USING PALLADIUM 103 |
| ICD10_proc | D7179CZ | HDR BRACHYTHERAPY OF PELVIS LYMPH USING CALIFORNIUM 252 |
| ICD10_proc | D7179YZ | HDR BRACHYTHERAPY OF PELVIS LYMPH USING OTH ISOTOPE |
| ICD10_proc | D71797Z | HDR BRACHYTHERAPY OF PELVIS LYMPH USING CESIUM 137 |
| ICD10_proc | D71798Z | HDR BRACHYTHERAPY OF PELVIS LYMPH USING IRIDIUM 192 |
| ICD10_proc | D71799Z | HDR BRACHYTHERAPY OF PELVIS LYMPH USING IODINE 125 |
| ICD10_proc | D718BBZ | LDR BRACHYTHERAPY OF INGUINAL LYMPH USING PALLADIUM 103 |
| ICD10_proc | D718BCZ | LDR BRACHYTHERAPY OF INGUINAL LYMPH USING CALIFORNIUM 252 |
| ICD10_proc | D718BYZ | LDR BRACHYTHERAPY OF INGUINAL LYMPH USING OTH ISOTOPE |
| ICD10_proc | D718B7Z | LDR BRACHYTHERAPY OF INGUINAL LYMPH USING CESIUM 137 |
| ICD10_proc | D718B8Z | LDR BRACHYTHERAPY OF INGUINAL LYMPH USING IRIDIUM 192 |
| ICD10_proc | D718B9Z | LDR BRACHYTHERAPY OF INGUINAL LYMPH USING IODINE 125 |
| ICD10_proc | D7189BZ | HDR BRACHYTHERAPY OF INGUINAL LYMPH USING PALLADIUM 103 |
| ICD10_proc | D7189CZ | HDR BRACHYTHERAPY OF INGUINAL LYMPH USING CALIFORNIUM 252 |
| ICD10_proc | D7189YZ | HDR BRACHYTHERAPY OF INGUINAL LYMPH USING OTH ISOTOPE |
| ICD10_proc | D71897Z | HDR BRACHYTHERAPY OF INGUINAL LYMPH USING CESIUM 137 |
| ICD10_proc | D71898Z | HDR BRACHYTHERAPY OF INGUINAL LYMPH USING IRIDIUM 192 |
| ICD10_proc | D71899Z | HDR BRACHYTHERAPY OF INGUINAL LYMPH USING IODINE 125 |
| ICD10_proc | D810BBZ | LOW DOSE RATE (LDR) BRACHYTHERAPY OF EYE USING PALLADIUM 103 |
| ICD10_proc | D810BCZ | LDR BRACHYTHERAPY OF EYE USING CALIFORNIUM 252 |
| ICD10_proc | D810BYZ | LOW DOSE RATE (LDR) BRACHYTHERAPY OF EYE USING OTHER ISOTOPE |
| ICD10_proc | D810B7Z | LOW DOSE RATE (LDR) BRACHYTHERAPY OF EYE USING CESIUM 137 |
| ICD10_proc | D810B8Z | LOW DOSE RATE (LDR) BRACHYTHERAPY OF EYE USING IRIDIUM 192 |
| ICD10_proc | D810B9Z | LOW DOSE RATE (LDR) BRACHYTHERAPY OF EYE USING IODINE 125 |
| ICD10_proc | D8109BZ | HDR BRACHYTHERAPY OF EYE USING PALLADIUM 103 |
| ICD10_proc | D8109CZ | HDR BRACHYTHERAPY OF EYE USING CALIFORNIUM 252 |
| ICD10_proc | D8109YZ | HIGH DOSE RATE (HDR) BRACHYTHERAPY OF EYE USING OTH ISOTOPE |
| ICD10_proc | D81097Z | HIGH DOSE RATE (HDR) BRACHYTHERAPY OF EYE USING CESIUM 137 |
| ICD10_proc | D81098Z | HIGH DOSE RATE (HDR) BRACHYTHERAPY OF EYE USING IRIDIUM 192 |
| ICD10_proc | D81099Z | HIGH DOSE RATE (HDR) BRACHYTHERAPY OF EYE USING IODINE 125 |
| ICD10_proc | D91BBBZ | LDR BRACHYTHERAPY OF LARYNX USING PALLADIUM 103 |
| ICD10_proc | D91BBCZ | LDR BRACHYTHERAPY OF LARYNX USING CALIFORNIUM 252 |
| ICD10_proc | D91BBYZ | LDR BRACHYTHERAPY OF LARYNX USING OTH ISOTOPE |
| ICD10_proc | D91BB7Z | LOW DOSE RATE (LDR) BRACHYTHERAPY OF LARYNX USING CESIUM 137 |
| ICD10_proc | D91BB8Z | LDR BRACHYTHERAPY OF LARYNX USING IRIDIUM 192 |
| ICD10_proc | D91BB9Z | LOW DOSE RATE (LDR) BRACHYTHERAPY OF LARYNX USING IODINE 125 |
| ICD10_proc | D91B9BZ | HDR BRACHYTHERAPY OF LARYNX USING PALLADIUM 103 |
| ICD10_proc | D91B9CZ | HDR BRACHYTHERAPY OF LARYNX USING CALIFORNIUM 252 |
| ICD10_proc | D91B9YZ | HDR BRACHYTHERAPY OF LARYNX USING OTH ISOTOPE |
| ICD10_proc | D91B97Z | HDR BRACHYTHERAPY OF LARYNX USING CESIUM 137 |
| ICD10_proc | D91B98Z | HDR BRACHYTHERAPY OF LARYNX USING IRIDIUM 192 |
| ICD10_proc | D91B99Z | HDR BRACHYTHERAPY OF LARYNX USING IODINE 125 |
| ICD10_proc | D91DBBZ | LDR BRACHYTHERAPY OF NASOPHARYNX USING PALLADIUM 103 |
| ICD10_proc | D91DBCZ | LDR BRACHYTHERAPY OF NASOPHARYNX USING CALIFORNIUM 252 |
| ICD10_proc | D91DBYZ | LDR BRACHYTHERAPY OF NASOPHARYNX USING OTH ISOTOPE |
| ICD10_proc | D91DB7Z | LDR BRACHYTHERAPY OF NASOPHARYNX USING CESIUM 137 |
| ICD10_proc | D91DB8Z | LDR BRACHYTHERAPY OF NASOPHARYNX USING IRIDIUM 192 |
| ICD10_proc | D91DB9Z | LDR BRACHYTHERAPY OF NASOPHARYNX USING IODINE 125 |
| ICD10_proc | D91D9BZ | HDR BRACHYTHERAPY OF NASOPHARYNX USING PALLADIUM 103 |
| ICD10_proc | D91D9CZ | HDR BRACHYTHERAPY OF NASOPHARYNX USING CALIFORNIUM 252 |
| ICD10_proc | D91D9YZ | HDR BRACHYTHERAPY OF NASOPHARYNX USING OTH ISOTOPE |
| ICD10_proc | D91D97Z | HDR BRACHYTHERAPY OF NASOPHARYNX USING CESIUM 137 |
| ICD10_proc | D91D98Z | HDR BRACHYTHERAPY OF NASOPHARYNX USING IRIDIUM 192 |
| ICD10_proc | D91D99Z | HDR BRACHYTHERAPY OF NASOPHARYNX USING IODINE 125 |
| ICD10_proc | D91FBBZ | LDR BRACHYTHERAPY OF OROPHARYNX USING PALLADIUM 103 |
| ICD10_proc | D91FBCZ | LDR BRACHYTHERAPY OF OROPHARYNX USING CALIFORNIUM 252 |
| ICD10_proc | D91FBYZ | LDR BRACHYTHERAPY OF OROPHARYNX USING OTH ISOTOPE |
| ICD10_proc | D91FB7Z | LDR BRACHYTHERAPY OF OROPHARYNX USING CESIUM 137 |
| ICD10_proc | D91FB8Z | LDR BRACHYTHERAPY OF OROPHARYNX USING IRIDIUM 192 |
| ICD10_proc | D91FB9Z | LDR BRACHYTHERAPY OF OROPHARYNX USING IODINE 125 |
| ICD10_proc | D91F9BZ | HDR BRACHYTHERAPY OF OROPHARYNX USING PALLADIUM 103 |
| ICD10_proc | D91F9CZ | HDR BRACHYTHERAPY OF OROPHARYNX USING CALIFORNIUM 252 |
| ICD10_proc | D91F9YZ | HDR BRACHYTHERAPY OF OROPHARYNX USING OTH ISOTOPE |
| ICD10_proc | D91F97Z | HDR BRACHYTHERAPY OF OROPHARYNX USING CESIUM 137 |
| ICD10_proc | D91F98Z | HDR BRACHYTHERAPY OF OROPHARYNX USING IRIDIUM 192 |
| ICD10_proc | D91F99Z | HDR BRACHYTHERAPY OF OROPHARYNX USING IODINE 125 |
| ICD10_proc | D910BBZ | LOW DOSE RATE (LDR) BRACHYTHERAPY OF EAR USING PALLADIUM 103 |
| ICD10_proc | D910BCZ | LDR BRACHYTHERAPY OF EAR USING CALIFORNIUM 252 |
| ICD10_proc | D910BYZ | LOW DOSE RATE (LDR) BRACHYTHERAPY OF EAR USING OTHER ISOTOPE |
| ICD10_proc | D910B7Z | LOW DOSE RATE (LDR) BRACHYTHERAPY OF EAR USING CESIUM 137 |
| ICD10_proc | D910B8Z | LOW DOSE RATE (LDR) BRACHYTHERAPY OF EAR USING IRIDIUM 192 |
| ICD10_proc | D910B9Z | LOW DOSE RATE (LDR) BRACHYTHERAPY OF EAR USING IODINE 125 |
| ICD10_proc | D9109BZ | HDR BRACHYTHERAPY OF EAR USING PALLADIUM 103 |
| ICD10_proc | D9109CZ | HDR BRACHYTHERAPY OF EAR USING CALIFORNIUM 252 |
| ICD10_proc | D9109YZ | HIGH DOSE RATE (HDR) BRACHYTHERAPY OF EAR USING OTH ISOTOPE |
| ICD10_proc | D91097Z | HIGH DOSE RATE (HDR) BRACHYTHERAPY OF EAR USING CESIUM 137 |
| ICD10_proc | D91098Z | HIGH DOSE RATE (HDR) BRACHYTHERAPY OF EAR USING IRIDIUM 192 |
| ICD10_proc | D91099Z | HIGH DOSE RATE (HDR) BRACHYTHERAPY OF EAR USING IODINE 125 |
| ICD10_proc | D911BBZ | LDR BRACHYTHERAPY OF NOSE USING PALLADIUM 103 |
| ICD10_proc | D911BCZ | LDR BRACHYTHERAPY OF NOSE USING CALIFORNIUM 252 |
| ICD10_proc | D911BYZ | LOW DOSE RATE (LDR) BRACHYTHERAPY OF NOSE USING OTH ISOTOPE |
| ICD10_proc | D911B7Z | LOW DOSE RATE (LDR) BRACHYTHERAPY OF NOSE USING CESIUM 137 |
| ICD10_proc | D911B8Z | LOW DOSE RATE (LDR) BRACHYTHERAPY OF NOSE USING IRIDIUM 192 |
| ICD10_proc | D911B9Z | LOW DOSE RATE (LDR) BRACHYTHERAPY OF NOSE USING IODINE 125 |
| ICD10_proc | D9119BZ | HDR BRACHYTHERAPY OF NOSE USING PALLADIUM 103 |
| ICD10_proc | D9119CZ | HDR BRACHYTHERAPY OF NOSE USING CALIFORNIUM 252 |
| ICD10_proc | D9119YZ | HIGH DOSE RATE (HDR) BRACHYTHERAPY OF NOSE USING OTH ISOTOPE |
| ICD10_proc | D91197Z | HIGH DOSE RATE (HDR) BRACHYTHERAPY OF NOSE USING CESIUM 137 |
| ICD10_proc | D91198Z | HIGH DOSE RATE (HDR) BRACHYTHERAPY OF NOSE USING IRIDIUM 192 |
| ICD10_proc | D91199Z | HIGH DOSE RATE (HDR) BRACHYTHERAPY OF NOSE USING IODINE 125 |
| ICD10_proc | D913BBZ | LDR BRACHYTHERAPY OF HYPOPHARYNX USING PALLADIUM 103 |
| ICD10_proc | D913BCZ | LDR BRACHYTHERAPY OF HYPOPHARYNX USING CALIFORNIUM 252 |
| ICD10_proc | D913BYZ | LDR BRACHYTHERAPY OF HYPOPHARYNX USING OTH ISOTOPE |
| ICD10_proc | D913B7Z | LDR BRACHYTHERAPY OF HYPOPHARYNX USING CESIUM 137 |
| ICD10_proc | D913B8Z | LDR BRACHYTHERAPY OF HYPOPHARYNX USING IRIDIUM 192 |
| ICD10_proc | D913B9Z | LDR BRACHYTHERAPY OF HYPOPHARYNX USING IODINE 125 |
| ICD10_proc | D9139BZ | HDR BRACHYTHERAPY OF HYPOPHARYNX USING PALLADIUM 103 |
| ICD10_proc | D9139CZ | HDR BRACHYTHERAPY OF HYPOPHARYNX USING CALIFORNIUM 252 |
| ICD10_proc | D9139YZ | HDR BRACHYTHERAPY OF HYPOPHARYNX USING OTH ISOTOPE |
| ICD10_proc | D91397Z | HDR BRACHYTHERAPY OF HYPOPHARYNX USING CESIUM 137 |
| ICD10_proc | D91398Z | HDR BRACHYTHERAPY OF HYPOPHARYNX USING IRIDIUM 192 |
| ICD10_proc | D91399Z | HDR BRACHYTHERAPY OF HYPOPHARYNX USING IODINE 125 |
| ICD10_proc | D914BBZ | LDR BRACHYTHERAPY OF MOUTH USING PALLADIUM 103 |
| ICD10_proc | D914BCZ | LDR BRACHYTHERAPY OF MOUTH USING CALIFORNIUM 252 |
| ICD10_proc | D914BYZ | LOW DOSE RATE (LDR) BRACHYTHERAPY OF MOUTH USING OTH ISOTOPE |
| ICD10_proc | D914B7Z | LOW DOSE RATE (LDR) BRACHYTHERAPY OF MOUTH USING CESIUM 137 |
| ICD10_proc | D914B8Z | LOW DOSE RATE (LDR) BRACHYTHERAPY OF MOUTH USING IRIDIUM 192 |
| ICD10_proc | D914B9Z | LOW DOSE RATE (LDR) BRACHYTHERAPY OF MOUTH USING IODINE 125 |
| ICD10_proc | D9149BZ | HDR BRACHYTHERAPY OF MOUTH USING PALLADIUM 103 |
| ICD10_proc | D9149CZ | HDR BRACHYTHERAPY OF MOUTH USING CALIFORNIUM 252 |
| ICD10_proc | D9149YZ | HDR BRACHYTHERAPY OF MOUTH USING OTH ISOTOPE |
| ICD10_proc | D91497Z | HIGH DOSE RATE (HDR) BRACHYTHERAPY OF MOUTH USING CESIUM 137 |
| ICD10_proc | D91498Z | HDR BRACHYTHERAPY OF MOUTH USING IRIDIUM 192 |
| ICD10_proc | D91499Z | HIGH DOSE RATE (HDR) BRACHYTHERAPY OF MOUTH USING IODINE 125 |
| ICD10_proc | D915BBZ | LDR BRACHYTHERAPY OF TONGUE USING PALLADIUM 103 |
| ICD10_proc | D915BCZ | LDR BRACHYTHERAPY OF TONGUE USING CALIFORNIUM 252 |
| ICD10_proc | D915BYZ | LDR BRACHYTHERAPY OF TONGUE USING OTH ISOTOPE |
| ICD10_proc | D915B7Z | LOW DOSE RATE (LDR) BRACHYTHERAPY OF TONGUE USING CESIUM 137 |
| ICD10_proc | D915B8Z | LDR BRACHYTHERAPY OF TONGUE USING IRIDIUM 192 |
| ICD10_proc | D915B9Z | LOW DOSE RATE (LDR) BRACHYTHERAPY OF TONGUE USING IODINE 125 |
| ICD10_proc | D9159BZ | HDR BRACHYTHERAPY OF TONGUE USING PALLADIUM 103 |
| ICD10_proc | D9159CZ | HDR BRACHYTHERAPY OF TONGUE USING CALIFORNIUM 252 |
| ICD10_proc | D9159YZ | HDR BRACHYTHERAPY OF TONGUE USING OTH ISOTOPE |
| ICD10_proc | D91597Z | HDR BRACHYTHERAPY OF TONGUE USING CESIUM 137 |
| ICD10_proc | D91598Z | HDR BRACHYTHERAPY OF TONGUE USING IRIDIUM 192 |
| ICD10_proc | D91599Z | HDR BRACHYTHERAPY OF TONGUE USING IODINE 125 |
| ICD10_proc | D916BBZ | LDR BRACHYTHERAPY OF SALIVARY GLAND USING PALLADIUM 103 |
| ICD10_proc | D916BCZ | LDR BRACHYTHERAPY OF SALIVARY GLAND USING CALIFORNIUM 252 |
| ICD10_proc | D916BYZ | LDR BRACHYTHERAPY OF SALIVARY GLAND USING OTH ISOTOPE |
| ICD10_proc | D916B7Z | LDR BRACHYTHERAPY OF SALIVARY GLAND USING CESIUM 137 |
| ICD10_proc | D916B8Z | LDR BRACHYTHERAPY OF SALIVARY GLAND USING IRIDIUM 192 |
| ICD10_proc | D916B9Z | LDR BRACHYTHERAPY OF SALIVARY GLAND USING IODINE 125 |
| ICD10_proc | D9169BZ | HDR BRACHYTHERAPY OF SALIVARY GLAND USING PALLADIUM 103 |
| ICD10_proc | D9169CZ | HDR BRACHYTHERAPY OF SALIVARY GLAND USING CALIFORNIUM 252 |
| ICD10_proc | D9169YZ | HDR BRACHYTHERAPY OF SALIVARY GLAND USING OTH ISOTOPE |
| ICD10_proc | D91697Z | HDR BRACHYTHERAPY OF SALIVARY GLAND USING CESIUM 137 |
| ICD10_proc | D91698Z | HDR BRACHYTHERAPY OF SALIVARY GLAND USING IRIDIUM 192 |
| ICD10_proc | D91699Z | HDR BRACHYTHERAPY OF SALIVARY GLAND USING IODINE 125 |
| ICD10_proc | D917BBZ | LDR BRACHYTHERAPY OF SINUSES USING PALLADIUM 103 |
| ICD10_proc | D917BCZ | LDR BRACHYTHERAPY OF SINUSES USING CALIFORNIUM 252 |
| ICD10_proc | D917BYZ | LDR BRACHYTHERAPY OF SINUSES USING OTH ISOTOPE |
| ICD10_proc | D917B7Z | LDR BRACHYTHERAPY OF SINUSES USING CESIUM 137 |
| ICD10_proc | D917B8Z | LDR BRACHYTHERAPY OF SINUSES USING IRIDIUM 192 |
| ICD10_proc | D917B9Z | LDR BRACHYTHERAPY OF SINUSES USING IODINE 125 |
| ICD10_proc | D9179BZ | HDR BRACHYTHERAPY OF SINUSES USING PALLADIUM 103 |
| ICD10_proc | D9179CZ | HDR BRACHYTHERAPY OF SINUSES USING CALIFORNIUM 252 |
| ICD10_proc | D9179YZ | HDR BRACHYTHERAPY OF SINUSES USING OTH ISOTOPE |
| ICD10_proc | D91797Z | HDR BRACHYTHERAPY OF SINUSES USING CESIUM 137 |
| ICD10_proc | D91798Z | HDR BRACHYTHERAPY OF SINUSES USING IRIDIUM 192 |
| ICD10_proc | D91799Z | HDR BRACHYTHERAPY OF SINUSES USING IODINE 125 |
| ICD10_proc | D918BBZ | LDR BRACHYTHERAPY OF HARD PALATE USING PALLADIUM 103 |
| ICD10_proc | D918BCZ | LDR BRACHYTHERAPY OF HARD PALATE USING CALIFORNIUM 252 |
| ICD10_proc | D918BYZ | LDR BRACHYTHERAPY OF HARD PALATE USING OTH ISOTOPE |
| ICD10_proc | D918B7Z | LDR BRACHYTHERAPY OF HARD PALATE USING CESIUM 137 |
| ICD10_proc | D918B8Z | LDR BRACHYTHERAPY OF HARD PALATE USING IRIDIUM 192 |
| ICD10_proc | D918B9Z | LDR BRACHYTHERAPY OF HARD PALATE USING IODINE 125 |
| ICD10_proc | D9189BZ | HDR BRACHYTHERAPY OF HARD PALATE USING PALLADIUM 103 |
| ICD10_proc | D9189CZ | HDR BRACHYTHERAPY OF HARD PALATE USING CALIFORNIUM 252 |
| ICD10_proc | D9189YZ | HDR BRACHYTHERAPY OF HARD PALATE USING OTH ISOTOPE |
| ICD10_proc | D91897Z | HDR BRACHYTHERAPY OF HARD PALATE USING CESIUM 137 |
| ICD10_proc | D91898Z | HDR BRACHYTHERAPY OF HARD PALATE USING IRIDIUM 192 |
| ICD10_proc | D91899Z | HDR BRACHYTHERAPY OF HARD PALATE USING IODINE 125 |
| ICD10_proc | D919BBZ | LDR BRACHYTHERAPY OF SOFT PALATE USING PALLADIUM 103 |
| ICD10_proc | D919BCZ | LDR BRACHYTHERAPY OF SOFT PALATE USING CALIFORNIUM 252 |
| ICD10_proc | D919BYZ | LDR BRACHYTHERAPY OF SOFT PALATE USING OTH ISOTOPE |
| ICD10_proc | D919B7Z | LDR BRACHYTHERAPY OF SOFT PALATE USING CESIUM 137 |
| ICD10_proc | D919B8Z | LDR BRACHYTHERAPY OF SOFT PALATE USING IRIDIUM 192 |
| ICD10_proc | D919B9Z | LDR BRACHYTHERAPY OF SOFT PALATE USING IODINE 125 |
| ICD10_proc | D9199BZ | HDR BRACHYTHERAPY OF SOFT PALATE USING PALLADIUM 103 |
| ICD10_proc | D9199CZ | HDR BRACHYTHERAPY OF SOFT PALATE USING CALIFORNIUM 252 |
| ICD10_proc | D9199YZ | HDR BRACHYTHERAPY OF SOFT PALATE USING OTH ISOTOPE |
| ICD10_proc | D91997Z | HDR BRACHYTHERAPY OF SOFT PALATE USING CESIUM 137 |
| ICD10_proc | D91998Z | HDR BRACHYTHERAPY OF SOFT PALATE USING IRIDIUM 192 |
| ICD10_proc | D91999Z | HDR BRACHYTHERAPY OF SOFT PALATE USING IODINE 125 |
| ICD10_proc | DB001ZZ | BEAM RADIATION OF TRACHEA USING PHOTONS 1 - 10 MEV |
| ICD10_proc | DB002ZZ | BEAM RADIATION OF TRACHEA USING PHOTONS >10 MEV |
| ICD10_proc | DB011ZZ | BEAM RADIATION OF BRONCHUS USING PHOTONS 1 - 10 MEV |
| ICD10_proc | DB012ZZ | BEAM RADIATION OF BRONCHUS USING PHOTONS >10 MEV |
| ICD10_proc | DB021ZZ | BEAM RADIATION OF LUNG USING PHOTONS 1 - 10 MEV |
| ICD10_proc | DB022ZZ | BEAM RADIATION OF LUNG USING PHOTONS >10 MEV |
| ICD10_proc | DB051ZZ | BEAM RADIATION OF PLEURA USING PHOTONS 1 - 10 MEV |
| ICD10_proc | DB052ZZ | BEAM RADIATION OF PLEURA USING PHOTONS >10 MEV |
| ICD10_proc | DB061ZZ | BEAM RADIATION OF MEDIASTINUM USING PHOTONS 1 - 10 MEV |
| ICD10_proc | DB062ZZ | BEAM RADIATION OF MEDIASTINUM USING PHOTONS >10 MEV |
| ICD10_proc | DB071ZZ | BEAM RADIATION OF CHEST WALL USING PHOTONS 1 - 10 MEV |
| ICD10_proc | DB072ZZ | BEAM RADIATION OF CHEST WALL USING PHOTONS >10 MEV |
| ICD10_proc | DB081ZZ | BEAM RADIATION OF DIAPHRAGM USING PHOTONS 1 - 10 MEV |
| ICD10_proc | DB082ZZ | BEAM RADIATION OF DIAPHRAGM USING PHOTONS >10 MEV |
| ICD10_proc | DD000ZZ | BEAM RADIATION OF ESOPHAGUS USING PHOTONS <1 MEV |
| ICD10_proc | DD001ZZ | BEAM RADIATION OF ESOPHAGUS USING PHOTONS 1 - 10 MEV |
| ICD10_proc | DD002ZZ | BEAM RADIATION OF ESOPHAGUS USING PHOTONS >10 MEV |
| ICD10_proc | DD010ZZ | BEAM RADIATION OF STOMACH USING PHOTONS <1 MEV |
| ICD10_proc | DD011ZZ | BEAM RADIATION OF STOMACH USING PHOTONS 1 - 10 MEV |
| ICD10_proc | DD012ZZ | BEAM RADIATION OF STOMACH USING PHOTONS >10 MEV |
| ICD10_proc | DD020ZZ | BEAM RADIATION OF DUODENUM USING PHOTONS <1 MEV |
| ICD10_proc | DD021ZZ | BEAM RADIATION OF DUODENUM USING PHOTONS 1 - 10 MEV |
| ICD10_proc | DD022ZZ | BEAM RADIATION OF DUODENUM USING PHOTONS >10 MEV |
| ICD10_proc | DD031ZZ | BEAM RADIATION OF JEJUNUM USING PHOTONS 1 - 10 MEV |
| ICD10_proc | DD032ZZ | BEAM RADIATION OF JEJUNUM USING PHOTONS >10 MEV |
| ICD10_proc | DD041ZZ | BEAM RADIATION OF ILEUM USING PHOTONS 1 - 10 MEV |
| ICD10_proc | DD042ZZ | BEAM RADIATION OF ILEUM USING PHOTONS >10 MEV |
| ICD10_proc | DD051ZZ | BEAM RADIATION OF COLON USING PHOTONS 1 - 10 MEV |
| ICD10_proc | DD052ZZ | BEAM RADIATION OF COLON USING PHOTONS >10 MEV |
| ICD10_proc | DD071ZZ | BEAM RADIATION OF RECTUM USING PHOTONS 1 - 10 MEV |
| ICD10_proc | DD072ZZ | BEAM RADIATION OF RECTUM USING PHOTONS >10 MEV |
| ICD10_proc | DF001ZZ | BEAM RADIATION OF LIVER USING PHOTONS 1 - 10 MEV |
| ICD10_proc | DF002ZZ | BEAM RADIATION OF LIVER USING PHOTONS >10 MEV |
| ICD10_proc | DF011ZZ | BEAM RADIATION OF GALLBLADDER USING PHOTONS 1 - 10 MEV |
| ICD10_proc | DF012ZZ | BEAM RADIATION OF GALLBLADDER USING PHOTONS >10 MEV |
| ICD10_proc | DF021ZZ | BEAM RADIATION OF BILE DUCTS USING PHOTONS 1 - 10 MEV |
| ICD10_proc | DF022ZZ | BEAM RADIATION OF BILE DUCTS USING PHOTONS >10 MEV |
| ICD10_proc | DF031ZZ | BEAM RADIATION OF PANCREAS USING PHOTONS 1 - 10 MEV |
| ICD10_proc | DF032ZZ | BEAM RADIATION OF PANCREAS USING PHOTONS >10 MEV |
| ICD10_proc | DG001ZZ | BEAM RADIATION OF PITUITARY GLAND USING PHOTONS 1 - 10 MEV |
| ICD10_proc | DG002ZZ | BEAM RADIATION OF PITUITARY GLAND USING PHOTONS >10 MEV |
| ICD10_proc | DG011ZZ | BEAM RADIATION OF PINEAL BODY USING PHOTONS 1 - 10 MEV |
| ICD10_proc | DG012ZZ | BEAM RADIATION OF PINEAL BODY USING PHOTONS >10 MEV |
| ICD10_proc | DG021ZZ | BEAM RADIATION OF ADRENAL GLANDS USING PHOTONS 1 - 10 MEV |
| ICD10_proc | DG022ZZ | BEAM RADIATION OF ADRENAL GLANDS USING PHOTONS >10 MEV |
| ICD10_proc | DG041ZZ | BEAM RADIATION PARATHYROID GLANDS W PHOTONS 1 - 10 MEV |
| ICD10_proc | DG042ZZ | BEAM RADIATION OF PARATHYROID GLANDS USING PHOTONS >10 MEV |
| ICD10_proc | DG051ZZ | BEAM RADIATION OF THYROID USING PHOTONS 1 - 10 MEV |
| ICD10_proc | DG052ZZ | BEAM RADIATION OF THYROID USING PHOTONS >10 MEV |
| ICD10_proc | DH0B1ZZ | BEAM RADIATION OF LEG SKIN USING PHOTONS 1 - 10 MEV |
| ICD10_proc | DH0B2ZZ | BEAM RADIATION OF LEG SKIN USING PHOTONS >10 MEV |
| ICD10_proc | DH021ZZ | BEAM RADIATION OF FACE SKIN USING PHOTONS 1 - 10 MEV |
| ICD10_proc | DH022ZZ | BEAM RADIATION OF FACE SKIN USING PHOTONS >10 MEV |
| ICD10_proc | DH031ZZ | BEAM RADIATION OF NECK SKIN USING PHOTONS 1 - 10 MEV |
| ICD10_proc | DH032ZZ | BEAM RADIATION OF NECK SKIN USING PHOTONS >10 MEV |
| ICD10_proc | DH041ZZ | BEAM RADIATION OF ARM SKIN USING PHOTONS 1 - 10 MEV |
| ICD10_proc | DH042ZZ | BEAM RADIATION OF ARM SKIN USING PHOTONS >10 MEV |
| ICD10_proc | DH061ZZ | BEAM RADIATION OF CHEST SKIN USING PHOTONS 1 - 10 MEV |
| ICD10_proc | DH062ZZ | BEAM RADIATION OF CHEST SKIN USING PHOTONS >10 MEV |
| ICD10_proc | DH071ZZ | BEAM RADIATION OF BACK SKIN USING PHOTONS 1 - 10 MEV |
| ICD10_proc | DH072ZZ | BEAM RADIATION OF BACK SKIN USING PHOTONS >10 MEV |
| ICD10_proc | DH081ZZ | BEAM RADIATION OF ABDOMEN SKIN USING PHOTONS 1 - 10 MEV |
| ICD10_proc | DH082ZZ | BEAM RADIATION OF ABDOMEN SKIN USING PHOTONS >10 MEV |
| ICD10_proc | DH091ZZ | BEAM RADIATION OF BUTTOCK SKIN USING PHOTONS 1 - 10 MEV |
| ICD10_proc | DH092ZZ | BEAM RADIATION OF BUTTOCK SKIN USING PHOTONS >10 MEV |
| ICD10_proc | DM001ZZ | BEAM RADIATION OF LEFT BREAST USING PHOTONS 1 - 10 MEV |
| ICD10_proc | DM002ZZ | BEAM RADIATION OF LEFT BREAST USING PHOTONS >10 MEV |
| ICD10_proc | DM010ZZ | BEAM RADIATION OF RIGHT BREAST USING PHOTONS <1 MEV |
| ICD10_proc | DM011ZZ | BEAM RADIATION OF RIGHT BREAST USING PHOTONS 1 - 10 MEV |
| ICD10_proc | DM012ZZ | BEAM RADIATION OF RIGHT BREAST USING PHOTONS >10 MEV |
| ICD10_proc | DP0B0ZZ | BEAM RADIATION OF TIBIA/FIBULA USING PHOTONS <1 MEV |
| ICD10_proc | DP0B1ZZ | BEAM RADIATION OF TIBIA/FIBULA USING PHOTONS 1 - 10 MEV |
| ICD10_proc | DP0B2ZZ | BEAM RADIATION OF TIBIA/FIBULA USING PHOTONS >10 MEV |
| ICD10_proc | DP0C0ZZ | BEAM RADIATION OF OTHER BONE USING PHOTONS <1 MEV |
| ICD10_proc | DP0C1ZZ | BEAM RADIATION OF OTHER BONE USING PHOTONS 1 - 10 MEV |
| ICD10_proc | DP0C2ZZ | BEAM RADIATION OF OTHER BONE USING PHOTONS >10 MEV |
| ICD10_proc | DP000ZZ | BEAM RADIATION OF SKULL USING PHOTONS <1 MEV |
| ICD10_proc | DP001ZZ | BEAM RADIATION OF SKULL USING PHOTONS 1 - 10 MEV |
| ICD10_proc | DP002ZZ | BEAM RADIATION OF SKULL USING PHOTONS >10 MEV |
| ICD10_proc | DP021ZZ | BEAM RADIATION OF MAXILLA USING PHOTONS 1 - 10 MEV |
| ICD10_proc | DP022ZZ | BEAM RADIATION OF MAXILLA USING PHOTONS >10 MEV |
| ICD10_proc | DP031ZZ | BEAM RADIATION OF MANDIBLE USING PHOTONS 1 - 10 MEV |
| ICD10_proc | DP032ZZ | BEAM RADIATION OF MANDIBLE USING PHOTONS >10 MEV |
| ICD10_proc | DP041ZZ | BEAM RADIATION OF STERNUM USING PHOTONS 1 - 10 MEV |
| ICD10_proc | DP042ZZ | BEAM RADIATION OF STERNUM USING PHOTONS >10 MEV |
| ICD10_proc | DP050ZZ | BEAM RADIATION OF RIB(S) USING PHOTONS <1 MEV |
| ICD10_proc | DP051ZZ | BEAM RADIATION OF RIB(S) USING PHOTONS 1 - 10 MEV |
| ICD10_proc | DP052ZZ | BEAM RADIATION OF RIB(S) USING PHOTONS >10 MEV |
| ICD10_proc | DP061ZZ | BEAM RADIATION OF HUMERUS USING PHOTONS 1 - 10 MEV |
| ICD10_proc | DP062ZZ | BEAM RADIATION OF HUMERUS USING PHOTONS >10 MEV |
| ICD10_proc | DP071ZZ | BEAM RADIATION OF RADIUS/ULNA USING PHOTONS 1 - 10 MEV |
| ICD10_proc | DP072ZZ | BEAM RADIATION OF RADIUS/ULNA USING PHOTONS >10 MEV |
| ICD10_proc | DP080ZZ | BEAM RADIATION OF PELVIC BONES USING PHOTONS <1 MEV |
| ICD10_proc | DP081ZZ | BEAM RADIATION OF PELVIC BONES USING PHOTONS 1 - 10 MEV |
| ICD10_proc | DP082ZZ | BEAM RADIATION OF PELVIC BONES USING PHOTONS >10 MEV |
| ICD10_proc | DP090ZZ | BEAM RADIATION OF FEMUR USING PHOTONS <1 MEV |
| ICD10_proc | DP091ZZ | BEAM RADIATION OF FEMUR USING PHOTONS 1 - 10 MEV |
| ICD10_proc | DP092ZZ | BEAM RADIATION OF FEMUR USING PHOTONS >10 MEV |
| ICD10_proc | DT000ZZ | BEAM RADIATION OF KIDNEY USING PHOTONS <1 MEV |
| ICD10_proc | DT001ZZ | BEAM RADIATION OF KIDNEY USING PHOTONS 1 - 10 MEV |
| ICD10_proc | DT002ZZ | BEAM RADIATION OF KIDNEY USING PHOTONS >10 MEV |
| ICD10_proc | DT010ZZ | BEAM RADIATION OF URETER USING PHOTONS <1 MEV |
| ICD10_proc | DT011ZZ | BEAM RADIATION OF URETER USING PHOTONS 1 - 10 MEV |
| ICD10_proc | DT012ZZ | BEAM RADIATION OF URETER USING PHOTONS >10 MEV |
| ICD10_proc | DT020ZZ | BEAM RADIATION OF BLADDER USING PHOTONS <1 MEV |
| ICD10_proc | DT021ZZ | BEAM RADIATION OF BLADDER USING PHOTONS 1 - 10 MEV |
| ICD10_proc | DT022ZZ | BEAM RADIATION OF BLADDER USING PHOTONS >10 MEV |
| ICD10_proc | DT030ZZ | BEAM RADIATION OF URETHRA USING PHOTONS <1 MEV |
| ICD10_proc | DT031ZZ | BEAM RADIATION OF URETHRA USING PHOTONS 1 - 10 MEV |
| ICD10_proc | DT032ZZ | BEAM RADIATION OF URETHRA USING PHOTONS >10 MEV |
| ICD10_proc | DU000ZZ | BEAM RADIATION OF OVARY USING PHOTONS <1 MEV |
| ICD10_proc | DU001ZZ | BEAM RADIATION OF OVARY USING PHOTONS 1 - 10 MEV |
| ICD10_proc | DU002ZZ | BEAM RADIATION OF OVARY USING PHOTONS >10 MEV |
| ICD10_proc | DU010ZZ | BEAM RADIATION OF CERVIX USING PHOTONS <1 MEV |
| ICD10_proc | DU011ZZ | BEAM RADIATION OF CERVIX USING PHOTONS 1 - 10 MEV |
| ICD10_proc | DU012ZZ | BEAM RADIATION OF CERVIX USING PHOTONS >10 MEV |
| ICD10_proc | DU020ZZ | BEAM RADIATION OF UTERUS USING PHOTONS <1 MEV |
| ICD10_proc | DU021ZZ | BEAM RADIATION OF UTERUS USING PHOTONS 1 - 10 MEV |
| ICD10_proc | DU022ZZ | BEAM RADIATION OF UTERUS USING PHOTONS >10 MEV |
| ICD10_proc | DV000ZZ | BEAM RADIATION OF PROSTATE USING PHOTONS <1 MEV |
| ICD10_proc | DV001ZZ | BEAM RADIATION OF PROSTATE USING PHOTONS 1 - 10 MEV |
| ICD10_proc | DV002ZZ | BEAM RADIATION OF PROSTATE USING PHOTONS >10 MEV |
| ICD10_proc | DV010ZZ | BEAM RADIATION OF TESTIS USING PHOTONS <1 MEV |
| ICD10_proc | DV011ZZ | BEAM RADIATION OF TESTIS USING PHOTONS 1 - 10 MEV |
| ICD10_proc | DV012ZZ | BEAM RADIATION OF TESTIS USING PHOTONS >10 MEV |
| ICD10_proc | DW010ZZ | BEAM RADIATION OF HEAD AND NECK USING PHOTONS <1 MEV |
| ICD10_proc | DW011ZZ | BEAM RADIATION OF HEAD AND NECK USING PHOTONS 1 - 10 MEV |
| ICD10_proc | DW012ZZ | BEAM RADIATION OF HEAD AND NECK USING PHOTONS >10 MEV |
| ICD10_proc | DW020ZZ | BEAM RADIATION OF CHEST USING PHOTONS <1 MEV |
| ICD10_proc | DW021ZZ | BEAM RADIATION OF CHEST USING PHOTONS 1 - 10 MEV |
| ICD10_proc | DW022ZZ | BEAM RADIATION OF CHEST USING PHOTONS >10 MEV |
| ICD10_proc | DW030ZZ | BEAM RADIATION OF ABDOMEN USING PHOTONS <1 MEV |
| ICD10_proc | DW031ZZ | BEAM RADIATION OF ABDOMEN USING PHOTONS 1 - 10 MEV |
| ICD10_proc | DW032ZZ | BEAM RADIATION OF ABDOMEN USING PHOTONS >10 MEV |
| ICD10_proc | DW040ZZ | BEAM RADIATION OF HEMIBODY USING PHOTONS <1 MEV |
| ICD10_proc | DW041ZZ | BEAM RADIATION OF HEMIBODY USING PHOTONS 1 - 10 MEV |
| ICD10_proc | DW042ZZ | BEAM RADIATION OF HEMIBODY USING PHOTONS >10 MEV |
| ICD10_proc | DW050ZZ | BEAM RADIATION OF WHOLE BODY USING PHOTONS <1 MEV |
| ICD10_proc | DW051ZZ | BEAM RADIATION OF WHOLE BODY USING PHOTONS 1 - 10 MEV |
| ICD10_proc | DW052ZZ | BEAM RADIATION OF WHOLE BODY USING PHOTONS >10 MEV |
| ICD10_proc | DW060ZZ | BEAM RADIATION OF PELVIC REGION USING PHOTONS <1 MEV |
| ICD10_proc | DW061ZZ | BEAM RADIATION OF PELVIC REGION USING PHOTONS 1 - 10 MEV |
| ICD10_proc | DW062ZZ | BEAM RADIATION OF PELVIC REGION USING PHOTONS >10 MEV |
| ICD10_proc | D0000ZZ | BEAM RADIATION OF BRAIN USING PHOTONS <1 MEV |
| ICD10_proc | D0001ZZ | BEAM RADIATION OF BRAIN USING PHOTONS 1 - 10 MEV |
| ICD10_proc | D0002ZZ | BEAM RADIATION OF BRAIN USING PHOTONS >10 MEV |
| ICD10_proc | D0010ZZ | BEAM RADIATION OF BRAIN STEM USING PHOTONS <1 MEV |
| ICD10_proc | D0011ZZ | BEAM RADIATION OF BRAIN STEM USING PHOTONS 1 - 10 MEV |
| ICD10_proc | D0012ZZ | BEAM RADIATION OF BRAIN STEM USING PHOTONS >10 MEV |
| ICD10_proc | D0060ZZ | BEAM RADIATION OF SPINAL CORD USING PHOTONS <1 MEV |
| ICD10_proc | D0061ZZ | BEAM RADIATION OF SPINAL CORD USING PHOTONS 1 - 10 MEV |
| ICD10_proc | D0062ZZ | BEAM RADIATION OF SPINAL CORD USING PHOTONS >10 MEV |
| ICD10_proc | D0071ZZ | BEAM RADIATION OF PERIPHERAL NERVE USING PHOTONS 1 - 10 MEV |
| ICD10_proc | D0072ZZ | BEAM RADIATION OF PERIPHERAL NERVE USING PHOTONS >10 MEV |
| ICD10_proc | D7001ZZ | BEAM RADIATION OF BONE MARROW USING PHOTONS 1 - 10 MEV |
| ICD10_proc | D7002ZZ | BEAM RADIATION OF BONE MARROW USING PHOTONS >10 MEV |
| ICD10_proc | D7011ZZ | BEAM RADIATION OF THYMUS USING PHOTONS 1 - 10 MEV |
| ICD10_proc | D7012ZZ | BEAM RADIATION OF THYMUS USING PHOTONS >10 MEV |
| ICD10_proc | D7021ZZ | BEAM RADIATION OF SPLEEN USING PHOTONS 1 - 10 MEV |
| ICD10_proc | D7022ZZ | BEAM RADIATION OF SPLEEN USING PHOTONS >10 MEV |
| ICD10_proc | D7031ZZ | BEAM RADIATION OF NECK LYMPHATICS USING PHOTONS 1 - 10 MEV |
| ICD10_proc | D7032ZZ | BEAM RADIATION OF NECK LYMPHATICS USING PHOTONS >10 MEV |
| ICD10_proc | D7041ZZ | BEAM RADIATION OF AXILLA LYMPH USING PHOTONS 1 - 10 MEV |
| ICD10_proc | D7042ZZ | BEAM RADIATION OF AXILLARY LYMPHATICS USING PHOTONS >10 MEV |
| ICD10_proc | D7051ZZ | BEAM RADIATION OF THORAX LYMPHATICS USING PHOTONS 1 - 10 MEV |
| ICD10_proc | D7052ZZ | BEAM RADIATION OF THORAX LYMPHATICS USING PHOTONS >10 MEV |
| ICD10_proc | D7061ZZ | BEAM RADIATION OF ABD LYMPH USING PHOTONS 1 - 10 MEV |
| ICD10_proc | D7062ZZ | BEAM RADIATION OF ABDOMEN LYMPHATICS USING PHOTONS >10 MEV |
| ICD10_proc | D7071ZZ | BEAM RADIATION OF PELVIS LYMPHATICS USING PHOTONS 1 - 10 MEV |
| ICD10_proc | D7072ZZ | BEAM RADIATION OF PELVIS LYMPHATICS USING PHOTONS >10 MEV |
| ICD10_proc | D7081ZZ | BEAM RADIATION OF INGUINAL LYMPH USING PHOTONS 1 - 10 MEV |
| ICD10_proc | D7082ZZ | BEAM RADIATION OF INGUINAL LYMPHATICS USING PHOTONS >10 MEV |
| ICD10_proc | D8001ZZ | BEAM RADIATION OF EYE USING PHOTONS 1 - 10 MEV |
| ICD10_proc | D8002ZZ | BEAM RADIATION OF EYE USING PHOTONS >10 MEV |
| ICD10_proc | D90B1ZZ | BEAM RADIATION OF LARYNX USING PHOTONS 1 - 10 MEV |
| ICD10_proc | D90B2ZZ | BEAM RADIATION OF LARYNX USING PHOTONS >10 MEV |
| ICD10_proc | D90D1ZZ | BEAM RADIATION OF NASOPHARYNX USING PHOTONS 1 - 10 MEV |
| ICD10_proc | D90D2ZZ | BEAM RADIATION OF NASOPHARYNX USING PHOTONS >10 MEV |
| ICD10_proc | D90F1ZZ | BEAM RADIATION OF OROPHARYNX USING PHOTONS 1 - 10 MEV |
| ICD10_proc | D90F2ZZ | BEAM RADIATION OF OROPHARYNX USING PHOTONS >10 MEV |
| ICD10_proc | D9001ZZ | BEAM RADIATION OF EAR USING PHOTONS 1 - 10 MEV |
| ICD10_proc | D9002ZZ | BEAM RADIATION OF EAR USING PHOTONS >10 MEV |
| ICD10_proc | D9011ZZ | BEAM RADIATION OF NOSE USING PHOTONS 1 - 10 MEV |
| ICD10_proc | D9012ZZ | BEAM RADIATION OF NOSE USING PHOTONS >10 MEV |
| ICD10_proc | D9031ZZ | BEAM RADIATION OF HYPOPHARYNX USING PHOTONS 1 - 10 MEV |
| ICD10_proc | D9032ZZ | BEAM RADIATION OF HYPOPHARYNX USING PHOTONS >10 MEV |
| ICD10_proc | D9041ZZ | BEAM RADIATION OF MOUTH USING PHOTONS 1 - 10 MEV |
| ICD10_proc | D9042ZZ | BEAM RADIATION OF MOUTH USING PHOTONS >10 MEV |
| ICD10_proc | D9051ZZ | BEAM RADIATION OF TONGUE USING PHOTONS 1 - 10 MEV |
| ICD10_proc | D9052ZZ | BEAM RADIATION OF TONGUE USING PHOTONS >10 MEV |
| ICD10_proc | D9061ZZ | BEAM RADIATION OF SALIVARY GLANDS USING PHOTONS 1 - 10 MEV |
| ICD10_proc | D9062ZZ | BEAM RADIATION OF SALIVARY GLANDS USING PHOTONS >10 MEV |
| ICD10_proc | D9071ZZ | BEAM RADIATION OF SINUSES USING PHOTONS 1 - 10 MEV |
| ICD10_proc | D9072ZZ | BEAM RADIATION OF SINUSES USING PHOTONS >10 MEV |
| ICD10_proc | D9081ZZ | BEAM RADIATION OF HARD PALATE USING PHOTONS 1 - 10 MEV |
| ICD10_proc | D9082ZZ | BEAM RADIATION OF HARD PALATE USING PHOTONS >10 MEV |
| ICD10_proc | D9091ZZ | BEAM RADIATION OF SOFT PALATE USING PHOTONS 1 - 10 MEV |
| ICD10_proc | D9092ZZ | BEAM RADIATION OF SOFT PALATE USING PHOTONS >10 MEV |
| ICD10_proc | DB003ZZ | BEAM RADIATION OF TRACHEA USING ELECTRONS |
| ICD10_proc | DB013ZZ | BEAM RADIATION OF BRONCHUS USING ELECTRONS |
| ICD10_proc | DB023ZZ | BEAM RADIATION OF LUNG USING ELECTRONS |
| ICD10_proc | DB053ZZ | BEAM RADIATION OF PLEURA USING ELECTRONS |
| ICD10_proc | DB063ZZ | BEAM RADIATION OF MEDIASTINUM USING ELECTRONS |
| ICD10_proc | DB073ZZ | BEAM RADIATION OF CHEST WALL USING ELECTRONS |
| ICD10_proc | DB083ZZ | BEAM RADIATION OF DIAPHRAGM USING ELECTRONS |
| ICD10_proc | DD003ZZ | BEAM RADIATION OF ESOPHAGUS USING ELECTRONS |
| ICD10_proc | DD013ZZ | BEAM RADIATION OF STOMACH USING ELECTRONS |
| ICD10_proc | DD023ZZ | BEAM RADIATION OF DUODENUM USING ELECTRONS |
| ICD10_proc | DD033ZZ | BEAM RADIATION OF JEJUNUM USING ELECTRONS |
| ICD10_proc | DD043ZZ | BEAM RADIATION OF ILEUM USING ELECTRONS |
| ICD10_proc | DD053ZZ | BEAM RADIATION OF COLON USING ELECTRONS |
| ICD10_proc | DD073ZZ | BEAM RADIATION OF RECTUM USING ELECTRONS |
| ICD10_proc | DF003ZZ | BEAM RADIATION OF LIVER USING ELECTRONS |
| ICD10_proc | DF013ZZ | BEAM RADIATION OF GALLBLADDER USING ELECTRONS |
| ICD10_proc | DF023ZZ | BEAM RADIATION OF BILE DUCTS USING ELECTRONS |
| ICD10_proc | DF033ZZ | BEAM RADIATION OF PANCREAS USING ELECTRONS |
| ICD10_proc | DG003ZZ | BEAM RADIATION OF PITUITARY GLAND USING ELECTRONS |
| ICD10_proc | DG013ZZ | BEAM RADIATION OF PINEAL BODY USING ELECTRONS |
| ICD10_proc | DG023ZZ | BEAM RADIATION OF ADRENAL GLANDS USING ELECTRONS |
| ICD10_proc | DG043ZZ | BEAM RADIATION OF PARATHYROID GLANDS USING ELECTRONS |
| ICD10_proc | DG053ZZ | BEAM RADIATION OF THYROID USING ELECTRONS |
| ICD10_proc | DH0B3ZZ | BEAM RADIATION OF LEG SKIN USING ELECTRONS |
| ICD10_proc | DH023ZZ | BEAM RADIATION OF FACE SKIN USING ELECTRONS |
| ICD10_proc | DH033ZZ | BEAM RADIATION OF NECK SKIN USING ELECTRONS |
| ICD10_proc | DH043ZZ | BEAM RADIATION OF ARM SKIN USING ELECTRONS |
| ICD10_proc | DH063ZZ | BEAM RADIATION OF CHEST SKIN USING ELECTRONS |
| ICD10_proc | DH073ZZ | BEAM RADIATION OF BACK SKIN USING ELECTRONS |
| ICD10_proc | DH083ZZ | BEAM RADIATION OF ABDOMEN SKIN USING ELECTRONS |
| ICD10_proc | DH093ZZ | BEAM RADIATION OF BUTTOCK SKIN USING ELECTRONS |
| ICD10_proc | DM003ZZ | BEAM RADIATION OF LEFT BREAST USING ELECTRONS |
| ICD10_proc | DM013ZZ | BEAM RADIATION OF RIGHT BREAST USING ELECTRONS |
| ICD10_proc | DP0B3ZZ | BEAM RADIATION OF TIBIA/FIBULA USING ELECTRONS |
| ICD10_proc | DP0C3ZZ | BEAM RADIATION OF OTHER BONE USING ELECTRONS |
| ICD10_proc | DP003ZZ | BEAM RADIATION OF SKULL USING ELECTRONS |
| ICD10_proc | DP023ZZ | BEAM RADIATION OF MAXILLA USING ELECTRONS |
| ICD10_proc | DP033ZZ | BEAM RADIATION OF MANDIBLE USING ELECTRONS |
| ICD10_proc | DP043ZZ | BEAM RADIATION OF STERNUM USING ELECTRONS |
| ICD10_proc | DP053ZZ | BEAM RADIATION OF RIB(S) USING ELECTRONS |
| ICD10_proc | DP063ZZ | BEAM RADIATION OF HUMERUS USING ELECTRONS |
| ICD10_proc | DP073ZZ | BEAM RADIATION OF RADIUS/ULNA USING ELECTRONS |
| ICD10_proc | DP083ZZ | BEAM RADIATION OF PELVIC BONES USING ELECTRONS |
| ICD10_proc | DP093ZZ | BEAM RADIATION OF FEMUR USING ELECTRONS |
| ICD10_proc | DT003ZZ | BEAM RADIATION OF KIDNEY USING ELECTRONS |
| ICD10_proc | DT013ZZ | BEAM RADIATION OF URETER USING ELECTRONS |
| ICD10_proc | DT023ZZ | BEAM RADIATION OF BLADDER USING ELECTRONS |
| ICD10_proc | DT033ZZ | BEAM RADIATION OF URETHRA USING ELECTRONS |
| ICD10_proc | DU003ZZ | BEAM RADIATION OF OVARY USING ELECTRONS |
| ICD10_proc | DU013ZZ | BEAM RADIATION OF CERVIX USING ELECTRONS |
| ICD10_proc | DU023ZZ | BEAM RADIATION OF UTERUS USING ELECTRONS |
| ICD10_proc | DV003ZZ | BEAM RADIATION OF PROSTATE USING ELECTRONS |
| ICD10_proc | DV013ZZ | BEAM RADIATION OF TESTIS USING ELECTRONS |
| ICD10_proc | DW013ZZ | BEAM RADIATION OF HEAD AND NECK USING ELECTRONS |
| ICD10_proc | DW023ZZ | BEAM RADIATION OF CHEST USING ELECTRONS |
| ICD10_proc | DW033ZZ | BEAM RADIATION OF ABDOMEN USING ELECTRONS |
| ICD10_proc | DW043ZZ | BEAM RADIATION OF HEMIBODY USING ELECTRONS |
| ICD10_proc | DW053ZZ | BEAM RADIATION OF WHOLE BODY USING ELECTRONS |
| ICD10_proc | DW063ZZ | BEAM RADIATION OF PELVIC REGION USING ELECTRONS |
| ICD10_proc | D0003ZZ | BEAM RADIATION OF BRAIN USING ELECTRONS |
| ICD10_proc | D0013ZZ | BEAM RADIATION OF BRAIN STEM USING ELECTRONS |
| ICD10_proc | D0063ZZ | BEAM RADIATION OF SPINAL CORD USING ELECTRONS |
| ICD10_proc | D0073ZZ | BEAM RADIATION OF PERIPHERAL NERVE USING ELECTRONS |
| ICD10_proc | D7003ZZ | BEAM RADIATION OF BONE MARROW USING ELECTRONS |
| ICD10_proc | D7013ZZ | BEAM RADIATION OF THYMUS USING ELECTRONS |
| ICD10_proc | D7023ZZ | BEAM RADIATION OF SPLEEN USING ELECTRONS |
| ICD10_proc | D7033ZZ | BEAM RADIATION OF NECK LYMPHATICS USING ELECTRONS |
| ICD10_proc | D7043ZZ | BEAM RADIATION OF AXILLARY LYMPHATICS USING ELECTRONS |
| ICD10_proc | D7053ZZ | BEAM RADIATION OF THORAX LYMPHATICS USING ELECTRONS |
| ICD10_proc | D7063ZZ | BEAM RADIATION OF ABDOMEN LYMPHATICS USING ELECTRONS |
| ICD10_proc | D7073ZZ | BEAM RADIATION OF PELVIS LYMPHATICS USING ELECTRONS |
| ICD10_proc | D7083ZZ | BEAM RADIATION OF INGUINAL LYMPHATICS USING ELECTRONS |
| ICD10_proc | D8003ZZ | BEAM RADIATION OF EYE USING ELECTRONS |
| ICD10_proc | D90B3ZZ | BEAM RADIATION OF LARYNX USING ELECTRONS |
| ICD10_proc | D90D3ZZ | BEAM RADIATION OF NASOPHARYNX USING ELECTRONS |
| ICD10_proc | D90F3ZZ | BEAM RADIATION OF OROPHARYNX USING ELECTRONS |
| ICD10_proc | D9003ZZ | BEAM RADIATION OF EAR USING ELECTRONS |
| ICD10_proc | D9013ZZ | BEAM RADIATION OF NOSE USING ELECTRONS |
| ICD10_proc | D9033ZZ | BEAM RADIATION OF HYPOPHARYNX USING ELECTRONS |
| ICD10_proc | D9043ZZ | BEAM RADIATION OF MOUTH USING ELECTRONS |
| ICD10_proc | D9053ZZ | BEAM RADIATION OF TONGUE USING ELECTRONS |
| ICD10_proc | D9063ZZ | BEAM RADIATION OF SALIVARY GLANDS USING ELECTRONS |
| ICD10_proc | D9073ZZ | BEAM RADIATION OF SINUSES USING ELECTRONS |
| ICD10_proc | D9083ZZ | BEAM RADIATION OF HARD PALATE USING ELECTRONS |
| ICD10_proc | D9093ZZ | BEAM RADIATION OF SOFT PALATE USING ELECTRONS |
| ICD10_proc | DB004ZZ | BEAM RADIATION OF TRACHEA USING HEAVY PARTICLES |
| ICD10_proc | DB005ZZ | BEAM RADIATION OF TRACHEA USING NEUTRONS |
| ICD10_proc | DB014ZZ | BEAM RADIATION OF BRONCHUS USING HEAVY PARTICLES |
| ICD10_proc | DB015ZZ | BEAM RADIATION OF BRONCHUS USING NEUTRONS |
| ICD10_proc | DB024ZZ | BEAM RADIATION OF LUNG USING HEAVY PARTICLES (PROTONS,IONS) |
| ICD10_proc | DB025ZZ | BEAM RADIATION OF LUNG USING NEUTRONS |
| ICD10_proc | DB054ZZ | BEAM RADIATION OF PLEURA USING HEAVY PARTICLES |
| ICD10_proc | DB055ZZ | BEAM RADIATION OF PLEURA USING NEUTRONS |
| ICD10_proc | DB064ZZ | BEAM RADIATION OF MEDIASTINUM USING HEAVY PARTICLES |
| ICD10_proc | DB065ZZ | BEAM RADIATION OF MEDIASTINUM USING NEUTRONS |
| ICD10_proc | DB074ZZ | BEAM RADIATION OF CHEST WALL USING HEAVY PARTICLES |
| ICD10_proc | DB075ZZ | BEAM RADIATION OF CHEST WALL USING NEUTRONS |
| ICD10_proc | DB084ZZ | BEAM RADIATION OF DIAPHRAGM USING HEAVY PARTICLES |
| ICD10_proc | DB085ZZ | BEAM RADIATION OF DIAPHRAGM USING NEUTRONS |
| ICD10_proc | DD004ZZ | BEAM RADIATION OF ESOPHAGUS USING HEAVY PARTICLES |
| ICD10_proc | DD005ZZ | BEAM RADIATION OF ESOPHAGUS USING NEUTRONS |
| ICD10_proc | DD014ZZ | BEAM RADIATION OF STOMACH USING HEAVY PARTICLES |
| ICD10_proc | DD015ZZ | BEAM RADIATION OF STOMACH USING NEUTRONS |
| ICD10_proc | DD024ZZ | BEAM RADIATION OF DUODENUM USING HEAVY PARTICLES |
| ICD10_proc | DD025ZZ | BEAM RADIATION OF DUODENUM USING NEUTRONS |
| ICD10_proc | DD034ZZ | BEAM RADIATION OF JEJUNUM USING HEAVY PARTICLES |
| ICD10_proc | DD035ZZ | BEAM RADIATION OF JEJUNUM USING NEUTRONS |
| ICD10_proc | DD044ZZ | BEAM RADIATION OF ILEUM USING HEAVY PARTICLES (PROTONS,IONS) |
| ICD10_proc | DD045ZZ | BEAM RADIATION OF ILEUM USING NEUTRONS |
| ICD10_proc | DD054ZZ | BEAM RADIATION OF COLON USING HEAVY PARTICLES (PROTONS,IONS) |
| ICD10_proc | DD055ZZ | BEAM RADIATION OF COLON USING NEUTRONS |
| ICD10_proc | DD074ZZ | BEAM RADIATION OF RECTUM USING HEAVY PARTICLES |
| ICD10_proc | DD075ZZ | BEAM RADIATION OF RECTUM USING NEUTRONS |
| ICD10_proc | DF004ZZ | BEAM RADIATION OF LIVER USING HEAVY PARTICLES (PROTONS,IONS) |
| ICD10_proc | DF005ZZ | BEAM RADIATION OF LIVER USING NEUTRONS |
| ICD10_proc | DF014ZZ | BEAM RADIATION OF GALLBLADDER USING HEAVY PARTICLES |
| ICD10_proc | DF015ZZ | BEAM RADIATION OF GALLBLADDER USING NEUTRONS |
| ICD10_proc | DF024ZZ | BEAM RADIATION OF BILE DUCTS USING HEAVY PARTICLES |
| ICD10_proc | DF025ZZ | BEAM RADIATION OF BILE DUCTS USING NEUTRONS |
| ICD10_proc | DF034ZZ | BEAM RADIATION OF PANCREAS USING HEAVY PARTICLES |
| ICD10_proc | DF035ZZ | BEAM RADIATION OF PANCREAS USING NEUTRONS |
| ICD10_proc | DG005ZZ | BEAM RADIATION OF PITUITARY GLAND USING NEUTRONS |
| ICD10_proc | DG015ZZ | BEAM RADIATION OF PINEAL BODY USING NEUTRONS |
| ICD10_proc | DG025ZZ | BEAM RADIATION OF ADRENAL GLANDS USING NEUTRONS |
| ICD10_proc | DG045ZZ | BEAM RADIATION OF PARATHYROID GLANDS USING NEUTRONS |
| ICD10_proc | DG055ZZ | BEAM RADIATION OF THYROID USING NEUTRONS |
| ICD10_proc | DH0B4ZZ | BEAM RADIATION OF LEG SKIN USING HEAVY PARTICLES |
| ICD10_proc | DH0B5ZZ | BEAM RADIATION OF LEG SKIN USING NEUTRONS |
| ICD10_proc | DH024ZZ | BEAM RADIATION OF FACE SKIN USING HEAVY PARTICLES |
| ICD10_proc | DH025ZZ | BEAM RADIATION OF FACE SKIN USING NEUTRONS |
| ICD10_proc | DH034ZZ | BEAM RADIATION OF NECK SKIN USING HEAVY PARTICLES |
| ICD10_proc | DH035ZZ | BEAM RADIATION OF NECK SKIN USING NEUTRONS |
| ICD10_proc | DH044ZZ | BEAM RADIATION OF ARM SKIN USING HEAVY PARTICLES |
| ICD10_proc | DH045ZZ | BEAM RADIATION OF ARM SKIN USING NEUTRONS |
| ICD10_proc | DH064ZZ | BEAM RADIATION OF CHEST SKIN USING HEAVY PARTICLES |
| ICD10_proc | DH065ZZ | BEAM RADIATION OF CHEST SKIN USING NEUTRONS |
| ICD10_proc | DH074ZZ | BEAM RADIATION OF BACK SKIN USING HEAVY PARTICLES |
| ICD10_proc | DH075ZZ | BEAM RADIATION OF BACK SKIN USING NEUTRONS |
| ICD10_proc | DH084ZZ | BEAM RADIATION OF ABDOMEN SKIN USING HEAVY PARTICLES |
| ICD10_proc | DH085ZZ | BEAM RADIATION OF ABDOMEN SKIN USING NEUTRONS |
| ICD10_proc | DH094ZZ | BEAM RADIATION OF BUTTOCK SKIN USING HEAVY PARTICLES |
| ICD10_proc | DH095ZZ | BEAM RADIATION OF BUTTOCK SKIN USING NEUTRONS |
| ICD10_proc | DM004ZZ | BEAM RADIATION OF LEFT BREAST USING HEAVY PARTICLES |
| ICD10_proc | DM005ZZ | BEAM RADIATION OF LEFT BREAST USING NEUTRONS |
| ICD10_proc | DM014ZZ | BEAM RADIATION OF RIGHT BREAST USING HEAVY PARTICLES |
| ICD10_proc | DM015ZZ | BEAM RADIATION OF RIGHT BREAST USING NEUTRONS |
| ICD10_proc | DP0B4ZZ | BEAM RADIATION OF TIBIA/FIBULA USING HEAVY PARTICLES |
| ICD10_proc | DP0B5ZZ | BEAM RADIATION OF TIBIA/FIBULA USING NEUTRONS |
| ICD10_proc | DP0C4ZZ | BEAM RADIATION OF OTHER BONE USING HEAVY PARTICLES |
| ICD10_proc | DP0C5ZZ | BEAM RADIATION OF OTHER BONE USING NEUTRONS |
| ICD10_proc | DP004ZZ | BEAM RADIATION OF SKULL USING HEAVY PARTICLES (PROTONS,IONS) |
| ICD10_proc | DP005ZZ | BEAM RADIATION OF SKULL USING NEUTRONS |
| ICD10_proc | DP024ZZ | BEAM RADIATION OF MAXILLA USING HEAVY PARTICLES |
| ICD10_proc | DP025ZZ | BEAM RADIATION OF MAXILLA USING NEUTRONS |
| ICD10_proc | DP034ZZ | BEAM RADIATION OF MANDIBLE USING HEAVY PARTICLES |
| ICD10_proc | DP035ZZ | BEAM RADIATION OF MANDIBLE USING NEUTRONS |
| ICD10_proc | DP044ZZ | BEAM RADIATION OF STERNUM USING HEAVY PARTICLES |
| ICD10_proc | DP045ZZ | BEAM RADIATION OF STERNUM USING NEUTRONS |
| ICD10_proc | DP054ZZ | BEAM RADIATION OF RIB(S) USING HEAVY PARTICLES |
| ICD10_proc | DP055ZZ | BEAM RADIATION OF RIB(S) USING NEUTRONS |
| ICD10_proc | DP064ZZ | BEAM RADIATION OF HUMERUS USING HEAVY PARTICLES |
| ICD10_proc | DP065ZZ | BEAM RADIATION OF HUMERUS USING NEUTRONS |
| ICD10_proc | DP074ZZ | BEAM RADIATION OF RADIUS/ULNA USING HEAVY PARTICLES |
| ICD10_proc | DP075ZZ | BEAM RADIATION OF RADIUS/ULNA USING NEUTRONS |
| ICD10_proc | DP084ZZ | BEAM RADIATION OF PELVIC BONES USING HEAVY PARTICLES |
| ICD10_proc | DP085ZZ | BEAM RADIATION OF PELVIC BONES USING NEUTRONS |
| ICD10_proc | DP094ZZ | BEAM RADIATION OF FEMUR USING HEAVY PARTICLES (PROTONS,IONS) |
| ICD10_proc | DP095ZZ | BEAM RADIATION OF FEMUR USING NEUTRONS |
| ICD10_proc | DT004ZZ | BEAM RADIATION OF KIDNEY USING HEAVY PARTICLES |
| ICD10_proc | DT005ZZ | BEAM RADIATION OF KIDNEY USING NEUTRONS |
| ICD10_proc | DT014ZZ | BEAM RADIATION OF URETER USING HEAVY PARTICLES |
| ICD10_proc | DT015ZZ | BEAM RADIATION OF URETER USING NEUTRONS |
| ICD10_proc | DT024ZZ | BEAM RADIATION OF BLADDER USING HEAVY PARTICLES |
| ICD10_proc | DT025ZZ | BEAM RADIATION OF BLADDER USING NEUTRONS |
| ICD10_proc | DT034ZZ | BEAM RADIATION OF URETHRA USING HEAVY PARTICLES |
| ICD10_proc | DT035ZZ | BEAM RADIATION OF URETHRA USING NEUTRONS |
| ICD10_proc | DU004ZZ | BEAM RADIATION OF OVARY USING HEAVY PARTICLES (PROTONS,IONS) |
| ICD10_proc | DU005ZZ | BEAM RADIATION OF OVARY USING NEUTRONS |
| ICD10_proc | DU014ZZ | BEAM RADIATION OF CERVIX USING HEAVY PARTICLES |
| ICD10_proc | DU015ZZ | BEAM RADIATION OF CERVIX USING NEUTRONS |
| ICD10_proc | DU024ZZ | BEAM RADIATION OF UTERUS USING HEAVY PARTICLES |
| ICD10_proc | DU025ZZ | BEAM RADIATION OF UTERUS USING NEUTRONS |
| ICD10_proc | DV004ZZ | BEAM RADIATION OF PROSTATE USING HEAVY PARTICLES |
| ICD10_proc | DV005ZZ | BEAM RADIATION OF PROSTATE USING NEUTRONS |
| ICD10_proc | DV014ZZ | BEAM RADIATION OF TESTIS USING HEAVY PARTICLES |
| ICD10_proc | DV015ZZ | BEAM RADIATION OF TESTIS USING NEUTRONS |
| ICD10_proc | DW014ZZ | BEAM RADIATION OF HEAD AND NECK USING HEAVY PARTICLES |
| ICD10_proc | DW015ZZ | BEAM RADIATION OF HEAD AND NECK USING NEUTRONS |
| ICD10_proc | DW024ZZ | BEAM RADIATION OF CHEST USING HEAVY PARTICLES (PROTONS,IONS) |
| ICD10_proc | DW025ZZ | BEAM RADIATION OF CHEST USING NEUTRONS |
| ICD10_proc | DW034ZZ | BEAM RADIATION OF ABDOMEN USING HEAVY PARTICLES |
| ICD10_proc | DW035ZZ | BEAM RADIATION OF ABDOMEN USING NEUTRONS |
| ICD10_proc | DW044ZZ | BEAM RADIATION OF HEMIBODY USING HEAVY PARTICLES |
| ICD10_proc | DW045ZZ | BEAM RADIATION OF HEMIBODY USING NEUTRONS |
| ICD10_proc | DW054ZZ | BEAM RADIATION OF WHOLE BODY USING HEAVY PARTICLES |
| ICD10_proc | DW055ZZ | BEAM RADIATION OF WHOLE BODY USING NEUTRONS |
| ICD10_proc | DW064ZZ | BEAM RADIATION OF PELVIC REGION USING HEAVY PARTICLES |
| ICD10_proc | DW065ZZ | BEAM RADIATION OF PELVIC REGION USING NEUTRONS |
| ICD10_proc | D0004ZZ | BEAM RADIATION OF BRAIN USING HEAVY PARTICLES (PROTONS,IONS) |
| ICD10_proc | D0005ZZ | BEAM RADIATION OF BRAIN USING NEUTRONS |
| ICD10_proc | D0014ZZ | BEAM RADIATION OF BRAIN STEM USING HEAVY PARTICLES |
| ICD10_proc | D0015ZZ | BEAM RADIATION OF BRAIN STEM USING NEUTRONS |
| ICD10_proc | D0064ZZ | BEAM RADIATION OF SPINAL CORD USING HEAVY PARTICLES |
| ICD10_proc | D0065ZZ | BEAM RADIATION OF SPINAL CORD USING NEUTRONS |
| ICD10_proc | D0074ZZ | BEAM RADIATION OF PERIPHERAL NERVE USING HEAVY PARTICLES |
| ICD10_proc | D0075ZZ | BEAM RADIATION OF PERIPHERAL NERVE USING NEUTRONS |
| ICD10_proc | D7004ZZ | BEAM RADIATION OF BONE MARROW USING HEAVY PARTICLES |
| ICD10_proc | D7005ZZ | BEAM RADIATION OF BONE MARROW USING NEUTRONS |
| ICD10_proc | D7014ZZ | BEAM RADIATION OF THYMUS USING HEAVY PARTICLES |
| ICD10_proc | D7015ZZ | BEAM RADIATION OF THYMUS USING NEUTRONS |
| ICD10_proc | D7024ZZ | BEAM RADIATION OF SPLEEN USING HEAVY PARTICLES |
| ICD10_proc | D7025ZZ | BEAM RADIATION OF SPLEEN USING NEUTRONS |
| ICD10_proc | D7034ZZ | BEAM RADIATION OF NECK LYMPHATICS USING HEAVY PARTICLES |
| ICD10_proc | D7035ZZ | BEAM RADIATION OF NECK LYMPHATICS USING NEUTRONS |
| ICD10_proc | D7044ZZ | BEAM RADIATION OF AXILLARY LYMPHATICS USING HEAVY PARTICLES |
| ICD10_proc | D7045ZZ | BEAM RADIATION OF AXILLARY LYMPHATICS USING NEUTRONS |
| ICD10_proc | D7054ZZ | BEAM RADIATION OF THORAX LYMPHATICS USING HEAVY PARTICLES |
| ICD10_proc | D7055ZZ | BEAM RADIATION OF THORAX LYMPHATICS USING NEUTRONS |
| ICD10_proc | D7064ZZ | BEAM RADIATION OF ABDOMEN LYMPHATICS USING HEAVY PARTICLES |
| ICD10_proc | D7065ZZ | BEAM RADIATION OF ABDOMEN LYMPHATICS USING NEUTRONS |
| ICD10_proc | D7074ZZ | BEAM RADIATION OF PELVIS LYMPHATICS USING HEAVY PARTICLES |
| ICD10_proc | D7075ZZ | BEAM RADIATION OF PELVIS LYMPHATICS USING NEUTRONS |
| ICD10_proc | D7084ZZ | BEAM RADIATION OF INGUINAL LYMPHATICS USING HEAVY PARTICLES |
| ICD10_proc | D7085ZZ | BEAM RADIATION OF INGUINAL LYMPHATICS USING NEUTRONS |
| ICD10_proc | D8004ZZ | BEAM RADIATION OF EYE USING HEAVY PARTICLES (PROTONS,IONS) |
| ICD10_proc | D8005ZZ | BEAM RADIATION OF EYE USING NEUTRONS |
| ICD10_proc | D90B4ZZ | BEAM RADIATION OF LARYNX USING HEAVY PARTICLES |
| ICD10_proc | D90B5ZZ | BEAM RADIATION OF LARYNX USING NEUTRONS |
| ICD10_proc | D90D4ZZ | BEAM RADIATION OF NASOPHARYNX USING HEAVY PARTICLES |
| ICD10_proc | D90D5ZZ | BEAM RADIATION OF NASOPHARYNX USING NEUTRONS |
| ICD10_proc | D90F4ZZ | BEAM RADIATION OF OROPHARYNX USING HEAVY PARTICLES |
| ICD10_proc | D90F5ZZ | BEAM RADIATION OF OROPHARYNX USING NEUTRONS |
| ICD10_proc | D9004ZZ | BEAM RADIATION OF EAR USING HEAVY PARTICLES (PROTONS,IONS) |
| ICD10_proc | D9005ZZ | BEAM RADIATION OF EAR USING NEUTRONS |
| ICD10_proc | D9014ZZ | BEAM RADIATION OF NOSE USING HEAVY PARTICLES (PROTONS,IONS) |
| ICD10_proc | D9015ZZ | BEAM RADIATION OF NOSE USING NEUTRONS |
| ICD10_proc | D9034ZZ | BEAM RADIATION OF HYPOPHARYNX USING HEAVY PARTICLES |
| ICD10_proc | D9035ZZ | BEAM RADIATION OF HYPOPHARYNX USING NEUTRONS |
| ICD10_proc | D9044ZZ | BEAM RADIATION OF MOUTH USING HEAVY PARTICLES (PROTONS,IONS) |
| ICD10_proc | D9045ZZ | BEAM RADIATION OF MOUTH USING NEUTRONS |
| ICD10_proc | D9054ZZ | BEAM RADIATION OF TONGUE USING HEAVY PARTICLES |
| ICD10_proc | D9055ZZ | BEAM RADIATION OF TONGUE USING NEUTRONS |
| ICD10_proc | D9064ZZ | BEAM RADIATION OF SALIVARY GLANDS USING HEAVY PARTICLES |
| ICD10_proc | D9065ZZ | BEAM RADIATION OF SALIVARY GLANDS USING NEUTRONS |
| ICD10_proc | D9074ZZ | BEAM RADIATION OF SINUSES USING HEAVY PARTICLES |
| ICD10_proc | D9075ZZ | BEAM RADIATION OF SINUSES USING NEUTRONS |
| ICD10_proc | D9084ZZ | BEAM RADIATION OF HARD PALATE USING HEAVY PARTICLES |
| ICD10_proc | D9085ZZ | BEAM RADIATION OF HARD PALATE USING NEUTRONS |
| ICD10_proc | D9094ZZ | BEAM RADIATION OF SOFT PALATE USING HEAVY PARTICLES |
| ICD10_proc | D9095ZZ | BEAM RADIATION OF SOFT PALATE USING NEUTRONS |
| ICD10_proc | 0BHK01Z | INSERTION OF RADIOACTIVE ELEMENT INTO R LUNG, OPEN APPROACH |
| ICD10_proc | 0UHC01Z | INSERTION OF RADIOACTIVE ELEMENT INTO CERVIX, OPEN APPROACH |
| ICD10_proc | 0UHC31Z | INSERTION OF RADIOACTIVE ELEMENT INTO CERVIX, PERC APPROACH |
| ICD10_proc | 0UHC41Z | INSERTION OF RADIOACT ELEM INTO CERVIX, PERC ENDO APPROACH |
| ICD10_proc | 0UHC71Z | INSERTION OF RADIOACTIVE ELEMENT INTO CERVIX, VIA OPENING |
| ICD10_proc | 0UHC81Z | INSERTION OF RADIOACTIVE ELEMENT INTO CERVIX, ENDO |
| ICD10_proc | 0UHGX1Z | INSERTION OF RADIOACT ELEM INTO VAGINA, EXTERN APPROACH |
| ICD10_proc | 0UHG01Z | INSERTION OF RADIOACTIVE ELEMENT INTO VAGINA, OPEN APPROACH |
| ICD10_proc | 0UHG31Z | INSERTION OF RADIOACTIVE ELEMENT INTO VAGINA, PERC APPROACH |
| ICD10_proc | 0UHG41Z | INSERTION OF RADIOACT ELEM INTO VAGINA, PERC ENDO APPROACH |
| ICD10_proc | 0UHG71Z | INSERTION OF RADIOACTIVE ELEMENT INTO VAGINA, VIA OPENING |
| ICD10_proc | 0UHG81Z | INSERTION OF RADIOACTIVE ELEMENT INTO VAGINA, ENDO |
| ICD10_proc | 0VH001Z | INSERTION OF RADIOACT ELEM INTO PROSTATE, OPEN APPROACH |
| ICD10_proc | 0VH031Z | INSERTION OF RADIOACT ELEM INTO PROSTATE, PERC APPROACH |
| ICD10_proc | 0VH041Z | INSERTION OF RADIOACT ELEM INTO PROSTATE, PERC ENDO APPROACH |
| ICD10_proc | 0VH071Z | INSERTION OF RADIOACTIVE ELEMENT INTO PROSTATE, VIA OPENING |
| ICD10_proc | 0VH081Z | INSERTION OF RADIOACTIVE ELEMENT INTO PROSTATE, ENDO |
| ICD10_proc | 0WHB01Z | INSERTION OF RADIOACT ELEM INTO L PLEURAL CAV, OPEN APPROACH |
| ICD10_proc | 0WHB31Z | INSERTION OF RADIOACT ELEM INTO L PLEURAL CAV, PERC APPROACH |
| ICD10_proc | 0WHB41Z | INSERT RADIOACT ELEM IN L PLEURAL CAV, PERC ENDO |
| ICD10_proc | 0WHC01Z | INSERTION OF RADIOACT ELEM INTO MEDIASTINUM, OPEN APPROACH |
| ICD10_proc | 0WHC31Z | INSERTION OF RADIOACT ELEM INTO MEDIASTINUM, PERC APPROACH |
| ICD10_proc | 0WHC41Z | INSERT OF RADIOACT ELEM INTO MEDIASTINUM, PERC ENDO APPROACH |
| ICD10_proc | 0WHD01Z | INSERTION OF RADIOACT ELEM INTO PERICARD CAV, OPEN APPROACH |
| ICD10_proc | 0WHD31Z | INSERTION OF RADIOACT ELEM INTO PERICARD CAV, PERC APPROACH |
| ICD10_proc | 0WHD41Z | INSERT RADIOACT ELEM IN PERICARD CAV, PERC ENDO |
| ICD10_proc | 0WHF01Z | INSERTION OF RADIOACT ELEM INTO ABD WALL, OPEN APPROACH |
| ICD10_proc | 0WHF31Z | INSERTION OF RADIOACT ELEM INTO ABD WALL, PERC APPROACH |
| ICD10_proc | 0WHF41Z | INSERTION OF RADIOACT ELEM INTO ABD WALL, PERC ENDO APPROACH |
| ICD10_proc | 0WHG01Z | INSERTION OF RADIOACT ELEM INTO PERITON CAV, OPEN APPROACH |
| ICD10_proc | 0WHG31Z | INSERTION OF RADIOACT ELEM INTO PERITON CAV, PERC APPROACH |
| ICD10_proc | 0WHG41Z | INSERT OF RADIOACT ELEM INTO PERITON CAV, PERC ENDO APPROACH |
| ICD10_proc | 0WHH01Z | INSERT OF RADIOACT ELEM INTO RETROPERITONEUM, OPEN APPROACH |
| ICD10_proc | 0WHH31Z | INSERT OF RADIOACT ELEM INTO RETROPERITONEUM, PERC APPROACH |
| ICD10_proc | 0WHH41Z | INSERT RADIOACT ELEM IN RETROPERITONEUM, PERC ENDO |
| ICD10_proc | 0WHJ01Z | INSERTION OF RADIOACT ELEM INTO PELVIC CAV, OPEN APPROACH |
| ICD10_proc | 0WHJ31Z | INSERTION OF RADIOACT ELEM INTO PELVIC CAV, PERC APPROACH |
| ICD10_proc | 0WHJ41Z | INSERT OF RADIOACT ELEM INTO PELVIC CAV, PERC ENDO APPROACH |
| ICD10_proc | 0WHK01Z | INSERTION OF RADIOACTIVE ELEMENT INTO UP BACK, OPEN APPROACH |
| ICD10_proc | 0WHK31Z | INSERTION OF RADIOACTIVE ELEMENT INTO UP BACK, PERC APPROACH |
| ICD10_proc | 0WHK41Z | INSERTION OF RADIOACT ELEM INTO UP BACK, PERC ENDO APPROACH |
| ICD10_proc | 0WHL01Z | INSERTION OF RADIOACT ELEM INTO LOW BACK, OPEN APPROACH |
| ICD10_proc | 0WHL31Z | INSERTION OF RADIOACT ELEM INTO LOW BACK, PERC APPROACH |
| ICD10_proc | 0WHL41Z | INSERTION OF RADIOACT ELEM INTO LOW BACK, PERC ENDO APPROACH |
| ICD10_proc | 0WHM01Z | INSERTION OF RADIOACT ELEM INTO MALE PERINEUM, OPEN APPROACH |
| ICD10_proc | 0WHM31Z | INSERTION OF RADIOACT ELEM INTO MALE PERINEUM, PERC APPROACH |
| ICD10_proc | 0WHM41Z | INSERT RADIOACT ELEM IN MALE PERINEUM, PERC ENDO |
| ICD10_proc | 0WHN01Z | INSERTION OF RADIOACT ELEM INTO FEM PERINEUM, OPEN APPROACH |
| ICD10_proc | 0WHN31Z | INSERTION OF RADIOACT ELEM INTO FEM PERINEUM, PERC APPROACH |
| ICD10_proc | 0WHN41Z | INSERT RADIOACT ELEM IN FEM PERINEUM, PERC ENDO |
| ICD10_proc | 0WHP01Z | INSERTION OF RADIOACT ELEM INTO GI TRACT, OPEN APPROACH |
| ICD10_proc | 0WHP31Z | INSERTION OF RADIOACT ELEM INTO GI TRACT, PERC APPROACH |
| ICD10_proc | 0WHP41Z | INSERTION OF RADIOACT ELEM INTO GI TRACT, PERC ENDO APPROACH |
| ICD10_proc | 0WHP71Z | INSERTION OF RADIOACTIVE ELEMENT INTO GI TRACT, VIA OPENING |
| ICD10_proc | 0WHP81Z | INSERTION OF RADIOACTIVE ELEMENT INTO GI TRACT, ENDO |
| ICD10_proc | 0WHQ01Z | INSERTION OF RADIOACT ELEM INTO RESP TRACT, OPEN APPROACH |
| ICD10_proc | 0WHQ31Z | INSERTION OF RADIOACT ELEM INTO RESP TRACT, PERC APPROACH |
| ICD10_proc | 0WHQ41Z | INSERT OF RADIOACT ELEM INTO RESP TRACT, PERC ENDO APPROACH |
| ICD10_proc | 0WHQ71Z | INSERTION OF RADIOACT ELEM INTO RESP TRACT, VIA OPENING |
| ICD10_proc | 0WHQ81Z | INSERTION OF RADIOACTIVE ELEMENT INTO RESP TRACT, ENDO |
| ICD10_proc | 0WHR01Z | INSERTION OF RADIOACT ELEM INTO GU TRACT, OPEN APPROACH |
| ICD10_proc | 0WHR31Z | INSERTION OF RADIOACT ELEM INTO GU TRACT, PERC APPROACH |
| ICD10_proc | 0WHR41Z | INSERTION OF RADIOACT ELEM INTO GU TRACT, PERC ENDO APPROACH |
| ICD10_proc | 0WHR71Z | INSERTION OF RADIOACTIVE ELEMENT INTO GU TRACT, VIA OPENING |
| ICD10_proc | 0WHR81Z | INSERTION OF RADIOACTIVE ELEMENT INTO GU TRACT, ENDO |
| ICD10_proc | 0WH001Z | INSERTION OF RADIOACTIVE ELEMENT INTO HEAD, OPEN APPROACH |
| ICD10_proc | 0WH031Z | INSERTION OF RADIOACTIVE ELEMENT INTO HEAD, PERC APPROACH |
| ICD10_proc | 0WH041Z | INSERTION OF RADIOACT ELEM INTO HEAD, PERC ENDO APPROACH |
| ICD10_proc | 0WH101Z | INSERTION OF RADIOACT ELEM INTO CRANIAL CAV, OPEN APPROACH |
| ICD10_proc | 0WH131Z | INSERTION OF RADIOACT ELEM INTO CRANIAL CAV, PERC APPROACH |
| ICD10_proc | 0WH141Z | INSERT OF RADIOACT ELEM INTO CRANIAL CAV, PERC ENDO APPROACH |
| ICD10_proc | 0WH201Z | INSERTION OF RADIOACTIVE ELEMENT INTO FACE, OPEN APPROACH |
| ICD10_proc | 0WH231Z | INSERTION OF RADIOACTIVE ELEMENT INTO FACE, PERC APPROACH |
| ICD10_proc | 0WH241Z | INSERTION OF RADIOACT ELEM INTO FACE, PERC ENDO APPROACH |
| ICD10_proc | 0WH301Z | INSERT RADIOACT ELEM IN ORAL CAV & THROAT, OPEN |
| ICD10_proc | 0WH331Z | INSERT RADIOACT ELEM IN ORAL CAV & THROAT, PERC |
| ICD10_proc | 0WH341Z | INSERT RADIOACT ELEM IN ORAL CAV & THROAT, PERC ENDO |
| ICD10_proc | 0WH401Z | INSERTION OF RADIOACTIVE ELEMENT INTO UP JAW, OPEN APPROACH |
| ICD10_proc | 0WH431Z | INSERTION OF RADIOACTIVE ELEMENT INTO UP JAW, PERC APPROACH |
| ICD10_proc | 0WH441Z | INSERTION OF RADIOACT ELEM INTO UP JAW, PERC ENDO APPROACH |
| ICD10_proc | 0WH501Z | INSERTION OF RADIOACTIVE ELEMENT INTO LOW JAW, OPEN APPROACH |
| ICD10_proc | 0WH531Z | INSERTION OF RADIOACTIVE ELEMENT INTO LOW JAW, PERC APPROACH |
| ICD10_proc | 0WH541Z | INSERTION OF RADIOACT ELEM INTO LOW JAW, PERC ENDO APPROACH |
| ICD10_proc | 0WH601Z | INSERTION OF RADIOACTIVE ELEMENT INTO NECK, OPEN APPROACH |
| ICD10_proc | 0WH631Z | INSERTION OF RADIOACTIVE ELEMENT INTO NECK, PERC APPROACH |
| ICD10_proc | 0WH641Z | INSERTION OF RADIOACT ELEM INTO NECK, PERC ENDO APPROACH |
| ICD10_proc | 0WH801Z | INSERTION OF RADIOACT ELEM INTO CHEST WALL, OPEN APPROACH |
| ICD10_proc | 0WH831Z | INSERTION OF RADIOACT ELEM INTO CHEST WALL, PERC APPROACH |
| ICD10_proc | 0WH841Z | INSERT OF RADIOACT ELEM INTO CHEST WALL, PERC ENDO APPROACH |
| ICD10_proc | 0WH901Z | INSERTION OF RADIOACT ELEM INTO R PLEURAL CAV, OPEN APPROACH |
| ICD10_proc | 0WH931Z | INSERTION OF RADIOACT ELEM INTO R PLEURAL CAV, PERC APPROACH |
| ICD10_proc | 0WH941Z | INSERT RADIOACT ELEM IN R PLEURAL CAV, PERC ENDO |
| ICD10_proc | 0XHB01Z | INSERTION OF RADIOACTIVE ELEMENT INTO R ELBOW, OPEN APPROACH |
| ICD10_proc | 0XHB31Z | INSERTION OF RADIOACTIVE ELEMENT INTO R ELBOW, PERC APPROACH |
| ICD10_proc | 0XHB41Z | INSERTION OF RADIOACT ELEM INTO R ELBOW, PERC ENDO APPROACH |
| ICD10_proc | 0XHC01Z | INSERTION OF RADIOACTIVE ELEMENT INTO L ELBOW, OPEN APPROACH |
| ICD10_proc | 0XHC31Z | INSERTION OF RADIOACTIVE ELEMENT INTO L ELBOW, PERC APPROACH |
| ICD10_proc | 0XHC41Z | INSERTION OF RADIOACT ELEM INTO L ELBOW, PERC ENDO APPROACH |
| ICD10_proc | 0XHD01Z | INSERTION OF RADIOACT ELEM INTO R LOW ARM, OPEN APPROACH |
| ICD10_proc | 0XHD31Z | INSERTION OF RADIOACT ELEM INTO R LOW ARM, PERC APPROACH |
| ICD10_proc | 0XHD41Z | INSERT OF RADIOACT ELEM INTO R LOW ARM, PERC ENDO APPROACH |
| ICD10_proc | 0XHF01Z | INSERTION OF RADIOACT ELEM INTO L LOW ARM, OPEN APPROACH |
| ICD10_proc | 0XHF31Z | INSERTION OF RADIOACT ELEM INTO L LOW ARM, PERC APPROACH |
| ICD10_proc | 0XHF41Z | INSERT OF RADIOACT ELEM INTO L LOW ARM, PERC ENDO APPROACH |
| ICD10_proc | 0XHG01Z | INSERTION OF RADIOACTIVE ELEMENT INTO R WRIST, OPEN APPROACH |
| ICD10_proc | 0XHG31Z | INSERTION OF RADIOACTIVE ELEMENT INTO R WRIST, PERC APPROACH |
| ICD10_proc | 0XHG41Z | INSERTION OF RADIOACT ELEM INTO R WRIST, PERC ENDO APPROACH |
| ICD10_proc | 0XHH01Z | INSERTION OF RADIOACTIVE ELEMENT INTO L WRIST, OPEN APPROACH |
| ICD10_proc | 0XHH31Z | INSERTION OF RADIOACTIVE ELEMENT INTO L WRIST, PERC APPROACH |
| ICD10_proc | 0XHH41Z | INSERTION OF RADIOACT ELEM INTO L WRIST, PERC ENDO APPROACH |
| ICD10_proc | 0XHJ01Z | INSERTION OF RADIOACTIVE ELEMENT INTO R HAND, OPEN APPROACH |
| ICD10_proc | 0XHJ31Z | INSERTION OF RADIOACTIVE ELEMENT INTO R HAND, PERC APPROACH |
| ICD10_proc | 0XHJ41Z | INSERTION OF RADIOACT ELEM INTO R HAND, PERC ENDO APPROACH |
| ICD10_proc | 0XHK01Z | INSERTION OF RADIOACTIVE ELEMENT INTO L HAND, OPEN APPROACH |
| ICD10_proc | 0XHK31Z | INSERTION OF RADIOACTIVE ELEMENT INTO L HAND, PERC APPROACH |
| ICD10_proc | 0XHK41Z | INSERTION OF RADIOACT ELEM INTO L HAND, PERC ENDO APPROACH |
| ICD10_proc | 0XH201Z | INSERTION OF RADIOACT ELEM INTO R SHOULDER, OPEN APPROACH |
| ICD10_proc | 0XH231Z | INSERTION OF RADIOACT ELEM INTO R SHOULDER, PERC APPROACH |
| ICD10_proc | 0XH241Z | INSERT OF RADIOACT ELEM INTO R SHOULDER, PERC ENDO APPROACH |
| ICD10_proc | 0XH301Z | INSERTION OF RADIOACT ELEM INTO L SHOULDER, OPEN APPROACH |
| ICD10_proc | 0XH331Z | INSERTION OF RADIOACT ELEM INTO L SHOULDER, PERC APPROACH |
| ICD10_proc | 0XH341Z | INSERT OF RADIOACT ELEM INTO L SHOULDER, PERC ENDO APPROACH |
| ICD10_proc | 0XH401Z | INSERTION OF RADIOACT ELEM INTO R AXILLA, OPEN APPROACH |
| ICD10_proc | 0XH431Z | INSERTION OF RADIOACT ELEM INTO R AXILLA, PERC APPROACH |
| ICD10_proc | 0XH441Z | INSERTION OF RADIOACT ELEM INTO R AXILLA, PERC ENDO APPROACH |
| ICD10_proc | 0XH501Z | INSERTION OF RADIOACT ELEM INTO L AXILLA, OPEN APPROACH |
| ICD10_proc | 0XH531Z | INSERTION OF RADIOACT ELEM INTO L AXILLA, PERC APPROACH |
| ICD10_proc | 0XH541Z | INSERTION OF RADIOACT ELEM INTO L AXILLA, PERC ENDO APPROACH |
| ICD10_proc | 0XH601Z | INSERTION OF RADIOACT ELEM INTO R UP EXTREM, OPEN APPROACH |
| ICD10_proc | 0XH631Z | INSERTION OF RADIOACT ELEM INTO R UP EXTREM, PERC APPROACH |
| ICD10_proc | 0XH641Z | INSERT OF RADIOACT ELEM INTO R UP EXTREM, PERC ENDO APPROACH |
| ICD10_proc | 0XH701Z | INSERTION OF RADIOACT ELEM INTO L UP EXTREM, OPEN APPROACH |
| ICD10_proc | 0XH731Z | INSERTION OF RADIOACT ELEM INTO L UP EXTREM, PERC APPROACH |
| ICD10_proc | 0XH741Z | INSERT OF RADIOACT ELEM INTO L UP EXTREM, PERC ENDO APPROACH |
| ICD10_proc | 0XH801Z | INSERTION OF RADIOACT ELEM INTO R UP ARM, OPEN APPROACH |
| ICD10_proc | 0XH831Z | INSERTION OF RADIOACT ELEM INTO R UP ARM, PERC APPROACH |
| ICD10_proc | 0XH841Z | INSERTION OF RADIOACT ELEM INTO R UP ARM, PERC ENDO APPROACH |
| ICD10_proc | 0XH901Z | INSERTION OF RADIOACT ELEM INTO L UP ARM, OPEN APPROACH |
| ICD10_proc | 0XH931Z | INSERTION OF RADIOACT ELEM INTO L UP ARM, PERC APPROACH |
| ICD10_proc | 0XH941Z | INSERTION OF RADIOACT ELEM INTO L UP ARM, PERC ENDO APPROACH |
| ICD10_proc | 0YHB01Z | INSERTION OF RADIOACT ELEM INTO L LOW EXTREM, OPEN APPROACH |
| ICD10_proc | 0YHB31Z | INSERTION OF RADIOACT ELEM INTO L LOW EXTREM, PERC APPROACH |
| ICD10_proc | 0YHB41Z | INSERT RADIOACT ELEM IN L LOW EXTREM, PERC ENDO |
| ICD10_proc | 0YHC01Z | INSERTION OF RADIOACT ELEM INTO R UP LEG, OPEN APPROACH |
| ICD10_proc | 0YHC31Z | INSERTION OF RADIOACT ELEM INTO R UP LEG, PERC APPROACH |
| ICD10_proc | 0YHC41Z | INSERTION OF RADIOACT ELEM INTO R UP LEG, PERC ENDO APPROACH |
| ICD10_proc | 0YHD01Z | INSERTION OF RADIOACT ELEM INTO L UP LEG, OPEN APPROACH |
| ICD10_proc | 0YHD31Z | INSERTION OF RADIOACT ELEM INTO L UP LEG, PERC APPROACH |
| ICD10_proc | 0YHD41Z | INSERTION OF RADIOACT ELEM INTO L UP LEG, PERC ENDO APPROACH |
| ICD10_proc | 0YHF01Z | INSERTION OF RADIOACTIVE ELEMENT INTO R KNEE, OPEN APPROACH |
| ICD10_proc | 0YHF31Z | INSERTION OF RADIOACTIVE ELEMENT INTO R KNEE, PERC APPROACH |
| ICD10_proc | 0YHF41Z | INSERTION OF RADIOACT ELEM INTO R KNEE, PERC ENDO APPROACH |
| ICD10_proc | 0YHG01Z | INSERTION OF RADIOACTIVE ELEMENT INTO L KNEE, OPEN APPROACH |
| ICD10_proc | 0YHG31Z | INSERTION OF RADIOACTIVE ELEMENT INTO L KNEE, PERC APPROACH |
| ICD10_proc | 0YHG41Z | INSERTION OF RADIOACT ELEM INTO L KNEE, PERC ENDO APPROACH |
| ICD10_proc | 0YHH01Z | INSERTION OF RADIOACT ELEM INTO R LOW LEG, OPEN APPROACH |
| ICD10_proc | 0YHH31Z | INSERTION OF RADIOACT ELEM INTO R LOW LEG, PERC APPROACH |
| ICD10_proc | 0YHH41Z | INSERT OF RADIOACT ELEM INTO R LOW LEG, PERC ENDO APPROACH |
| ICD10_proc | 0YHJ01Z | INSERTION OF RADIOACT ELEM INTO L LOW LEG, OPEN APPROACH |
| ICD10_proc | 0YHJ31Z | INSERTION OF RADIOACT ELEM INTO L LOW LEG, PERC APPROACH |
| ICD10_proc | 0YHJ41Z | INSERT OF RADIOACT ELEM INTO L LOW LEG, PERC ENDO APPROACH |
| ICD10_proc | 0YHK01Z | INSERTION OF RADIOACTIVE ELEMENT INTO R ANKLE, OPEN APPROACH |
| ICD10_proc | 0YHK31Z | INSERTION OF RADIOACTIVE ELEMENT INTO R ANKLE, PERC APPROACH |
| ICD10_proc | 0YHK41Z | INSERTION OF RADIOACT ELEM INTO R ANKLE, PERC ENDO APPROACH |
| ICD10_proc | 0YHL01Z | INSERTION OF RADIOACTIVE ELEMENT INTO L ANKLE, OPEN APPROACH |
| ICD10_proc | 0YHL31Z | INSERTION OF RADIOACTIVE ELEMENT INTO L ANKLE, PERC APPROACH |
| ICD10_proc | 0YHL41Z | INSERTION OF RADIOACT ELEM INTO L ANKLE, PERC ENDO APPROACH |
| ICD10_proc | 0YHM01Z | INSERTION OF RADIOACTIVE ELEMENT INTO R FOOT, OPEN APPROACH |
| ICD10_proc | 0YHM31Z | INSERTION OF RADIOACTIVE ELEMENT INTO R FOOT, PERC APPROACH |
| ICD10_proc | 0YHM41Z | INSERTION OF RADIOACT ELEM INTO R FOOT, PERC ENDO APPROACH |
| ICD10_proc | 0YHN01Z | INSERTION OF RADIOACTIVE ELEMENT INTO L FOOT, OPEN APPROACH |
| ICD10_proc | 0YHN31Z | INSERTION OF RADIOACTIVE ELEMENT INTO L FOOT, PERC APPROACH |
| ICD10_proc | 0YHN41Z | INSERTION OF RADIOACT ELEM INTO L FOOT, PERC ENDO APPROACH |
| ICD10_proc | 0YH001Z | INSERTION OF RADIOACT ELEM INTO R BUTTOCK, OPEN APPROACH |
| ICD10_proc | 0YH031Z | INSERTION OF RADIOACT ELEM INTO R BUTTOCK, PERC APPROACH |
| ICD10_proc | 0YH041Z | INSERT OF RADIOACT ELEM INTO R BUTTOCK, PERC ENDO APPROACH |
| ICD10_proc | 0YH101Z | INSERTION OF RADIOACT ELEM INTO L BUTTOCK, OPEN APPROACH |
| ICD10_proc | 0YH131Z | INSERTION OF RADIOACT ELEM INTO L BUTTOCK, PERC APPROACH |
| ICD10_proc | 0YH141Z | INSERT OF RADIOACT ELEM INTO L BUTTOCK, PERC ENDO APPROACH |
| ICD10_proc | 0YH501Z | INSERT RADIOACT ELEM IN R INGUINAL REGION, OPEN |
| ICD10_proc | 0YH531Z | INSERT RADIOACT ELEM IN R INGUINAL REGION, PERC |
| ICD10_proc | 0YH541Z | INSERT RADIOACT ELEM IN R INGUINAL REGION, PERC ENDO |
| ICD10_proc | 0YH601Z | INSERT RADIOACT ELEM IN L INGUINAL REGION, OPEN |
| ICD10_proc | 0YH631Z | INSERT RADIOACT ELEM IN L INGUINAL REGION, PERC |
| ICD10_proc | 0YH641Z | INSERT RADIOACT ELEM IN L INGUINAL REGION, PERC ENDO |
| ICD10_proc | 0YH701Z | INSERT OF RADIOACT ELEM INTO R FEMORAL REGION, OPEN APPROACH |
| ICD10_proc | 0YH731Z | INSERT OF RADIOACT ELEM INTO R FEMORAL REGION, PERC APPROACH |
| ICD10_proc | 0YH741Z | INSERT RADIOACT ELEM IN R FEMORAL REGION, PERC ENDO |
| ICD10_proc | 0YH801Z | INSERT OF RADIOACT ELEM INTO L FEMORAL REGION, OPEN APPROACH |
| ICD10_proc | 0YH831Z | INSERT OF RADIOACT ELEM INTO L FEMORAL REGION, PERC APPROACH |
| ICD10_proc | 0YH841Z | INSERT RADIOACT ELEM IN L FEMORAL REGION, PERC ENDO |
| ICD10_proc | 0YH901Z | INSERTION OF RADIOACT ELEM INTO R LOW EXTREM, OPEN APPROACH |
| ICD10_proc | 0YH931Z | INSERTION OF RADIOACT ELEM INTO R LOW EXTREM, PERC APPROACH |
| ICD10_proc | 0YH941Z | INSERT RADIOACT ELEM IN R LOW EXTREM, PERC ENDO |
| ICD10_proc | 00H004Z | INSERTION OF CES 131 COLG IMPLT INTO BRAIN, OPEN APPROACH |
| ICD10_proc | 08H0X1Z | INSERTION OF RADIOACTIVE ELEMENT INTO R EYE, EXTERN APPROACH |
| ICD10_proc | 08H031Z | INSERTION OF RADIOACTIVE ELEMENT INTO R EYE, PERC APPROACH |
| ICD10_proc | 08H1X1Z | INSERTION OF RADIOACTIVE ELEMENT INTO L EYE, EXTERN APPROACH |
| ICD10_proc | 08H131Z | INSERTION OF RADIOACTIVE ELEMENT INTO L EYE, PERC APPROACH |
| ICD10_proc | 0BHK31Z | INSERTION OF RADIOACTIVE ELEMENT INTO R LUNG, PERC APPROACH |
| ICD10_proc | 0BHK41Z | INSERTION OF RADIOACT ELEM INTO R LUNG, PERC ENDO APPROACH |
| ICD10_proc | 0BHK71Z | INSERTION OF RADIOACTIVE ELEMENT INTO R LUNG, VIA OPENING |
| ICD10_proc | 0BHK81Z | INSERTION OF RADIOACTIVE ELEMENT INTO RIGHT LUNG, ENDO |
| ICD10_proc | 0BHL01Z | INSERTION OF RADIOACTIVE ELEMENT INTO L LUNG, OPEN APPROACH |
| ICD10_proc | 0BHL31Z | INSERTION OF RADIOACTIVE ELEMENT INTO L LUNG, PERC APPROACH |
| ICD10_proc | 0BHL41Z | INSERTION OF RADIOACT ELEM INTO L LUNG, PERC ENDO APPROACH |
| ICD10_proc | 0BHL71Z | INSERTION OF RADIOACTIVE ELEMENT INTO LEFT LUNG, VIA OPENING |
| ICD10_proc | 0BHL81Z | INSERTION OF RADIOACTIVE ELEMENT INTO LEFT LUNG, ENDO |
| ICD10_proc | 0BH001Z | INSERT RADIOACT ELEM IN TRACHEOBRONC TREE, OPEN |
| ICD10_proc | 0BH031Z | INSERT RADIOACT ELEM IN TRACHEOBRONC TREE, PERC |
| ICD10_proc | 0BH041Z | INSERT RADIOACT ELEM IN TRACHEOBRONC TREE, PERC ENDO |
| ICD10_proc | 0BH071Z | INSERT OF RADIOACT ELEM INTO TRACHEOBRONC TREE, VIA OPENING |
| ICD10_proc | 0BH081Z | INSERTION OF RADIOACT ELEM INTO TRACHEOBRONC TREE, ENDO |
| ICD10_proc | 0CH7X1Z | INSERTION OF RADIOACT ELEM INTO TONGUE, EXTERN APPROACH |
| ICD10_proc | 0CH701Z | INSERTION OF RADIOACTIVE ELEMENT INTO TONGUE, OPEN APPROACH |
| ICD10_proc | 0CH731Z | INSERTION OF RADIOACTIVE ELEMENT INTO TONGUE, PERC APPROACH |
| ICD10_proc | 0DHP01Z | INSERTION OF RADIOACTIVE ELEMENT INTO RECTUM, OPEN APPROACH |
| ICD10_proc | 0DHP31Z | INSERTION OF RADIOACTIVE ELEMENT INTO RECTUM, PERC APPROACH |
| ICD10_proc | 0DHP41Z | INSERTION OF RADIOACT ELEM INTO RECTUM, PERC ENDO APPROACH |
| ICD10_proc | 0DHP71Z | INSERTION OF RADIOACTIVE ELEMENT INTO RECTUM, VIA OPENING |
| ICD10_proc | 0DHP81Z | INSERTION OF RADIOACTIVE ELEMENT INTO RECTUM, ENDO |
| ICD10_proc | 0DH501Z | INSERTION OF RADIOACTIVE ELEMENT INTO ESOPHAG, OPEN APPROACH |
| ICD10_proc | 0DH531Z | INSERTION OF RADIOACTIVE ELEMENT INTO ESOPHAG, PERC APPROACH |
| ICD10_proc | 0DH541Z | INSERTION OF RADIOACT ELEM INTO ESOPHAG, PERC ENDO APPROACH |
| ICD10_proc | 0DH571Z | INSERTION OF RADIOACTIVE ELEMENT INTO ESOPHAGUS, VIA OPENING |
| ICD10_proc | 0DH581Z | INSERTION OF RADIOACTIVE ELEMENT INTO ESOPHAGUS, ENDO |
| ICD10_proc | 0FHB01Z | INSERT OF RADIOACT ELEM INTO HEPATOBIL DUCT, OPEN APPROACH |
| ICD10_proc | 0FHB31Z | INSERT OF RADIOACT ELEM INTO HEPATOBIL DUCT, PERC APPROACH |
| ICD10_proc | 0FHB41Z | INSERT RADIOACT ELEM IN HEPATOBIL DUCT, PERC ENDO |
| ICD10_proc | 0FHB71Z | INSERTION OF RADIOACT ELEM INTO HEPATOBIL DUCT, VIA OPENING |
| ICD10_proc | 0FHB81Z | INSERTION OF RADIOACTIVE ELEMENT INTO HEPATOBIL DUCT, ENDO |
| ICD10_proc | 0FHD01Z | INSERTION OF RADIOACT ELEM INTO PANCREAT DUCT, OPEN APPROACH |
| ICD10_proc | 0FHD31Z | INSERTION OF RADIOACT ELEM INTO PANCREAT DUCT, PERC APPROACH |
| ICD10_proc | 0FHD41Z | INSERT RADIOACT ELEM IN PANCREAT DUCT, PERC ENDO |
| ICD10_proc | 0FHD71Z | INSERTION OF RADIOACT ELEM INTO PANCREAT DUCT, VIA OPENING |
| ICD10_proc | 0FHD81Z | INSERTION OF RADIOACTIVE ELEMENT INTO PANCREATIC DUCT, ENDO |
| ICD10_proc | 0HHTX1Z | INSERTION OF RADIOACT ELEM INTO R BREAST, EXTERN APPROACH |
| ICD10_proc | 0HHT01Z | INSERTION OF RADIOACT ELEM INTO R BREAST, OPEN APPROACH |
| ICD10_proc | 0HHT31Z | INSERTION OF RADIOACT ELEM INTO R BREAST, PERC APPROACH |
| ICD10_proc | 0HHT71Z | INSERTION OF RADIOACTIVE ELEMENT INTO R BREAST, VIA OPENING |
| ICD10_proc | 0HHT81Z | INSERTION OF RADIOACTIVE ELEMENT INTO RIGHT BREAST, ENDO |
| ICD10_proc | 0HHUX1Z | INSERTION OF RADIOACT ELEM INTO L BREAST, EXTERN APPROACH |
| ICD10_proc | 0HHU01Z | INSERTION OF RADIOACT ELEM INTO L BREAST, OPEN APPROACH |
| ICD10_proc | 0HHU31Z | INSERTION OF RADIOACT ELEM INTO L BREAST, PERC APPROACH |
| ICD10_proc | 0HHU71Z | INSERTION OF RADIOACTIVE ELEMENT INTO L BREAST, VIA OPENING |
| ICD10_proc | 0HHU81Z | INSERTION OF RADIOACTIVE ELEMENT INTO LEFT BREAST, ENDO |
| ICD10_proc | 0HHVX1Z | INSERTION OF RADIOACT ELEM INTO BI BREAST, EXTERN APPROACH |
| ICD10_proc | 0HHV01Z | INSERTION OF RADIOACT ELEM INTO BI BREAST, OPEN APPROACH |
| ICD10_proc | 0HHV31Z | INSERTION OF RADIOACT ELEM INTO BI BREAST, PERC APPROACH |
| ICD10_proc | 0HHV71Z | INSERTION OF RADIOACTIVE ELEMENT INTO BI BREAST, VIA OPENING |
| ICD10_proc | 0HHV81Z | INSERTION OF RADIOACTIVE ELEMENT INTO BILATERAL BREAST, ENDO |
| ICD10_proc | 0HHWX1Z | INSERTION OF RADIOACT ELEM INTO R NIPPLE, EXTERN APPROACH |
| ICD10_proc | 0HHW01Z | INSERTION OF RADIOACT ELEM INTO R NIPPLE, OPEN APPROACH |
| ICD10_proc | 0HHW31Z | INSERTION OF RADIOACT ELEM INTO R NIPPLE, PERC APPROACH |
| ICD10_proc | 0HHW71Z | INSERTION OF RADIOACTIVE ELEMENT INTO R NIPPLE, VIA OPENING |
| ICD10_proc | 0HHW81Z | INSERTION OF RADIOACTIVE ELEMENT INTO RIGHT NIPPLE, ENDO |
| ICD10_proc | 0HHXX1Z | INSERTION OF RADIOACT ELEM INTO L NIPPLE, EXTERN APPROACH |
| ICD10_proc | 0HHX01Z | INSERTION OF RADIOACT ELEM INTO L NIPPLE, OPEN APPROACH |
| ICD10_proc | 0HHX31Z | INSERTION OF RADIOACT ELEM INTO L NIPPLE, PERC APPROACH |
| ICD10_proc | 0HHX71Z | INSERTION OF RADIOACTIVE ELEMENT INTO L NIPPLE, VIA OPENING |
| ICD10_proc | 0HHX81Z | INSERTION OF RADIOACTIVE ELEMENT INTO LEFT NIPPLE, ENDO |
| ICD10_proc | 0JHS01Z | INSERT RADIOACT ELEM IN HEAD & NECK SUBCU/FASCIA, OPEN |
| ICD10_proc | 0JHS31Z | INSERT RADIOACT ELEM IN HEAD & NECK SUBCU/FASCIA, PERC |
| ICD10_proc | 0JHT01Z | INSERT RADIOACT ELEM IN TRUNK SUBCU/FASCIA, OPEN |
| ICD10_proc | 0JHT31Z | INSERT RADIOACT ELEM IN TRUNK SUBCU/FASCIA, PERC |
| ICD10_proc | 0JHV01Z | INSERT RADIOACT ELEM IN UP EXTREM SUBCU/FASCIA, OPEN |
| ICD10_proc | 0JHV31Z | INSERT RADIOACT ELEM IN UP EXTREM SUBCU/FASCIA, PERC |
| ICD10_proc | 0JHW01Z | INSERT RADIOACT ELEM IN LOW EXTREM SUBCU/FASCIA, OPEN |
| ICD10_proc | 0JHW31Z | INSERT RADIOACT ELEM IN LOW EXTREM SUBCU/FASCIA, PERC |
| ICD10_proc | CW7GGZZ | SYS NUCL MED THERAPY OF THYROID USING IODINE 131 |
| ICD10_proc | CW7GYZZ | SYS NUCL MED THERAPY OF THYROID USING OTH RADIONUCLIDE |
| ICD10_proc | CW7NGZZ | SYS NUCL MED THERAPY OF WHOLE BODY USING IODINE 131 |
| ICD10_proc | CW7NNZZ | SYS NUCL MED THERAPY OF WHOLE BODY USING PHOSPHORUS 32 |
| ICD10_proc | CW7NPZZ | SYS NUCL MED THERAPY OF WHOLE BODY USING STRONTIUM 89 |
| ICD10_proc | CW7NYZZ | SYS NUCL MED THERAPY OF WHOLE BODY USING OTH RADIONUCLIDE |
| ICD10_proc | CW7N8ZZ | SYS NUCL MED THERAPY OF WHOLE BODY USING SAMARIUM 153 |
| ICD10_proc | CW7YYZZ | SYS NUCL MED THERAPY MULT ANATOM REGION W OTH RADIONUCLIDE |
| ICD10_proc | CW70NZZ | SYSTEMIC NUCLEAR MEDICINE THERAPY OF ABD USING PHOSPHORUS 32 |
| ICD10_proc | CW70YZZ | SYS NUCL MED THERAPY OF ABD USING OTH RADIONUCLIDE |
| ICD10_proc | CW73NZZ | SYS NUCL MED THERAPY OF CHEST USING PHOSPHORUS 32 |
| ICD10_proc | CW73YZZ | SYS NUCL MED THERAPY OF CHEST USING OTH RADIONUCLIDE |
| ICD10_proc | DWY5GDZ | ISOTOPE ADMINISTRATION TO WHOLE BODY USING IODINE 131 |
| ICD10_proc | DWY5GFZ | ISOTOPE ADMINISTRATION TO WHOLE BODY USING PHOSPHORUS 32 |
| ICD10_proc | DWY5GGZ | ISOTOPE ADMINISTRATION TO WHOLE BODY USING STRONTIUM 89 |
| ICD10_proc | DWY5GHZ | ISOTOPE ADMINISTRATION TO WHOLE BODY USING STRONTIUM 90 |
| ICD10_proc | DWY5GYZ | ISOTOPE ADMINISTRATION TO WHOLE BODY USING OTHER ISOTOPE |
| ICD10_proc | DBY0FZZ | PLAQUE RADIATION OF TRACHEA |
| ICD10_proc | DBY1FZZ | PLAQUE RADIATION OF BRONCHUS |
| ICD10_proc | DBY2FZZ | PLAQUE RADIATION OF LUNG |
| ICD10_proc | DBY5FZZ | PLAQUE RADIATION OF PLEURA |
| ICD10_proc | DBY6FZZ | PLAQUE RADIATION OF MEDIASTINUM |
| ICD10_proc | DBY7FZZ | PLAQUE RADIATION OF CHEST WALL |
| ICD10_proc | DBY8FZZ | PLAQUE RADIATION OF DIAPHRAGM |
| ICD10_proc | DDY0FZZ | PLAQUE RADIATION OF ESOPHAGUS |
| ICD10_proc | DDY1CZZ | INTRAOPERATIVE RADIATION THERAPY (IORT) OF STOMACH |
| ICD10_proc | DDY1FZZ | PLAQUE RADIATION OF STOMACH |
| ICD10_proc | DDY2CZZ | INTRAOPERATIVE RADIATION THERAPY (IORT) OF DUODENUM |
| ICD10_proc | DDY2FZZ | PLAQUE RADIATION OF DUODENUM |
| ICD10_proc | DDY3CZZ | INTRAOPERATIVE RADIATION THERAPY (IORT) OF JEJUNUM |
| ICD10_proc | DDY3FZZ | PLAQUE RADIATION OF JEJUNUM |
| ICD10_proc | DDY4CZZ | INTRAOPERATIVE RADIATION THERAPY (IORT) OF ILEUM |
| ICD10_proc | DDY4FZZ | PLAQUE RADIATION OF ILEUM |
| ICD10_proc | DDY5CZZ | INTRAOPERATIVE RADIATION THERAPY (IORT) OF COLON |
| ICD10_proc | DDY5FZZ | PLAQUE RADIATION OF COLON |
| ICD10_proc | DDY7CZZ | INTRAOPERATIVE RADIATION THERAPY (IORT) OF RECTUM |
| ICD10_proc | DDY7FZZ | PLAQUE RADIATION OF RECTUM |
| ICD10_proc | DDY8CZZ | INTRAOPERATIVE RADIATION THERAPY (IORT) OF ANUS |
| ICD10_proc | DDY8FZZ | PLAQUE RADIATION OF ANUS |
| ICD10_proc | DFY0CZZ | INTRAOPERATIVE RADIATION THERAPY (IORT) OF LIVER |
| ICD10_proc | DFY0FZZ | PLAQUE RADIATION OF LIVER |
| ICD10_proc | DFY1CZZ | INTRAOPERATIVE RADIATION THERAPY (IORT) OF GALLBLADDER |
| ICD10_proc | DFY1FZZ | PLAQUE RADIATION OF GALLBLADDER |
| ICD10_proc | DFY2CZZ | INTRAOPERATIVE RADIATION THERAPY (IORT) OF BILE DUCTS |
| ICD10_proc | DFY2FZZ | PLAQUE RADIATION OF BILE DUCTS |
| ICD10_proc | DFY3CZZ | INTRAOPERATIVE RADIATION THERAPY (IORT) OF PANCREAS |
| ICD10_proc | DFY3FZZ | PLAQUE RADIATION OF PANCREAS |
| ICD10_proc | DGY0FZZ | PLAQUE RADIATION OF PITUITARY GLAND |
| ICD10_proc | DGY1FZZ | PLAQUE RADIATION OF PINEAL BODY |
| ICD10_proc | DGY2FZZ | PLAQUE RADIATION OF ADRENAL GLANDS |
| ICD10_proc | DGY4FZZ | PLAQUE RADIATION OF PARATHYROID GLANDS |
| ICD10_proc | DGY5FZZ | PLAQUE RADIATION OF THYROID |
| ICD10_proc | DHYBFZZ | PLAQUE RADIATION OF LEG SKIN |
| ICD10_proc | DHYCFZZ | PLAQUE RADIATION OF FOOT SKIN |
| ICD10_proc | DHY2FZZ | PLAQUE RADIATION OF FACE SKIN |
| ICD10_proc | DHY3FZZ | PLAQUE RADIATION OF NECK SKIN |
| ICD10_proc | DHY4FZZ | PLAQUE RADIATION OF ARM SKIN |
| ICD10_proc | DHY5FZZ | PLAQUE RADIATION OF HAND SKIN |
| ICD10_proc | DHY6FZZ | PLAQUE RADIATION OF CHEST SKIN |
| ICD10_proc | DHY7FZZ | PLAQUE RADIATION OF BACK SKIN |
| ICD10_proc | DHY8FZZ | PLAQUE RADIATION OF ABDOMEN SKIN |
| ICD10_proc | DHY9FZZ | PLAQUE RADIATION OF BUTTOCK SKIN |
| ICD10_proc | DMY0FZZ | PLAQUE RADIATION OF LEFT BREAST |
| ICD10_proc | DMY1FZZ | PLAQUE RADIATION OF RIGHT BREAST |
| ICD10_proc | DPYBFZZ | PLAQUE RADIATION OF TIBIA/FIBULA |
| ICD10_proc | DPYCFZZ | PLAQUE RADIATION OF OTHER BONE |
| ICD10_proc | DPY0FZZ | PLAQUE RADIATION OF SKULL |
| ICD10_proc | DPY2FZZ | PLAQUE RADIATION OF MAXILLA |
| ICD10_proc | DPY3FZZ | PLAQUE RADIATION OF MANDIBLE |
| ICD10_proc | DPY4FZZ | PLAQUE RADIATION OF STERNUM |
| ICD10_proc | DPY5FZZ | PLAQUE RADIATION OF RIB(S) |
| ICD10_proc | DPY6FZZ | PLAQUE RADIATION OF HUMERUS |
| ICD10_proc | DPY7FZZ | PLAQUE RADIATION OF RADIUS/ULNA |
| ICD10_proc | DPY8FZZ | PLAQUE RADIATION OF PELVIC BONES |
| ICD10_proc | DPY9FZZ | PLAQUE RADIATION OF FEMUR |
| ICD10_proc | DTY0CZZ | INTRAOPERATIVE RADIATION THERAPY (IORT) OF KIDNEY |
| ICD10_proc | DTY0FZZ | PLAQUE RADIATION OF KIDNEY |
| ICD10_proc | DTY1CZZ | INTRAOPERATIVE RADIATION THERAPY (IORT) OF URETER |
| ICD10_proc | DTY1FZZ | PLAQUE RADIATION OF URETER |
| ICD10_proc | DTY2CZZ | INTRAOPERATIVE RADIATION THERAPY (IORT) OF BLADDER |
| ICD10_proc | DTY2FZZ | PLAQUE RADIATION OF BLADDER |
| ICD10_proc | DTY3CZZ | INTRAOPERATIVE RADIATION THERAPY (IORT) OF URETHRA |
| ICD10_proc | DTY3FZZ | PLAQUE RADIATION OF URETHRA |
| ICD10_proc | DUY0CZZ | INTRAOPERATIVE RADIATION THERAPY (IORT) OF OVARY |
| ICD10_proc | DUY0FZZ | PLAQUE RADIATION OF OVARY |
| ICD10_proc | DUY1CZZ | INTRAOPERATIVE RADIATION THERAPY (IORT) OF CERVIX |
| ICD10_proc | DUY1FZZ | PLAQUE RADIATION OF CERVIX |
| ICD10_proc | DUY2CZZ | INTRAOPERATIVE RADIATION THERAPY (IORT) OF UTERUS |
| ICD10_proc | DUY2FZZ | PLAQUE RADIATION OF UTERUS |
| ICD10_proc | DVY0CZZ | INTRAOPERATIVE RADIATION THERAPY (IORT) OF PROSTATE |
| ICD10_proc | DVY0FZZ | PLAQUE RADIATION OF PROSTATE |
| ICD10_proc | DVY1FZZ | PLAQUE RADIATION OF TESTIS |
| ICD10_proc | DWY1FZZ | PLAQUE RADIATION OF HEAD AND NECK |
| ICD10_proc | DWY2FZZ | PLAQUE RADIATION OF CHEST |
| ICD10_proc | DWY3FZZ | PLAQUE RADIATION OF ABDOMEN |
| ICD10_proc | DWY4FZZ | PLAQUE RADIATION OF HEMIBODY |
| ICD10_proc | DWY5FZZ | PLAQUE RADIATION OF WHOLE BODY |
| ICD10_proc | DWY6FZZ | PLAQUE RADIATION OF PELVIC REGION |
| ICD10_proc | D0Y0FZZ | PLAQUE RADIATION OF BRAIN |
| ICD10_proc | D0Y1FZZ | PLAQUE RADIATION OF BRAIN STEM |
| ICD10_proc | D0Y6FZZ | PLAQUE RADIATION OF SPINAL CORD |
| ICD10_proc | D0Y7FZZ | PLAQUE RADIATION OF PERIPHERAL NERVE |
| ICD10_proc | D7Y0FZZ | PLAQUE RADIATION OF BONE MARROW |
| ICD10_proc | D7Y1FZZ | PLAQUE RADIATION OF THYMUS |
| ICD10_proc | D7Y2FZZ | PLAQUE RADIATION OF SPLEEN |
| ICD10_proc | D7Y3FZZ | PLAQUE RADIATION OF NECK LYMPHATICS |
| ICD10_proc | D7Y4FZZ | PLAQUE RADIATION OF AXILLARY LYMPHATICS |
| ICD10_proc | D7Y5FZZ | PLAQUE RADIATION OF THORAX LYMPHATICS |
| ICD10_proc | D7Y6FZZ | PLAQUE RADIATION OF ABDOMEN LYMPHATICS |
| ICD10_proc | D7Y7FZZ | PLAQUE RADIATION OF PELVIS LYMPHATICS |
| ICD10_proc | D7Y8FZZ | PLAQUE RADIATION OF INGUINAL LYMPHATICS |
| ICD10_proc | D8Y0FZZ | PLAQUE RADIATION OF EYE |
| ICD10_proc | D9YBCZZ | INTRAOPERATIVE RADIATION THERAPY (IORT) OF LARYNX |
| ICD10_proc | D9YBFZZ | PLAQUE RADIATION OF LARYNX |
| ICD10_proc | D9YCCZZ | INTRAOPERATIVE RADIATION THERAPY (IORT) OF PHARYNX |
| ICD10_proc | D9YCFZZ | PLAQUE RADIATION OF PHARYNX |
| ICD10_proc | D9YDCZZ | INTRAOPERATIVE RADIATION THERAPY (IORT) OF NASOPHARYNX |
| ICD10_proc | D9YDFZZ | PLAQUE RADIATION OF NASOPHARYNX |
| ICD10_proc | D9Y0FZZ | PLAQUE RADIATION OF EAR |
| ICD10_proc | D9Y1FZZ | PLAQUE RADIATION OF NOSE |
| ICD10_proc | D9Y4CZZ | INTRAOPERATIVE RADIATION THERAPY (IORT) OF MOUTH |
| ICD10_proc | D9Y4FZZ | PLAQUE RADIATION OF MOUTH |
| ICD10_proc | D9Y5FZZ | PLAQUE RADIATION OF TONGUE |
| ICD10_proc | D9Y6FZZ | PLAQUE RADIATION OF SALIVARY GLANDS |
| ICD10_proc | D9Y7FZZ | PLAQUE RADIATION OF SINUSES |
| ICD10_proc | D9Y8FZZ | PLAQUE RADIATION OF HARD PALATE |
| ICD10_proc | D9Y9FZZ | PLAQUE RADIATION OF SOFT PALATE |
| ICD10_proc | DB003Z0 | BEAM RADIATION OF TRACHEA USING ELECTRONS, INTRAOPERATIVE |
| ICD10_proc | DB013Z0 | BEAM RADIATION OF BRONCHUS USING ELECTRONS, INTRAOPERATIVE |
| ICD10_proc | DB023Z0 | BEAM RADIATION OF LUNG USING ELECTRONS, INTRAOPERATIVE |
| ICD10_proc | DB053Z0 | BEAM RADIATION OF PLEURA USING ELECTRONS, INTRAOPERATIVE |
| ICD10_proc | DB063Z0 | BEAM RADIATION OF MEDIASTINUM USING ELECTRONS, INTRAOP |
| ICD10_proc | DB073Z0 | BEAM RADIATION OF CHEST WALL USING ELECTRONS, INTRAOPERATIVE |
| ICD10_proc | DB083Z0 | BEAM RADIATION OF DIAPHRAGM USING ELECTRONS, INTRAOPERATIVE |
| ICD10_proc | DD003Z0 | BEAM RADIATION OF ESOPHAGUS USING ELECTRONS, INTRAOPERATIVE |
| ICD10_proc | DD013Z0 | BEAM RADIATION OF STOMACH USING ELECTRONS, INTRAOPERATIVE |
| ICD10_proc | DD023Z0 | BEAM RADIATION OF DUODENUM USING ELECTRONS, INTRAOPERATIVE |
| ICD10_proc | DD033Z0 | BEAM RADIATION OF JEJUNUM USING ELECTRONS, INTRAOPERATIVE |
| ICD10_proc | DD043Z0 | BEAM RADIATION OF ILEUM USING ELECTRONS, INTRAOPERATIVE |
| ICD10_proc | DD053Z0 | BEAM RADIATION OF COLON USING ELECTRONS, INTRAOPERATIVE |
| ICD10_proc | DD073Z0 | BEAM RADIATION OF RECTUM USING ELECTRONS, INTRAOPERATIVE |
| ICD10_proc | DF003Z0 | BEAM RADIATION OF LIVER USING ELECTRONS, INTRAOPERATIVE |
| ICD10_proc | DF013Z0 | BEAM RADIATION OF GALLBLADDER USING ELECTRONS, INTRAOP |
| ICD10_proc | DF023Z0 | BEAM RADIATION OF BILE DUCTS USING ELECTRONS, INTRAOPERATIVE |
| ICD10_proc | DF033Z0 | BEAM RADIATION OF PANCREAS USING ELECTRONS, INTRAOPERATIVE |
| ICD10_proc | DG003Z0 | BEAM RADIATION OF PITUITARY GLAND USING ELECTRONS, INTRAOP |
| ICD10_proc | DG013Z0 | BEAM RADIATION OF PINEAL BODY USING ELECTRONS, INTRAOP |
| ICD10_proc | DG023Z0 | BEAM RADIATION OF ADRENAL GLANDS USING ELECTRONS, INTRAOP |
| ICD10_proc | DG043Z0 | BEAM RADIATION PARATHYROID GLANDS W ELECTRONS, INTRAOP |
| ICD10_proc | DG053Z0 | BEAM RADIATION OF THYROID USING ELECTRONS, INTRAOPERATIVE |
| ICD10_proc | DH0B3Z0 | BEAM RADIATION OF LEG SKIN USING ELECTRONS, INTRAOPERATIVE |
| ICD10_proc | DH023Z0 | BEAM RADIATION OF FACE SKIN USING ELECTRONS, INTRAOPERATIVE |
| ICD10_proc | DH033Z0 | BEAM RADIATION OF NECK SKIN USING ELECTRONS, INTRAOPERATIVE |
| ICD10_proc | DH043Z0 | BEAM RADIATION OF ARM SKIN USING ELECTRONS, INTRAOPERATIVE |
| ICD10_proc | DH063Z0 | BEAM RADIATION OF CHEST SKIN USING ELECTRONS, INTRAOPERATIVE |
| ICD10_proc | DH073Z0 | BEAM RADIATION OF BACK SKIN USING ELECTRONS, INTRAOPERATIVE |
| ICD10_proc | DH083Z0 | BEAM RADIATION OF ABDOMEN SKIN USING ELECTRONS, INTRAOP |
| ICD10_proc | DH093Z0 | BEAM RADIATION OF BUTTOCK SKIN USING ELECTRONS, INTRAOP |
| ICD10_proc | DM003Z0 | BEAM RADIATION OF LEFT BREAST USING ELECTRONS, INTRAOP |
| ICD10_proc | DM013Z0 | BEAM RADIATION OF RIGHT BREAST USING ELECTRONS, INTRAOP |
| ICD10_proc | DP0B3Z0 | BEAM RADIATION OF TIBIA/FIBULA USING ELECTRONS, INTRAOP |
| ICD10_proc | DP0C3Z0 | BEAM RADIATION OF OTHER BONE USING ELECTRONS, INTRAOPERATIVE |
| ICD10_proc | DP003Z0 | BEAM RADIATION OF SKULL USING ELECTRONS, INTRAOPERATIVE |
| ICD10_proc | DP023Z0 | BEAM RADIATION OF MAXILLA USING ELECTRONS, INTRAOPERATIVE |
| ICD10_proc | DP033Z0 | BEAM RADIATION OF MANDIBLE USING ELECTRONS, INTRAOPERATIVE |
| ICD10_proc | DP043Z0 | BEAM RADIATION OF STERNUM USING ELECTRONS, INTRAOPERATIVE |
| ICD10_proc | DP053Z0 | BEAM RADIATION OF RIB(S) USING ELECTRONS, INTRAOPERATIVE |
| ICD10_proc | DP063Z0 | BEAM RADIATION OF HUMERUS USING ELECTRONS, INTRAOPERATIVE |
| ICD10_proc | DP073Z0 | BEAM RADIATION OF RADIUS/ULNA USING ELECTRONS, INTRAOP |
| ICD10_proc | DP083Z0 | BEAM RADIATION OF PELVIC BONES USING ELECTRONS, INTRAOP |
| ICD10_proc | DP093Z0 | BEAM RADIATION OF FEMUR USING ELECTRONS, INTRAOPERATIVE |
| ICD10_proc | DT003Z0 | BEAM RADIATION OF KIDNEY USING ELECTRONS, INTRAOPERATIVE |
| ICD10_proc | DT013Z0 | BEAM RADIATION OF URETER USING ELECTRONS, INTRAOPERATIVE |
| ICD10_proc | DT023Z0 | BEAM RADIATION OF BLADDER USING ELECTRONS, INTRAOPERATIVE |
| ICD10_proc | DT033Z0 | BEAM RADIATION OF URETHRA USING ELECTRONS, INTRAOPERATIVE |
| ICD10_proc | DU003Z0 | BEAM RADIATION OF OVARY USING ELECTRONS, INTRAOPERATIVE |
| ICD10_proc | DU013Z0 | BEAM RADIATION OF CERVIX USING ELECTRONS, INTRAOPERATIVE |
| ICD10_proc | DU023Z0 | BEAM RADIATION OF UTERUS USING ELECTRONS, INTRAOPERATIVE |
| ICD10_proc | DV003Z0 | BEAM RADIATION OF PROSTATE USING ELECTRONS, INTRAOPERATIVE |
| ICD10_proc | DV013Z0 | BEAM RADIATION OF TESTIS USING ELECTRONS, INTRAOPERATIVE |
| ICD10_proc | DW013Z0 | BEAM RADIATION OF HEAD AND NECK USING ELECTRONS, INTRAOP |
| ICD10_proc | DW023Z0 | BEAM RADIATION OF CHEST USING ELECTRONS, INTRAOPERATIVE |
| ICD10_proc | DW033Z0 | BEAM RADIATION OF ABDOMEN USING ELECTRONS, INTRAOPERATIVE |
| ICD10_proc | DW043Z0 | BEAM RADIATION OF HEMIBODY USING ELECTRONS, INTRAOPERATIVE |
| ICD10_proc | DW053Z0 | BEAM RADIATION OF WHOLE BODY USING ELECTRONS, INTRAOPERATIVE |
| ICD10_proc | DW063Z0 | BEAM RADIATION OF PELVIC REGION USING ELECTRONS, INTRAOP |
| ICD10_proc | D0003Z0 | BEAM RADIATION OF BRAIN USING ELECTRONS, INTRAOPERATIVE |
| ICD10_proc | D0013Z0 | BEAM RADIATION OF BRAIN STEM USING ELECTRONS, INTRAOPERATIVE |
| ICD10_proc | D0063Z0 | BEAM RADIATION OF SPINAL CORD USING ELECTRONS, INTRAOP |
| ICD10_proc | D0073Z0 | BEAM RADIATION OF PERIPHERAL NERVE USING ELECTRONS, INTRAOP |
| ICD10_proc | D7003Z0 | BEAM RADIATION OF BONE MARROW USING ELECTRONS, INTRAOP |
| ICD10_proc | D7013Z0 | BEAM RADIATION OF THYMUS USING ELECTRONS, INTRAOPERATIVE |
| ICD10_proc | D7023Z0 | BEAM RADIATION OF SPLEEN USING ELECTRONS, INTRAOPERATIVE |
| ICD10_proc | D7033Z0 | BEAM RADIATION OF NECK LYMPHATICS USING ELECTRONS, INTRAOP |
| ICD10_proc | D7043Z0 | BEAM RADIATION OF AXILLA LYMPH USING ELECTRONS, INTRAOP |
| ICD10_proc | D7053Z0 | BEAM RADIATION OF THORAX LYMPHATICS USING ELECTRONS, INTRAOP |
| ICD10_proc | D7063Z0 | BEAM RADIATION OF ABD LYMPH USING ELECTRONS, INTRAOP |
| ICD10_proc | D7073Z0 | BEAM RADIATION OF PELVIS LYMPHATICS USING ELECTRONS, INTRAOP |
| ICD10_proc | D7083Z0 | BEAM RADIATION OF INGUINAL LYMPH USING ELECTRONS, INTRAOP |
| ICD10_proc | D8003Z0 | BEAM RADIATION OF EYE USING ELECTRONS, INTRAOPERATIVE |
| ICD10_proc | D90B3Z0 | BEAM RADIATION OF LARYNX USING ELECTRONS, INTRAOPERATIVE |
| ICD10_proc | D90D3Z0 | BEAM RADIATION OF NASOPHARYNX USING ELECTRONS, INTRAOP |
| ICD10_proc | D90F3Z0 | BEAM RADIATION OF OROPHARYNX USING ELECTRONS, INTRAOPERATIVE |
| ICD10_proc | D9003Z0 | BEAM RADIATION OF EAR USING ELECTRONS, INTRAOPERATIVE |
| ICD10_proc | D9013Z0 | BEAM RADIATION OF NOSE USING ELECTRONS, INTRAOPERATIVE |
| ICD10_proc | D9033Z0 | BEAM RADIATION OF HYPOPHARYNX USING ELECTRONS, INTRAOP |
| ICD10_proc | D9043Z0 | BEAM RADIATION OF MOUTH USING ELECTRONS, INTRAOPERATIVE |
| ICD10_proc | D9053Z0 | BEAM RADIATION OF TONGUE USING ELECTRONS, INTRAOPERATIVE |
| ICD10_proc | D9063Z0 | BEAM RADIATION OF SALIVARY GLANDS USING ELECTRONS, INTRAOP |
| ICD10_proc | D9073Z0 | BEAM RADIATION OF SINUSES USING ELECTRONS, INTRAOPERATIVE |
| ICD10_proc | D9083Z0 | BEAM RADIATION OF HARD PALATE USING ELECTRONS, INTRAOP |
| ICD10_proc | D9093Z0 | BEAM RADIATION OF SOFT PALATE USING ELECTRONS, INTRAOP |
| ICD10_dx | Z51.0 | ENCOUNTER FOR ANTINEOPLASTIC RADIATION THERAPY |
| ICD9_proc | 92.20 | Infusion of liquid brachytherapy radioisotope |
| ICD10_proc | 3E0B304 | INTRODUCTION OF LIQUID BRACHY INTO EAR, PERC APPROACH |
| ICD10_proc | 3E0B704 | INTRODUCTION OF LIQUID BRACHY INTO EAR, VIA OPENING |
| ICD10_proc | 3E0BX04 | INTRODUCTION OF LIQUID BRACHY INTO EAR, EXTERN APPROACH |
| ICD10_proc | 3E0C304 | INTRODUCTION OF LIQUID BRACHY INTO EYE, PERC APPROACH |
| ICD10_proc | 3E0C704 | INTRODUCTION OF LIQUID BRACHY INTO EYE, VIA OPENING |
| ICD10_proc | 3E0CX04 | INTRODUCTION OF LIQUID BRACHY INTO EYE, EXTERN APPROACH |
| ICD10_proc | 3E0D304 | INTRODUCTION OF LIQUID BRACHY INTO MOUTH/PHAR, PERC APPROACH |
| ICD10_proc | 3E0D704 | INTRODUCTION OF LIQUID BRACHY INTO MOUTH/PHAR, VIA OPENING |
| ICD10_proc | 3E0DX04 | INTRODUCE OF LIQUID BRACHY INTO MOUTH/PHAR, EXTERN APPROACH |
| ICD10_proc | 3E0E304 | INTRODUCTION OF LIQUID BRACHY INTO POC, PERC APPROACH |
| ICD10_proc | 3E0E704 | INTRODUCTION OF LIQUID BRACHY INTO POC, VIA OPENING |
| ICD10_proc | 3E0E804 | INTRODUCTION OF LIQUID BRACHY INTO POC, ENDO |
| ICD10_proc | 3E0F304 | INTRODUCTION OF LIQUID BRACHY INTO RESP TRACT, PERC APPROACH |
| ICD10_proc | 3E0F704 | INTRODUCTION OF LIQUID BRACHY INTO RESP TRACT, VIA OPENING |
| ICD10_proc | 3E0F804 | INTRODUCTION OF LIQUID BRACHY INTO RESP TRACT, ENDO |
| ICD10_proc | 3E0G304 | INTRODUCTION OF LIQUID BRACHY INTO UP GI, PERC APPROACH |
| ICD10_proc | 3E0G704 | INTRODUCTION OF LIQUID BRACHY INTO UP GI, VIA OPENING |
| ICD10_proc | 3E0G804 | INTRODUCTION OF LIQUID BRACHY INTO UP GI, ENDO |
| ICD10_proc | 3E0H304 | INTRODUCTION OF LIQUID BRACHY INTO LOW GI, PERC APPROACH |
| ICD10_proc | 3E0H704 | INTRODUCTION OF LIQUID BRACHY INTO LOW GI, VIA OPENING |
| ICD10_proc | 3E0H804 | INTRODUCTION OF LIQUID BRACHY INTO LOW GI, ENDO |
| ICD10_proc | 3E0J304 | INTRODUCE LIQUID BRACHY IN BIL/PANC TRACT, PERC |
| ICD10_proc | 3E0J704 | INTRODUCE OF LIQUID BRACHY INTO BIL/PANC TRACT, VIA OPENING |
| ICD10_proc | 3E0J804 | INTRODUCTION OF LIQUID BRACHY INTO BIL/PANC TRACT, ENDO |
| ICD10_proc | 3E0K304 | INTRODUCTION OF LIQUID BRACHY INTO GU TRACT, PERC APPROACH |
| ICD10_proc | 3E0K704 | INTRODUCTION OF LIQUID BRACHY INTO GU TRACT, VIA OPENING |
| ICD10_proc | 3E0K804 | INTRODUCTION OF LIQUID BRACHY INTO GU TRACT, ENDO |
| ICD10_proc | 3E0L304 | INTRODUCE OF LIQUID BRACHY INTO PLEURAL CAV, PERC APPROACH |
| ICD10_proc | 3E0L704 | INTRODUCTION OF LIQUID BRACHY INTO PLEURAL CAV, VIA OPENING |
| ICD10_proc | 3E0M304 | INTRODUCE OF LIQUID BRACHY INTO PERITON CAV, PERC APPROACH |
| ICD10_proc | 3E0M704 | INTRODUCTION OF LIQUID BRACHY INTO PERITON CAV, VIA OPENING |
| ICD10_proc | 3E0N304 | INTRODUCE OF LIQUID BRACHY INTO MALE REPROD, PERC APPROACH |
| ICD10_proc | 3E0N704 | INTRODUCTION OF LIQUID BRACHY INTO MALE REPROD, VIA OPENING |
| ICD10_proc | 3E0N804 | INTRODUCTION OF LIQUID BRACHY INTO MALE REPROD, ENDO |
| ICD10_proc | 3E0P304 | INTRODUCTION OF LIQUID BRACHY INTO FEM REPROD, PERC APPROACH |
| ICD10_proc | 3E0P704 | INTRODUCTION OF LIQUID BRACHY INTO FEM REPROD, VIA OPENING |
| ICD10_proc | 3E0P804 | INTRODUCTION OF LIQUID BRACHY INTO FEM REPROD, ENDO |
| ICD10_proc | 3E0Q004 | INTRODUCE LIQUID BRACHY IN CRAN CAV/BRAIN, OPEN |
| ICD10_proc | 3E0Q304 | INTRODUCE LIQUID BRACHY IN CRAN CAV/BRAIN, PERC |
| ICD10_proc | 3E0Q704 | INTRODUCE OF LIQUID BRACHY INTO CRAN CAV/BRAIN, VIA OPENING |
| ICD10_proc | 3E0R304 | INTRODUCE OF LIQUID BRACHY INTO SPINAL CANAL, PERC APPROACH |
| ICD10_proc | 3E0S304 | INTRODUCE LIQUID BRACHY IN EPIDURAL SPACE, PERC |
| ICD10_proc | 3E0U304 | INTRODUCTION OF LIQUID BRACHY INTO JOINT, PERC APPROACH |
| ICD10_proc | 3E0Y304 | INTRODUCE OF LIQUID BRACHY INTO PERICARD CAV, PERC APPROACH |
| ICD10_proc | 3E0Y704 | INTRODUCTION OF LIQUID BRACHY INTO PERICARD CAV, VIA OPENING |

Supplementary Figure 1: Distribution of Center Volume of Neurosurgical Procedure from 2007-2019.

Note: Outliers are not shown. Of the 810 providers - 143 had more than 42 resections (across 2007 – 2019) and 66 had more than 25 biopsies (across 2007 – 2019).


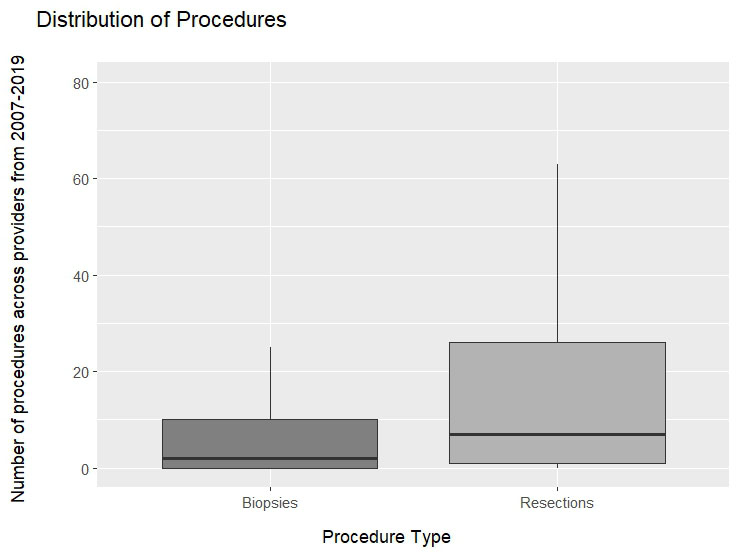

Supplement: Supplementary file 1 — Table S1. Claims codes for surgery. Table S2. Claims codes for chemotherapy. Table S3. IV codes for systemic therapy drugs. Table S4. Claims codes for radiation. Figure S1. Distribution of Center Volume of Neurosurgical Procedure from 2007 to 2019. Outliers are not shown. Of the 810 providers—143 had more than 42 resections (across 2007–2019) and 66 had more than 25 biopsies (across 2007–2019). [file CAM4-14-e70866-s001.docx]
